# Reactions of aluminium(i) with transition metal carbonyls: scope, mechanism and selectivity of CO homologation†

**DOI:** 10.1039/d1sc04940b

**Published:** 2021-10-25

**Authors:** Richard Y. Kong, Maria Batuecas, Mark R. Crimmin

**Affiliations:** Department of Chemistry, Molecular Sciences Research Hub, Imperial College London 82 Wood Lane, Shepherds Bush London W12 0BZ UK m.crimmin@imperial.ac.uk

## Abstract

Over the past few decades, numerous model systems have been discovered that create carbon–carbon bonds from CO. These reactions are of potential relevance to the Fischer–Tropsch process, a technology that converts syngas (H_2_/CO) into mixtures of hydrocarbons. In this paper, a homogeneous model system that constructs carbon chains from CO is reported. The system exploits the cooperative effect of a transition metal complex and main group reductant. An entire reaction sequence from C_1_ → C_2_ → C_3_ → C_4_ has been synthetically verified. The scope of reactivity is broad and includes a variety of transition metals (M = Cr, Mo, W, Mn, Re, Co), including those found in industrial heterogeneous Fischer–Tropsch catalysts. Variation of the transition metal fragment impacts the relative rate of the steps of chain growth, allowing isolation and structural characterisation of a rare C_2_ intermediate. The selectivity of carbon chain growth is also impacted by this variable; two distinct isomers of the C_3_ carbon chain were observed to form in different ratios with different transition metal reagents. Based on a combination of experiments (isotope labelling studies, study of intermediates) and calculations (DFT, NBO, ETS-NOCV) we propose a complete mechanism for chain growth that involves defined reactivity at both transition metal and main group centres.

## Abstract 2

A homogeneous model system that constructs carbon chains from CO is reported. The system exploits the cooperative effect of a transition metal complex and main group reductant. An entire reaction sequence from C_1_ → C_2_ → C_3_ → C_4_ has been synthetically verified.

## Graphical abstract

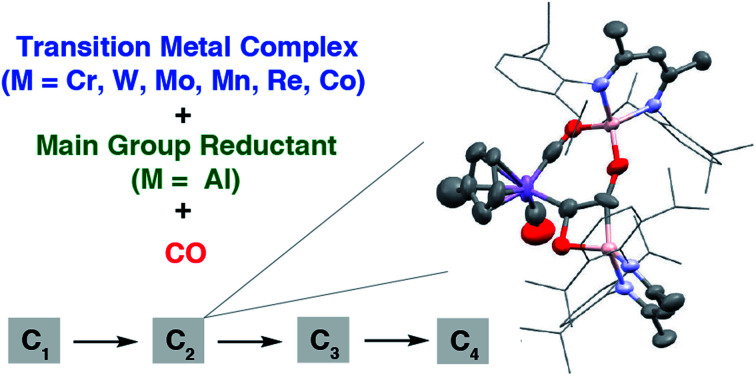

## Introduction

Chemical reactions that allow the formation of useful fine chemicals from C_1_ building blocks such as CO or CO_2_ are of contemporary interest.^1^ These reactions hold promise for sustainable chemical manufacturing methods, as CO can be obtained from renewable sources, including biomass.^2,3^ One approach that has a long history is the Fischer–Tropsch reaction. Implemented on large scales and mediated by heterogeneous transition metal catalysts (typically cobalt, iron, ruthenium), the Fischer–Tropsch process (eqn (1)) allows access to mixtures of hydrocarbons with an Anderson–Schulz–Flory distribution from CO and H_2_.^4^

1 (*n*)CO + (2*n*)H_2_ → C_*n*_H_2*n*_ + (*n*)H_2_O

Homogeneous transition metal complexes have been studied as models of the active sites of Fischer–Tropsch catalysis.^5,6^ While numerous systems have been developed, there is a growing focus on systems in which CO units are reductively combined to make oxygenated chains, {C_*n*_O_*n*_}^*m*−^ (*n* = 2–6).^7–33^ For example, Cloke and coworkers have shown that uranium(iii) compounds can be used to construct carbon chains by combining two (ethynediolate), three (deltate) or four (squarate) CO units in a reductive coupling.^34–36^ Related reactions of low-oxidation state magnesium(i) complexes have been reported by Jones and coworkers. In the absence of a catalyst, three units of CO combine to form a cyclic deltate anion,^37^ while in the presence of 10 mol% of [Mo(CO)_6_] reductive hexamerisation to form a benzenehexolate derived from six units of CO occurred.^38^ Although the direct relevance of these systems to Fischer–Tropsch catalysis remains a point of debate, their formation has captured the imagination of the community. Not least because these systems, and the mechanistic information gained from them, could act as a foundation for the development of homogeneous Fischer–Tropsch catalysts for the selective construction of oxygenated hydrocarbons (*e.g.* polyols) from CO and H_2_.^39^

Despite these advances a clear limitation of the systems reported to date can be identified: in the majority of cases reactions result in the formation of isolable products in which {C_*n*_O_*n*_}^*m*−^ chains coordinate to metal complexes through thermodynamically stable M–O bonds (*e.g.* M = U, Mg). In contrast, cases in which reactive M–C bonds are formed during carbon chain growth from CO are far rarer.^6^ Such systems may offer an opportunity to study the (often opaque) individual steps of carbon chain growth. In addition, although it is known that variation of the transition metal can influence product distributions in heterogeneous Fischer–Tropsch catalysis,^40^ there are few defined examples of organometallic reactions in which multiple carbon chain topologies are accessible from a single reaction.

Recently we reported the reactions of the aluminium(i) reagent [{(ArNCMe)_2_CH}Al] (Ar = 2,6-di-iso-propylphenyl, **[Al]**) with [W(CO)_6_]/CO mixtures.^41^ We documented a system in which {C_*n*_O_*n*_}^4−^ (*n* = 3,4) carbon chains could be synthesised through the cooperative action of the metal complexes. Remarkably these reactions not only proceeded from a defined transition metal carbonyl starting material, but they also led to the isolation of intermediates and products bearing reactive Al–C bonds allowing us, for the first time, to elucidate the mechanism of chain growth from C_1_ → C_3_ → C_4_ species. Herein, we show that the reaction scope can be expanded to a range of transition metal carbonyl precursors. We shed additional light on the mechanism of chain growth through isolation and characterisation of the C_2_ intermediate and demonstrate that systematic variation of the transition metal impacts both the apparent rate and selectivity of the carbon–carbon bond forming steps.

## Results and discussion

### C_1_ to C_4_ chain growth with CO

**Scheme 1:**
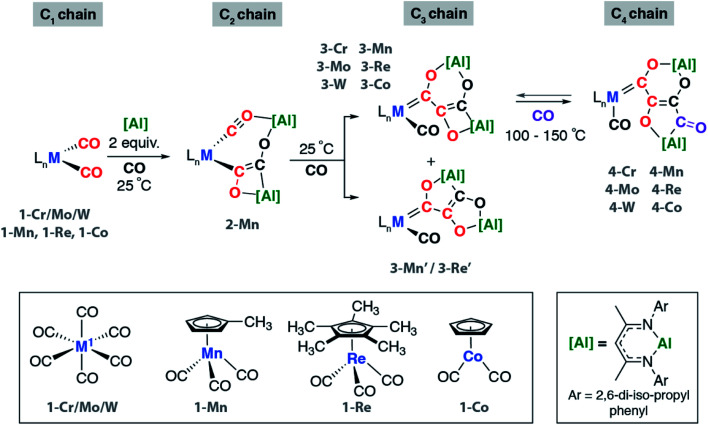
Synthesis of C_2_ to C_4_ carbon chains by CO homologation. Reactions conducted in C_6_D_6_ or C_6_H_6_.

**Fig. 1:**
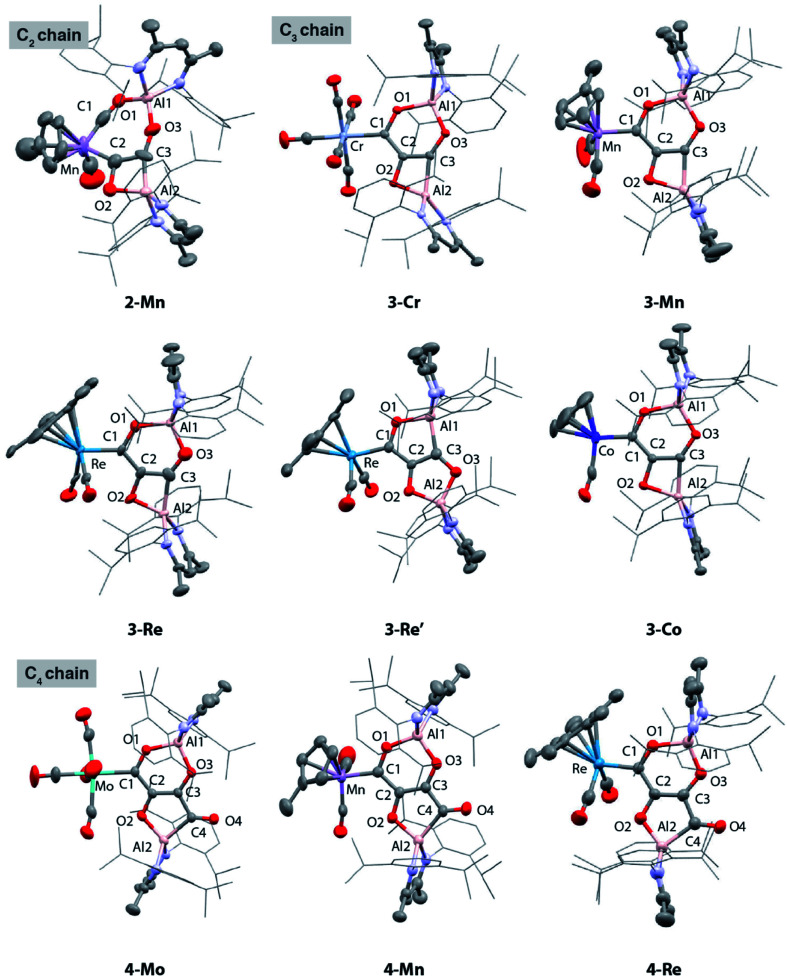
Solid state structures of **2-Mn**, **3-Cr**, **3-Mn**, **3-Re**, **3-Re′**, **3-Co**, **4-Mo**, **4-Mn** and **4-Re**.

We have previously reported the reaction of **[Al]** with [W(CO)_6_] and the isolation of the C_3_ homologation product and its chain growth to a C_4_ analogue. Keen to understand the role of the transition metal and expose the complete mechanism for chain growth from C_1_ to C_4_, we conducted a series of reactions in which the transition metal fragment was varied. Reaction of **[Al]** with a series of group 6–9 metal carbonyl complexes in the presence of CO was investigated (Scheme 1 and Fig. 1). The transition metal complexes were selected based on an 18-electron configuration and low-spin electronic structure. In addition, a *cis*-dicarbonyl motif was considered an essential reactive component for chain growth.

These reactions allowed the isolation of a series of heterometallic products incorporating C_2_, C_3_ or C_4_ carbon chains derived from a 2 : 1 stoichiometry of **[Al]** : **1-M**. The aluminium(i) complex **[Al]** acts as a formal 2-electron reductant in these reactions transferring a total of 4-electrons to the CO derived ligand in the chain growth process. Most notably modification of the electronics at the transition metal appears, qualitatively, to influence the reaction rate of each chain growth step, allowing isolation of a previously unobserved C_2_ intermediate. Hence while reaction of **1-Cr**, **1-Mo**, **1-W**, or **1-Co** with **[Al]** under CO (1 atm.) led directly to the C_3_ products **3-Cr**, **3-Mo**, **3-W**, and **3-Co** at 25 °C, in the case of **1-Mn** conversion to the C_2_ homologue **2-Mn** was achieved.

**2-Mn** is an incredibly rare example of a C_2_ homologation product that contains multiple reactive metal–carbon bonds. Although related complexes have been proposed as reaction intermediates, they are seldom isolated. **2-Mn** could be isolated and shown to convert to **3-Mn** under an atmosphere of CO at 25 °C. A minor isomeric product tentatively assigned as **3-Mn′** was observed alongside **3-Mn** in crude reactions mixtures. **3-Mn** and **3-Mn′** form in a 9 : 1 ratio and likely co-crystallise: **3-Mn** could not be separated from **3-Mn′** by fractional crystallisation. Analogous reactions with the rhenium precursor ultimately shed light on this data. The reaction of **1-Re** with CO formed a 3 : 1 mixture of two products from CO homologation, the expected product **3-Re** which contained [6,4]-fused ring system, alongside **3-Re′** – an isomer with a [5,5]-fused ring system (Scheme 1).^42^ The ratio of **3-Re** : **3-Re′** was unaffected by heating for 18 h at 100 °C. Furthermore, isolated samples of **3-Re** do not convert to mixtures of **3-Re** and **3-Re′**. DFT calculations suggest that **3-Re′** is more stable than **3-Re** by 18.8 kcal mol^−1^, despite forming as the minor product. These data suggest the reactions are under kinetic control with the selectivity determining step occurring during the mechanism of C_1_ to C_3_ chain growth. In all other cases, [6,4]-fused ring systems were identified as the only isomer of the C_3_ chain. This observation along with the variation of product ratios with different transition metal precursors, *i.e.***3-Mn** : **3-Mn′** (9 : 1) and **3-Re** : **3-Re′** (3 : 1), demonstrate that the transition metal fragment impacts the selectivity of the CO homologation process.

Under more forcing conditions (1 atm., 100 °C), **3-Cr**, **3-Mo**, **3-W**, **3-Mn**, and **3-Co** could all be converted to the C_4_ products **4-Cr**, **4-Mo**, **4-W**, **4-Mn**, and **4-Co** respectively. These reactions were found to be reversible and at elevated temperatures (100–150 °C) under a dinitrogen atmosphere the de-insertion of CO could be observed spectroscopically. Mixtures of **3-Re** : **3-Re′** reacted selectively to form **4-Re**. Only **3-Re** was consumed in this reaction suggesting that the strained [6,4] fused ring system is more labile than the [5,5] isomer. **4-Re** could be separated from **3-Re′** through fractional crystallisation. Subsequent heating of **4-Re** under static vacuum to effect CO de-insertion allowed the isolation of pure samples of **3-Re**.

### Multinuclear NMR and infrared characterisation of **2–4**

In C_6_D_6_ solution, **2-Mn** displays ^13^C NMR resonances for the carbonyl and isocarbonyl ligands at *δ* = 236.8 and 189.5 ppm respectively, along with diagnostic resonances for the C_2_ ligand fragment at *δ* = 249.2 and 167.2 ppm. The most deshielded resonance is assigned to the C^2^ position and is consistent with the location of this atomic site adjacent to the transition metal. In the solid-state, the IR spectrum of **2-Mn** shows an absorption at 1921 cm^−1^ assigned to the *ν*(CO) vibrations of the carbonyl ligand. The vibration(s) associated with the isocarbonyl ligand could not be assigned clearly as they overlap with stretches associated with the ligand and are shifted into the fingerprint region. The C_3_ homologation products, **3-Cr**, **3-W**, **3-Mn**, **3-Re** and **3-Co** are characterised by diagnostic ^13^C resonances ranging from *δ* = 265–315 ppm and 167–172 ppm for the C^1^ and C^2^ positions respectively. The largest variation in these data across the series is seen for the C^1^ position as it is connected directly to the transition metal and influenced by the nature of M=C^1^ bond. These chemical shifts are consistent with the assignment as a metallocarbene ligand.^43–45^ Chemical shifts of the C^2^ position are insulated from the transition metal fragment and vary little across the series. In nearly all cases the quadrupolar broadening associated with the ^27^Al nucleus prevents identification of the C^3^ resonance. Despite the different topologies, chemical shifts of the C^1^ and C^2^ position of **3-Re** and **3-Re′** are remarkably similar. Characterisation of ^13^C enriched isotopomers of **3-W** allows aspects of the *J* coupling in the carbon chain to be resolved and the C^3^ resonance to be unambiguously identified. Hence, ^13^CO enriched **3-W** shows the C^3^ resonance at *δ* = 176.5 ppm. The coupling constants in the chain were determined as ^1^*J*_C2–C3_ = 41.8 Hz, ^1^*J*_C1–C2_ = 37.1 Hz, along with ^1^*J*_W–C1_ = 102 Hz determined from natural abundance satellites of ^183^W.

The C_4_ homologation products, **4-W**, **4-Mn** and **4-Re** are characterised by diagnostic ^13^C resonances for the C^1^, C^2^, and C^3^ positions which range from *δ* = 281–349 ppm, 161–169 ppm, and 135–145 ppm respectively. ^13^C enriched samples of **4-W** provide ^13^C–^13^C coupling constants of the carbon chain with ^1^*J*_C1–C2_ = 35.8 Hz, ^1^*J*_C3–C4_ = 27.4 Hz, and ^2^*J*_C2–C4_ = 9.1 Hz. The coupling from W to the C^1^ position of **4-W** is ^1^*J*_W–C1_ = 109 Hz and is similar to the value observed in **3-W**, suggesting little change in the nature of the carbene ligand on elongation of the carbon chain from C_3_ to C_4_.^41^

### Solid-state characterisation of **2–4**

**Fig. 2:**
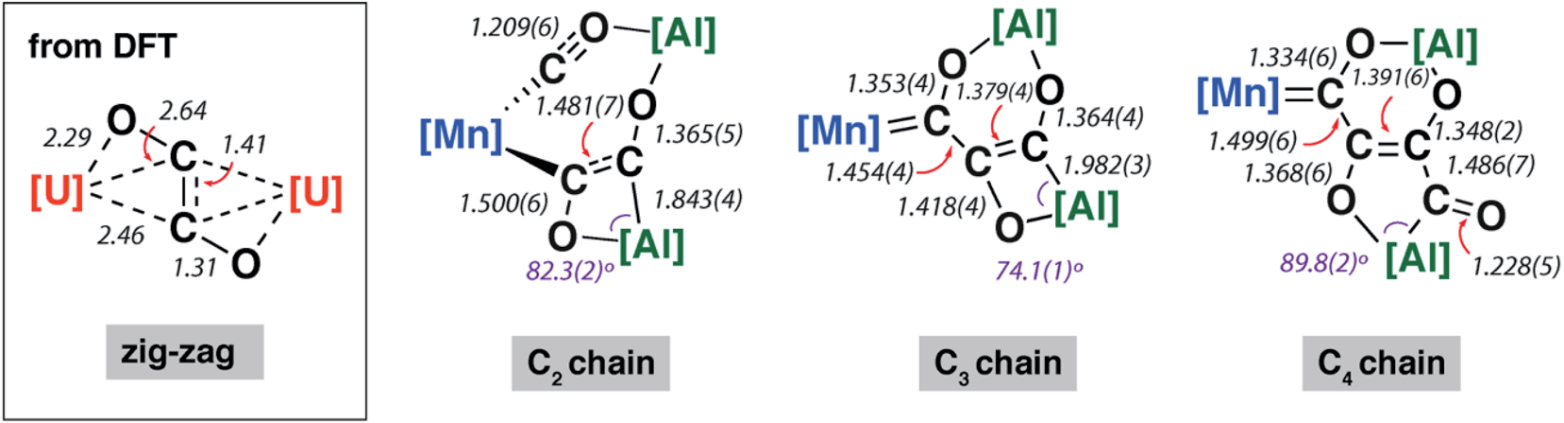
Comparison of key bond lengths and angles (Å or °) in **2-Mn**, **3-Mn** and **4-Mn** alongside a calculated zig-zag intermediate.

The X-ray structure of **2-Mn** incorporates a [7,4] *ortho*-fused ring system. Bond lengths and angles at the main group fragments support the assignment of an aluminium +3 oxidation state.^46^ The 7-membered ring system includes both Mn and Al centres along with the C_2_ fragment and isocarbonyl ligand. The C^1^⋯C^2^ distance is ∼2.6 Å and the system is pre-organised for carbon–carbon bond formation through ring contraction. The C^1^–O^1^ bond length of 1.209(6) Å of the isocarbonyl ligand is ∼0.1 Å longer than the terminal carbonyl of 1.097(8) Å. The C_2_ fragment is bound to Mn through a short Mn–C^2^ bond of 1.971(5) Å. The C^2^–C^3^ distance of 1.481(7) Å is beyond that expected for a C=C bond. Similarly, the C^2^–O^2^ bond of 1.500(6) Å is long. Both these bond lengths shorten upon forming the ring contraction product **3-Mn**. While the C^3^–O^3^ bond length remains near constant in **2-Mn** and **3-Mn** taking values of 1.365(5) and 1.364(4) Å, the Al–C^3^ length is ∼0.1 Å shorter in **2-Mn** than **3-Mn** and the O^2^–Al–C^2^ angle is more obtuse. Both the lengthening of the C≡O bond and the unusual bonding features of the C_2_ fragment are consistent with electron transfer from Al to CO on reaction of **1-Mn**. For comparison, {C_2_O_2_}^2−^ ‘zig-zag’ intermediates have been proposed in the formation {C_2_O_2_}^2−^ ethynediolate and {C_3_O_3_}^2−^ deltate complexes from reaction of CO with uranium(iii) and magnesium(i) compounds.^47^ While neither isolated nor spectroscopically characterised, these species have been inferred by DFT studies. The C_2_ fragment of **2-Mn** is related to these proposed zig-zag intermediates, however the C_2_ ligand in **2-Mn** shows considerable asymmetry and a unique elongated C–O bond that strongly suggests an unusual electronic structure (Fig. 2).

**Table 1:** Selected bond lengths (Å) and angles (°) from solid state data of compounds **2** and **3**

	**2-Mn**	**3-Cr**	**3-Mo** a	**3-W** b	**3-Mn**	**3-Re**	**3-Re′**	**3-Co**
M–C1	1.742(6)	2.055(3)	2.250(4)	2.195(3)	1.922(3)	2.019(6)	2.015(4)	1.810(2)
M–C2	1.971(5)	—	—	—	—	—	—	—
C1⋯C2	2.633(7)	1.445(4)	1.428(6)	1.437(4)	1.454(5)	1.482(9)	1.485(6)	1.447(3)
C2–C3	1.481(7)	1.375(4)	1.399(6)	1.379(4)	1.386(5)	1.382(10)	1.372(7)	1.380(3)
C1–O1	1.209(6)	1.335(3)	1.337(5)	1.337(3)	1.353(4)	1.366(8)	1.344(6)	1.353(3)
C2–O2	1.500(6)	1.412(3)	1.403(5)	1.409(3)	1.418(4)	1.410(9)	1.375(5)	1.409(3)
C3–O3	1.365(5)	1.362(3)	1.328(5)	1.360(3)	1.364(4)	1.321(9)	1.383(6)	1.371(3)
Al–C	1.843(4)	1.979(3)	1.977(4)	1.982(3)	1.942(3)	1.993(8)	1.953(5)	1.946(2)
O–Al2–C	82.25(19)	73.89(10)	74.82(16)	74.13(11)	75.37(13)	73.4(3)	90.02(19)	75.50(9)

a As two molecules were found in the asymmetric unit, data is provided for **3-Mo(B)** one of these molecules.

b Data from ref. 41.

Except for the transition metal fragment, the solid-state structures of **3** and **4** vary only slightly within their series. Key bonding metrics for both the C_3_ and C_4_ carbon chains are consistent, within error, across every single crystal X-ray diffraction study reported herein (Tables 1 and 2). Although we have commented on these types of structures before, some details are included here for clarity. **3** possesses an *ortho*-fused [6,4] ring system derived from contraction of the [7,4] system in **2**, while **4** contains a [6,5] ring system. The side product **3-Re′** contains a [5,5] *ortho*-fused ring system. In all cases, the carbon atoms of both the C_3_ and C_4_ carbon chains are close to idealised sp^2^ hybridised. The C^1^–C^2^ and C^2^–C^3^ bond lengths of **3** range from 1.428(6)–1.482(9) and 1.375(4)–1.399(6) Å respectively; those for **4** are similar. In combination with the C^3^–C^4^ lengths of **4** which vary from 1.486(7)–1.510(5) Å, the data are consistent with an alternating array of C–C and C=C bonds in the carbon chains. The C^1^–O^1^, C^2^–O^2^ and C^3^–O^3^ bond lengths of **3** and **4** range from approximately 1.3 to 1.4 Å and are consistent their assignment as C–O bonds, while the C^4^–O^4^ distances of **4** are shorter at 1.228(5)–1.246(6) Å and assigned as C=O bonds. The bond lengths in **3-Re** and **3-Re′** are remarkably similar, despite the isomerisation of the [6,4] to [5,5] ring system, the major difference is expansion of the O–Al–C angle of the smaller (4- or 5-membered ring) from 73.4(3) to 90.0(2)°. It should be noted, that despite these metrics, the localised resonance structures are an imperfect description as there is the potential for delocalisation of electrons within the {C_3_O_3_}^4−^ and {C_4_O_4_}^4−^ units (*vide infra*). Of the data considered only the M=C^1^ distance shows any real variation across the series. Nevertheless, comparison of **3-W** with **4-W**, **3-Re** with **4-Re**, and **3-Mn** with **4-Mn** shows that there is no change in this bond length, within error, on elongation of the carbon chain from C_3_ to C_4_. This observation is consistent with NMR data and the insensitivity of the ^1^*J*_W–C1_ coupling constants to chain length. Moreover, when considering **3-Cr**, **3-W**, **3-Mo**, **3-Mn**, **3-Re**, and **3-Co** if the covalent radii of the transition metal are factored in, the normalised M=C^1^ bond lengths are remarkably similar with a slight contraction occurring across the period (group 9 < group 7 < group 6).^48^

**Table 2:** Selected bond lengths (Å) and angles (°) from solid state data of compounds **4**

	**4-Cr**	**4-Mo**	**4-W** a	**4-Mn**	**4-Re**
M–C1	2.084(4)	2.231(2)	2.233(4)	1.902(4)	2.030(4)
C1–C2	1.474(5)	1.466(4)	1.457(5)	1.499(6)	1.497(6)
C2–C3	1.374(5)	1.396(3)	1.396(5)	1.391(6)	1.407(7)
C3–C4	1.510(5)	1.499(4)	1.507(5)	1.486(7)	1.494(7)
C1–O1	1.322(4)	1.322(3)	1.322(5)	1.334(6)	1.330(6)
C2–O2	1.370(4)	1.375(3)	1.373(5)	1.368(6)	1.372(6)
C3–O3	1.335(4)	1.337(3)	1.334(5)	1.348(6)	1.353(6)
C4–O4	1.246(5)	1.229(3)	1.233(5)	1.228(5)	1.246(6)
Al–C	1.990(4)	2.002(3)	1.996(4)	2.010(5)	1.990(5)
O–Al2–C	90.19(14)	90.83(10)	90.37(15)	89.81(17)	91.16(18)

a Data from ref. 41.

### Electronic structure of **2–4**

Members of the manganese series (**2-Mn**, **3-Mn**, and **4-Mn**) were interrogated by DFT calculations. Geometries were optimised in the gas-phase using the ωB97x functional with a split 6-31G**(C, H, N, O)/SDDAll (Al, Mn) basis set. These calculations provide insight into the electronic structure of the carbon-chain, how this responds to the number of carbon atoms, and how the carbon chain interacts with the transition metal and main group centres.

NBO calculations (version 6.0) show that the primary interaction between the carbon chains and aluminium atoms is ionic. The NPA charges across all Al sites in **2-Mn**, **3-Mn**, and **4-Mn** range from +2.02 to +2.25. The ionic component to the bonding is further reflected in the large charge localisation on oxygen (−0.86 to −1.05) and carbon (−0.48 to −0.04) atoms directly connected to Al in these complexes. These data are consistent with assignment of a formal +3 oxidation state to aluminium and consideration of the chain as a {C_*n*_O_*n*_}^4−^ (*n* = 2–4) fragment. For **3-Mn** and **4-Mn**, the Wiberg Bond Indices (WBI) between the C–C (C^1^–C^2^ = 1.07, 1.09; C^2^–C^3^ = 1.55, 1.57; C^3^–C^4^ = 1.03) and C–O (C^1^–O^1^ = 1.14, 1.18; C^2^–O^2^ = 0.96, 1.02; C^3^–O^3^ = 1.03, 1.04; C^4^–O^4^ = 1.76) atoms of the {C_*n*_O_*n*_}^4−^ chain reflect the alternating array of single and double bonds suggested by the solid-state structures. While these data hint at a degree of delocalisation of electron density across the 4-, 5- and 6-membered rings of **3-Mn** and **4-Mn**, NICS(0) calculations show that these ring systems are essentially non-aromatic (see ESI†).

**Fig. 3:**
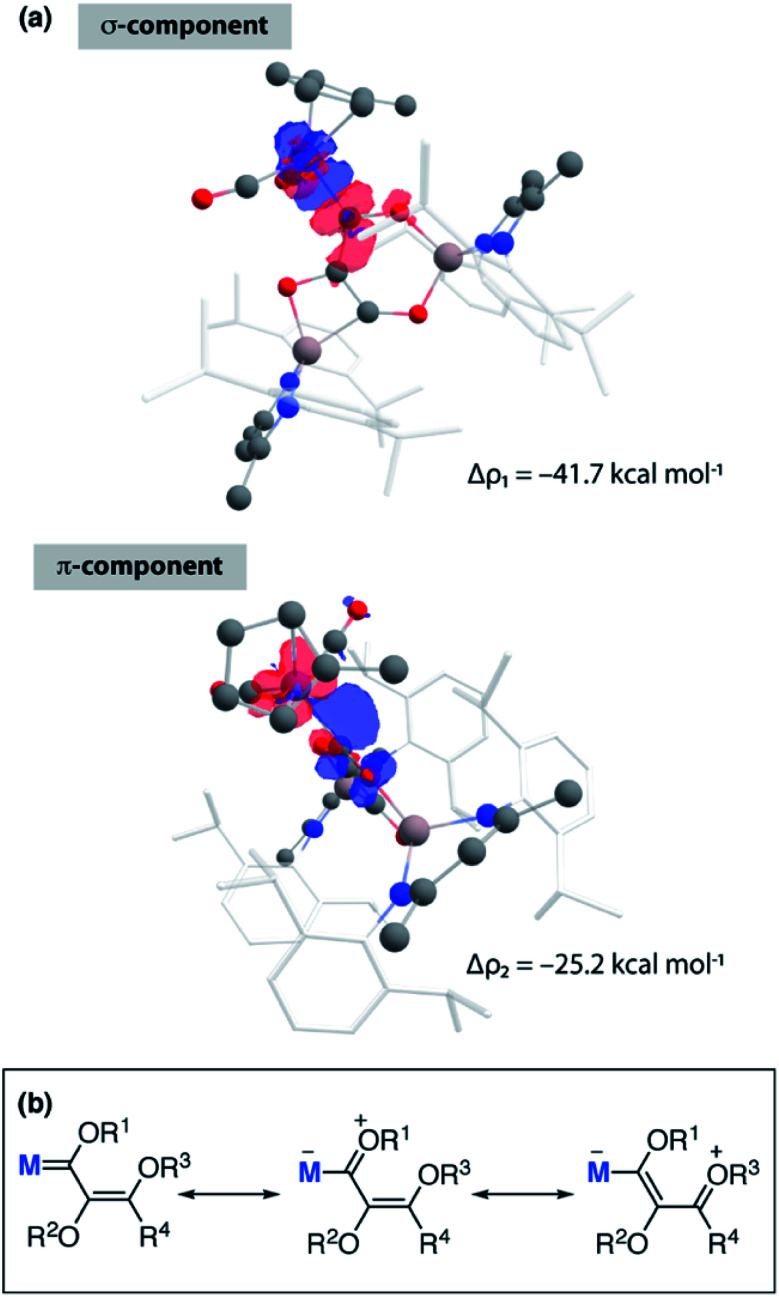
(a) ETS-NOCV calculations on **3-Mn** showing principal components Δ*ρ*_1_ and Δ*ρ*_1_ of Δ*E*_orb_. Charge flow from red to blue. (b) Simplified resonances structures for neutral and zwitterionic bonding components of **3**.

The relatively large WBI for the C^1^–C^2^ and C^1^–O^1^ units of **3-Mn** and **4-Mn** warrants further comment but requires consideration of the nature of M=C^1^ bond to explain. The manganese centre of these complexes bears a slight negative charge (−0.34) while the adjacent carbon atom C^1^ is electropositive (+0.40, +0.42). The calculated WBI for the bond between Mn and C^1^ (0.83) is smaller than that to the carbon atom of the terminal carbonyl ligand (0.95–0.99). The data vary little between **3-Mn** and **4-Mn** and along with the nominal changes in M=C^1^ bond length and ^1^*J*_W–C_ coupling constant strongly suggest that the metal–ligand interaction in these complexes is very similar. ETS-NOCV calculations are consistent with the formalisation of the M=C^1^ interaction of **3-Mn** as a metallocarbene. Extended Transition State Natural Orbitals for Chemical Valence (ETS-NOCV) calculations involve partitioning the molecule into fragments and interrogating the interaction energy between said fragments. From this analysis, the stabilising component from the orbital interaction can be derived and further decomposed into contributions corresponding to pairs of natural orbitals for chemical valence. The total Δ*E*_orb_ of the carbon chain ligand and transition metal fragment of **3-Mn** is −80.1 kcal mol^−1^. There are two principal components Δ*ρ*_1_ (−41.7 kcal mol^−1^) and Δ*ρ*_2_ (−25.2 kcal mol^−1^) comprised of σ-donation from a filled C^1^-based orbital to the metal, along with π-backdonation from a metal d-orbital to a p_*z*_-based orbital on C^1^ (Fig. 3a). A similar bonding situation is found in **4-Mn**. Charge localisation on Mn, along with the large C^1^–O^1^ and C^1^–C^2^ WBIs can be rationalised by a series of zwitterionic resonance structures, reminiscent of those commonly invoked for Fischer carbenes (Fig. 3b).

The electronic structure of **2-Mn** is more complex. While variable temperature NMR data suggests that **2-Mn** may undergo fluxional rearrangement in solution (*vide infra*), there is no evidence this species is paramagnetic. The calculated singlet–triplet gap for **2-Mn** is 38.0 kcal mol^−1^, while such values are known to be functional dependent, the data strongly suggesting a singlet ground state. The Mn–C^2^ WBI in **2-Mn** (0.58) is notably lower than the Mn–C^1^ values in **3-Mn** and **4-Mn**. In combination with the large C^2^–C^3^ WBI (1.79) and increased charge accumulation on C^2^ (+0.15), C^3^ (−0.48), Mn (−0.32) in this complex relative to **3-Mn** and **4-Mn**, these calculations suggest a unique electronic structure of **2-Mn** with the C_2_ ligand adopting some vinyl character. ETS-NOCV calculations support this assertion and capture only a σ-component to the bonding with no clear π-backdonation from the metal to C^2^ (ESI†). The WBIs calculated for the isocarbonyl fragment suggest a lengthened C=O bond (1.38) and compressed M=C bond (1.52) relative to the terminal CO ligand as is typical of bridging isocarbonyl fragments.

### Mechanism of C_1_ to C_4_ chain growth

Preliminary experiments using ^13^C labelled materials [W(^13^CO)_6_] and ^13^CO showed that the first two carbon atoms of **3-W** and **4-W** (C^1^ and C^2^) derive from the transition metal carbonyl, while the second two carbon atoms (C^3^ and C^4^) derive from atmospheric CO. Further, these labelling experiments reveal that an additional equivalent of atmospheric CO is incorporated into the *cis*-position of the transition metal complex during the reaction sequence.^41^ Any plausible mechanism for chain formation must account for these isotopic labelling experiments.

**Fig. 4:**
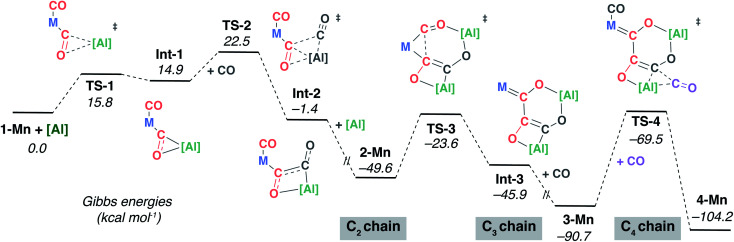
DFT calculated mechanism for C_1_ to C_4_ from **1-Mn**, **[Al]** and CO *via* isolated intermediates **2-Mn**, **3-Mn** and **4-Mn**. M = Mn(η^5^-C_5_H_4_Me)(CO).

The DFT calculations were expanded to consider the formation of the key intermediate **2-Mn** along with the interconversion of **2-Mn** → **3-Mn** → **4-Mn** (Fig. 4). The C_1_ to C_4_ chain-growth mechanism involves alternating reduction and oxidation events at the growing carbon, by **[Al]** and CO respectively. Stationary point geometry optimisation and frequency analysis were performed using the ωB97x functional.^49^ Single point energies were calculated using the M06L^50^ functional with dispersion (D3)^51^ and solvent (PCM, benzene)^52^ corrections. The M06L functional has been previously benchmarked to provide good agreement with experimentally derived thermodynamic parameters on closely related systems.^53^

The calculated pathway begins with nucleophilic attack of **[Al]** onto a C^2^≡O^2^ unit bound to the transition-metal centre, forming **Int-1**.^54,55^ A single transition state connects the starting materials, **1-Mn** and **[Al]**, with **Int-1**. The aluminium centre is bound to both the C^2^ and O^2^ atoms in **Int-1** to form a three-membered ring. Hence, this step could more precisely be described as a (2 + 1) cycloaddition from the perspective of **[Al]**. NBO analysis of **Int-1** shows that 2e^−^ have been transferred from **[Al]** to the CO unit; both the natural charges on C^2^ (−0.11) and O^2^ (−0.85) are lower than that of free CO (+0.69 and −0.52 respectively). Donation of the lone-pair of **[Al]** into the CO π* orbital is a key interaction in **TS-1**.

Subsequent insertion of exogeneous CO into the Al–C bond of **Int-1** is calculated to be facile. This occurs *via* a single transition state, **TS-2**, to form the acyl intermediate **Int-2**. This step of the mechanism is the first carbon–carbon bond forming event, but it constructs the C^2^–C^3^ bond of the chain. The NPA charge analysis shows that insertion of C^3^≡O^3^ into the Al–C^2^ bond of **Int-1** is accompanied by partial oxidation of the carbon chain; both carbon atoms of **Int-2** have positive NPA charges (C^2^ +0.31; C^3^ +0.02). There is literature precedent for the insertion of CO into three-membered aluminocyclopropanes^56^ and aluminocyclopropenes.^57^ Prior calculations from our group have shown that these reactions are reminiscent of migratory insertion of CO at transition metal centres but have a propensity to occur in a concerted manner.^57^ The reaction of a second equivalent of **[Al]** with **Int-2** is calculated to be thermodynamically downhill 
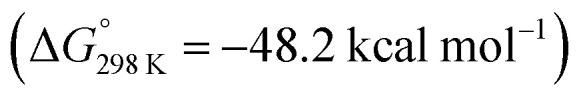
. The NPA charge of the C^2^ and C^3^ carbon in **2-Mn** (+0.15, −0.48) are lower than those in **Int-2** (+0.31, +0.02) reflecting the reduction of the carbon chain in this step. Although a transition state could not be located for this intermolecular process, the step is entirely feasible and leads directly to the key intermediate **2-Mn**, which has been isolated and crystallographically characterised.

The structure of **2-Mn** is preorganised for the migratory insertion of the C^1^≡O^1^ isocarbonyl ligand into the Mn–C^2^ bond. The C^1^–C^2^ bond along with the [6,4] fused ring system of **3-Mn** is formed in this reaction step. A single transition state, **TS-3**, connects **2-Mn** with **Int-3**. The reaction barrier from **2-Mn** to **TS-3** (Δ*G*^‡^_298 K_ = 26.0 kcal mol^−1^) is consistent with this being a slow step, and combined with the relative thermodynamic stability of **2-Mn**, predicts this intermediate to be an isolable species. For comparison, the equivalent step in the pathway from **1-W** is calculated to occur *via* a lower in energy transition state (see ESI†) and all experimental attempts to detect **2-W**, the analogue of **2-Mn**, failed. While it is challenging to comment on the origin of this effect, based on the broader scheme of reactivity (Scheme 1) it does not appear to be steric in origin. One possible explanation for the different reactivity of **2-Mn** and **2-W** is that the migratory insertion step occurs more readily when the C^2^ position is more nucleophilic (see ESI, Fig. S18†). Migratory insertion reactions at transition metal centres are commonly reversible, with trapping of the products by an exogeneous ligand providing a thermodynamic driving force for the forward reaction.^58^ In line with this statement, the formation of **Int-3** from **2-Mn** is calculated to be mildly endergonic 
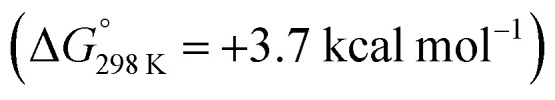
 and likely reversible. Coordination of CO to **Int-3** results in the formation of **3-Mn** and is downhill from **2-Mn**
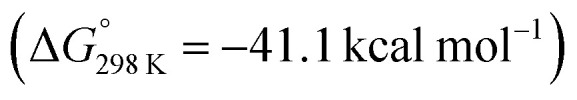
. This coordination event explains the incorporation of exogenous CO into the *cis*-position of the transition metal fragment observed in isotopic labelling studies.

Insertion of a further equivalent of exogeneous C^4^≡O^4^ into the Mn–C^3^ bond of **3-Mn** is calculated to occur by **TS-4** and results in the construction of the C^3^–C^4^ bond and formation of **4-Mn**. The activation barrier and thermodynamics for this step (Δ*G*^‡^_298 K_ = 21.2 kcal mol^−1^; 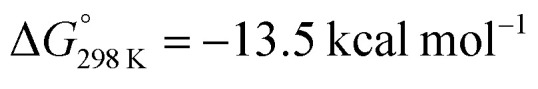) again suggest that **3-Mn** should be isolable. The calculations are also consistent with the reverse reaction, deinsertion of C^4^≡O^4^ from **4-Mn** to form **3-Mn**, being feasible under forcing conditions (Δ*G*^‡^_298 K_ = 34.7 kcal mol^−1^) as observed experimentally.^59^

### Selectivity

**Fig. 5:**
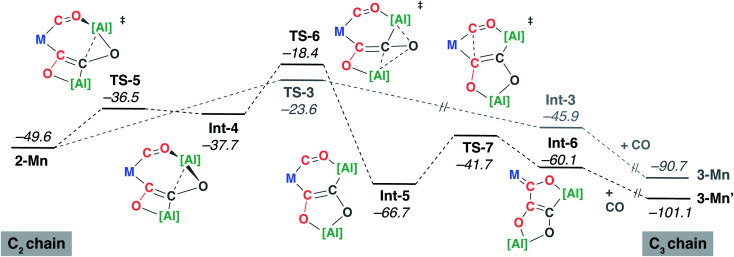
DFT calculated mechanism for the formation of **3-Mn′** from **2-Mn**. M = Mn(η^5^-C_5_H_4_Me)(CO).

A 9 : 1 mixture of **3-Mn** : **3-Mn′** is observed to form upon reaction of **2-Mn** with CO. Hence, the pathway presented in Fig. 4 cannot be the complete mechanistic picture. Based on the experimental findings, the mechanism towards the formation of **3-Mn′** from **2-Mn** was also investigated using DFT calculations (Fig. 5). Isomerisation of **2-Mn** was considered. A low-energy transition state **TS-5** connects **2-Mn** with **Int-4**. This transition state involves migration of the aluminium fragment that is chelated by the isocarbonyl and C_2_ carbon chain from the O^2^ toward the C^2^ atom. The potential energy surface describing this reaction step is calculated to be reasonably flat and **Int-4** is only 1.2 kcal mol^−1^ lower in energy than its associated **TS-5**. The low barrier to the reverse reaction suggests that the isomerisation of **2-Mn** is reversible and by itself is not selectivity determining. Variable temperature ^1^H NMR spectroscopy on d^8^-toluene solution of **2-Mn** across a −60 to +80 °C range revealed a fluxional process involving at least one of the β-diketiminate ligands bound to aluminium. While the complexity of the data does not allow this process to be assigned to a specific molecular reorganisation, the observation is consistent with **2-Mn** being unstable toward either isomerisation or conformational changes below 20 °C.

Subsequent rearrangement of **Int-4** to **Int-5** is calculated to be non-reversible and occurs *via* a single transition state **TS-6**. Like **TS-5**, **TS-6** involves a migration of an aluminium fragment but this time from the C^2^ to the O^2^ atom of the carbon chain. The overall process from **2-Mn** to **Int-5** reorganises the molecular topology converting a [7,4] fused ring system into a [6,5] analogue. Insertion of the C^1^≡O^1^ isocarbonyl ligand into the Mn–C^2^ bond of **Int-5** results in the formation of **Int-6**, in which the [5,5] fused ring system structure of the product is set. Subsequent addition of CO forms **3-Mn′**.

The calculations suggest that the isolated intermediate **2-Mn** provides a point of mechanistic divergence. The formation of **3-Mn** and **3-Mn′** are determined by **TS-3** (Δ*G*^‡^_298 K_ = 26.0 kcal mol^−1^) and **TS-6** (Δ*G*^‡^_298 K_ = 31.2 kcal mol^−1^) respectively. Although the relative energy barriers are consistent with the prediction of **3-Mn** as the major product, closer comparison of the selectivity determining step reveals an energy difference (ΔΔ*G*^‡^_298 K_ = +5.2 kcal mol^−1^) beyond that predicted for a 9 : 1 mixture of products likely highlighting the limitations in accuracy of the DFT model.^60^

## Conclusions

In summary, we have prepared a series of heterometallic products incorporating C_2_, C_3_ or C_4_ chains derived from reaction of group 6–9 metal carbonyl complexes, CO, and an aluminium(i) reductant. These reactions create rare polymetallic complexes in which a {C_*n*_O_*n*_}^4−^ (*n* = 2–4) carbon chain is supported by both transition metal and main group centres. Electronic structures of a manganese series were interrogated by DFT calculations, showing that C_3_ and C_4_ products are best described as metallocarbene complexes of the transition metal. These calculations also suggest that the {C_*n*_O_*n*_}^4−^ chain contains an alternating array of single and double bonds which is bonded to aluminium through polarised and largely ionic Al–O and Al–C bonds. Synthetic studies elucidated each of the steps in the reaction sequence C_2_ → C_3_ → C_4_, confirming the C_2_ product as intermediate in chain growth and showing the step from C_3_ to C_4_ chains is potentially reversible.

Modification of the electronics at the transition metal influences not only the relative rate of each chain growth step allowing but also the selectivity. Through modification of this component, the product distribution of the reaction can be tuned allowing access to different isomers of C_3_ carbon chains which contain different topologies and different reactivity. Further calculations and isotopic labelling experiments were used to gain insight into the mechanism of chain growth. These studies suggest the reaction is initiated by an intermolecular attack of the nucleophilic aluminium(i) reagent at a transition metal carbonyl ligand. Chain growth then proceeds through a series of migratory insertion steps, occurring first at the transition metal site, then at the main group site. The selectivity that controls the chain topology, is influenced by the relative rates of migratory insertion and ligand reorganisation within the heterometallic framework.

In totality, this study provides unprecedented insight into the subtle mechanistic features (*e.g.* reversibility, selectivity, cooperative effects) involved in CO homologation in the presence of transition metal and main group centres.

## Data availability

Primary data (IR, NMR spectra) and computational XYZ coordinates can be downloaded from http://dx.doi.org/10.14469/hpc/8698.

## Author contributions

RYK and MB conducted the experimental work. RYK conducted the DFT calculations. RYK and MB collected and analysed single crystal X-ray diffraction data. MRC managed the project. All authors contributed to writing the manuscript.

## Conflicts of interest

The authors declare no financial conflicts.

## Supplementary Material

SC-012-D1SC04940B-s001

SC-012-D1SC04940B-s002

SC-012-D1SC04940B-s003

SC-012-D1SC04940B-s004
