# Supplementary material for: Reactions of aluminium(i) with transition metal carbonyls: scope, mechanism and selectivity of CO homologation
Source: Chem Sci. 2021 Oct 25;12(44):14845–54. doi: 10.1039/d1sc04940b (PMC8597845; doi:10.1039/d1sc04940b)
Supplement: SC-012-D1SC04940B-s003 [file SC-012-D1SC04940B-s003.pdf]

Electronic Supplementary Material (ESI) for Chemical Science.
This journal is © The Royal Society of Chemistry 2021

Reactions of Aluminium(I) with Transition Metal Carbonyls: Scope, Mechanism and Selectivity of CO Homologation

Electronic Supporting Information

Richard Y. Kong, Maria Batuecas, Mark R. Crimmin

Department of Chemistry, Molecular Sciences Research Hub, Imperial College London, 82 Wood Lane, Shepherds Bush, London, W12 0BZ, UK.

Table of Contents

TABLE OF CONTENTS 2

1 GENERAL EXPERIMENTAL 3

2 SYNTHETIC METHODS 4

2.1 – Preparation of Compounds 4

2.1.1 – Key ^{13}C NMR spectroscopic data of the carbon chain 28

2.1.2 – Synthesis of a ^{13}C labelled sample of 3-W 29

2.2 – VT NMR of 2-Mn 30

3 X-RAY DATA 31

3.1 Normalised M=C for 3 50

4 DENSITY FUNCTIONAL THEORY CALCULATIONS 51

4.1 – Computational methods 51

4.2 –Calculated stationary points 52

4.3 – NBO data 56

4.4 – NICS calculations on 3-Mn and 4-Mn 57

4.5 – ETS-NOCV Calculations 58

4.6 – Functional testing on key stationary points 60

5 COMPUTATIONAL COORDINATES 61

6 REFERENCES 119

1 General Experimental

All manipulations were carried out using standard Schlenk-line and glovebox techniques under an inert atmosphere of argon or dinitrogen. A MBraun Labmaster glovebox was employed, operating at <0.1 ppm O_2 and <0.1 ppm H_2O. A Polar Bear Cub reactor located inside this MBraun Labmaster glovebox was used as the low-temperature reactor. A Grant XUBA1 analogue ultrasonic bath was used to sonicate samples. Solvents were dried over activated alumina from a SPS (solvent purification system) based upon the Grubbs design and degassed before use. Glassware was dried for 12 h at 120 °C prior to use. C_6D_6 was dried over 3 Å molecular sieves and freeze-pump-thaw degassed thrice before use.

NMR Spectra were recorded on Bruker 400 MHz at 25 °C unless otherwise stated and values recorded in ppm. Data were processed in MestReNova software. Where needed, chemical shifts were assigned with the assistance of 2D NMR (HSQC, HMBC, COSY) spectra. **[Al]**[1] and **1-Re**[2] was synthesized according to literature procedures. IR spectra were recorded on an Agilent Cary630 ATR FTIR spectrometer located inside an MBraun glovebox operating at <0.1 ppm O_2 and <0.1 ppm H_2O. Chemicals were purchased from Sigma Aldrich, Fluorochem, Alfa Aesar, or VWR and used as received. CO was purchased from BOC Ltd and used as received. Elemental analyses were performed by Elemental Labs (https://www.elementallab.co.uk/).

All synthetic procedures were run in J-Young NMR tubes. Some syntheses were run in parallel in two separate NMR tubes. This was performed to circumvent the limited headspace (and hence limited CO gas) available to a single NMR tube. Assuming a 2 mL headspace in a standard J-Young NMR tube, 1 bar CO gas pressure, and ideal gas conditions, 0.08 mmol of CO gas can be admitted to a NMR tube. As 2 equiv. of CO gas is required to form **3-M** (and 3 equiv. to form **4-M**), this places a limitation on the scale of the synthesis using a single NMR tube. Upon completion, the reaction mixtures from the two parallel experiments were combined to facilitate isolation.

2 Synthetic Methods

2.1 – Preparation of Compounds

*Preparation of **2-Mn***

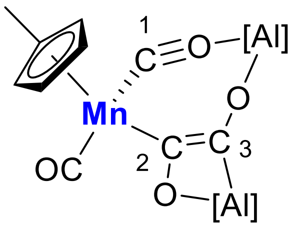

In a glovebox, to a solution of **[Al]** (36 mg, 0.08 mmol. 2 equiv.) in C_6D_6 (1.2 mL) was added [(η^5-C_5H_4Me)Mn(CO)$_3$](8 µL, 11 mg, 0.050 mmol, 1.3 equiv.). The reaction mixture was distributed equally (2x 0.6 mL) into two separate NMR tubes and the headspace of each NMR tube was evacuated *in vacuo*. The NMR tubes were removed from the glovebox, and CO gas (~ 1 bar) was introduced into the tubes. The resultant solutions were sonicated for 15 minutes to ensure the complete dissolution of **[Al]**. At this point, the tubes were shaken several times and allowed to stand for 2.5 h at 25 °C. Both reactions were monitored by ^{1}H NMR spectroscopy and deemed complete upon total consumption of **[Al]**. Upon completion, the NMR tubes were returned to the glovebox, and the combined solutions from both NMR tubes were diluted with toluene (~1 mL) and decanted into in a 20 mL scintillation vial. The resultant dark purple solution was concentrated *in vacuo* until approximately 0.5 mL of solvent remained and filtered into a 4 mL vial. The filtrate was further concentrated until ~0.1 mL of solvent remained. n-Pentane (~2 mL) was layered on top of this solution, and the vial was placed in the glovebox freezer (–35 °C) and **2-Mn** was allowed to crystallise as black-purple blocks. The supernatant was decanted, and the crystals were washed with cold n-pentane thrice (3 x 1mL) before being dried briefly *in vacuo* (~2 min). Yield: 12 mg, 0.012 mmol, 31%.

^{1}H NMR (400 MHz, C_6D_6, 298 K) δ 0.49 (s br, 6H, 2x (C***H*₃**)CH(CH$_3$)), 0.98 (d, $^3J_{HH}$ = 7.0 Hz, 3H, (C$\boldsymbol{H_3}$)CH(CH$_3$)), 1.02 (d, $^3J_{HH}$ = 6.8 Hz, 3H, (C$\boldsymbol{H_3}$)CH(CH$_3$)), 1.05 (d, $^3J_{HH}$ = 6.8 Hz, 3H, (C$\boldsymbol{H_3}$)CH(CH$_3$)), 1.14 (d, $^3J_{HH}$ = 6.9 Hz, 3H, (C$\boldsymbol{H_3}$)CH(CH$_3$)), 1.19 (d, $^3J_{HH}$ = 6.9 Hz, 3H, (C$\boldsymbol{H_3}$)CH(CH$_3$)), 1.23 (d, $^3J_{HH}$ = 6.7 Hz, 3H, (C$\boldsymbol{H_3}$)CH(CH$_3$)), 1.27 (d, $^3J_{HH}$ = 6.1 Hz, 3H, (C$\boldsymbol{H_3}$)CH(CH$_3$)), 1.32 (d, $^3J_{HH}$ = 7.0 Hz, 3H, (C$\boldsymbol{H_3}$)CH(CH$_3$)), 1.35 (s, 3H, (C$\boldsymbol{H_3}$)CH(CH$_3$)), 1.40 (s, 3H, (C$\boldsymbol{H_3}$)C(CH)C(CH$_3$)), 1.47 (d, $^3J_{HH}$ = 6.7 Hz, 3H, (C$\boldsymbol{H_3}$)CH(CH$_3$)), 1.53 (s, 3H, (C$\boldsymbol{H_3}$)C(CH)C(CH$_3$)), 1.54 (s, 3H, (C$\boldsymbol{H_3}$)C(CH)C(CH$_3$)), 1.59 (s, 3H, (C$\boldsymbol{H_3}$)C(CH)C(CH$_3$)), 1.66

(d, $^3J_{HH}$ = 6.5 Hz, 3H, (C$\boldsymbol{H_3}$)CH(CH_3)), 1.72 (s, 3H, Cp'C$\boldsymbol{H_3}$), 2.84 (hept, $^3J_{HH}$ = 6.7 Hz, 1H, (CH_3)C$\boldsymbol{H}$(CH_3)), 2.91-3.26 (overlapping signals, 5H, 5x (CH_3)C$\boldsymbol{H}$(CH_3)), 3.33 (s br, 1H, (CH_3)C$\boldsymbol{H}$(CH_3)), 3.60 (s br, 1H, (CH_3)C$\boldsymbol{H}$(CH_3)), 3.79 (s br, 1H, Cp'C$\boldsymbol{H}$), 3.93 (s br, 1H, Cp'C$\boldsymbol{H}$), 4.25 (s br, 1H, Cp'C$\boldsymbol{H}$), 4.68 (s br, 1H, Cp'C$\boldsymbol{H}$), 4.79 (s, 1H, (CH_3)C(C$\boldsymbol{H}$)C(CH_3)), 4.93 (s, 1H, (CH_3)C(C$\boldsymbol{H}$)C(CH_3)), 6.74 – 7.50 (m overlapping signals, 12H, Ar-$\boldsymbol{H}$).

$^{13}C\{^1H\}$ NMR (101 MHz, C_6D_6, 298 K) δ 14.6 (Cp'$\boldsymbol{C}H_3$), 23.5 (C$\boldsymbol{H_3}$), 23.6 (C$\boldsymbol{H_3}$), 24.0 (C$\boldsymbol{H_3}$), 24.2 (C$\boldsymbol{H_3}$), 24.2 (C$\boldsymbol{H_3}$), 24.5 (C$\boldsymbol{H_3}$), 24.7 (C$\boldsymbol{H_3}$), 24.9 (C$\boldsymbol{H_3}$), 25.1 (C$\boldsymbol{H_3}$), 25.3 (C$\boldsymbol{H_3}$), 26.2 (C$\boldsymbol{H_3}$), 26.3 (C$\boldsymbol{H_3}$), 28.3 ((CH_3)$\boldsymbol{C}$H(CH_3)), 28.7 ((CH_3)$\boldsymbol{C}$H(CH_3)), 28.8 ((CH_3)$\boldsymbol{C}$H(CH_3)), 28.9 ((CH_3)$\boldsymbol{C}$H(CH_3)), 29.0 ((CH_3)$\boldsymbol{C}$H(CH_3)), 29.0 ((CH_3)$\boldsymbol{C}$H(CH_3)), 29.2 ((CH_3)$\boldsymbol{C}$H(CH_3)), 29.3 ((CH_3)$\boldsymbol{C}$H(CH_3)), 84.0 (Cp'$\boldsymbol{C}$H), 86.6 (Cp'$\boldsymbol{C}$H), 89.1 (Cp'$\boldsymbol{C}$H), 91.7 (Cp'$\boldsymbol{C}$H), 98.0 ((CH_3)C($\boldsymbol{C}$H)C(CH_3)), 98.3 ((CH_3)C($\boldsymbol{C}$H)C(CH_3)), 102.7 (Cp'$\boldsymbol{C}CH_3$), 123.5 (Ar$\boldsymbol{C}$), 124.2 (Ar$\boldsymbol{C}$), 124.4 (Ar$\boldsymbol{C}$), 124.6 (Ar$\boldsymbol{C}$), 124.7 (Ar$\boldsymbol{C}$), 124.9 (Ar$\boldsymbol{C}$), 125.2 (Ar$\boldsymbol{C}$), 125.3 (Ar$\boldsymbol{C}$), 125.6 (Ar$\boldsymbol{C}$), 126.9 (Ar$\boldsymbol{C}$), 127.2 (Ar$\boldsymbol{C}$), 139.3 (Ar$\boldsymbol{C}$), 139.6 (Ar$\boldsymbol{C}$), 140.9 (Ar$\boldsymbol{C}$), 141.6 (Ar$\boldsymbol{C}$), 143.7 (Ar$\boldsymbol{C}$), 143.9 (Ar$\boldsymbol{C}$), 144.0 (Ar$\boldsymbol{C}$), 144.3 (Ar$\boldsymbol{C}$), 144.6 (Ar$\boldsymbol{C}$), 145.2 (Ar$\boldsymbol{C}$), 145.8 (Ar$\boldsymbol{C}$), 146.5 (Ar$\boldsymbol{C}$), 167.2 ($\boldsymbol{C^3}$), 169.2 ((CH_3)C($\boldsymbol{C}$H)C(CH_3)), 171.8 ((CH_3)C($\boldsymbol{C}$H)C(CH_3)), 172.7 ((CH_3)C($\boldsymbol{C}$H)C(CH_3)), 172.9 ((CH_3)C($\boldsymbol{C}$H)C(CH_3)), 189.5 ($\boldsymbol{C^1}$), 236.8 (Mn$\boldsymbol{C}$O), 249.2 ($\boldsymbol{C^2}$).

Many methyl and aromatic carbon resonances are broad and overlapping, and could not be observed or identified. $\boldsymbol{C^3}$ is a tentative assignment as it is uncharacteristically sharp for a nucleus bound to quadrupolar ^{29}Al (I = 5/2) nucleus.

IR (ATR), ν_{CO} (cm^{-1}): 1921 (s).

Anal. Calc. ($C_{68}H_{89}Al_2MnN_4O_4$): C, 71.94; H, 7.90; N, 4.93. Found: C, 71.30; H, 7.90; N, 4.76.

Preparation of ***3-Mn***

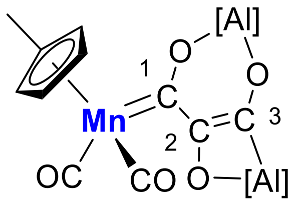

In a glovebox, to a solution of **[Al]** (36 mg, 0.08 mmol. 2 equiv.) in C_6D_6 (1.2 mL) was added [(η^5-C_5H_4Me)Mn(CO)$_3$] (8 µL, 11 mg, 0.050 mmol, 1.3 equiv.). The reaction mixture was distributed equally (2x 0.6 mL) into two separate NMR tubes and the headspace of each NMR tube was evacuated *in vacuo*. The NMR tubes were removed from the glovebox, and CO gas (~ 1 bar) was introduced into the tubes. The resultant solutions were sonicated for 15 minutes to ensure the total dissolution of **[Al]**. At this point, the tubes were shaken several times and allowed to stand for 48 h at 25 °C. The reaction was monitored by ^{1}H NMR spectroscopy and was deemed complete upon total consumption of **2-Mn** and **[Al]**. Both **3-Mn** and **3-Mn'** are observed in the ^{1}H NMR spectrum in a *ca.* 91 : 9 ratio. At this point, the NMR tubes were returned to the glovebox, and the solutions from both NMR tubes were combined and decanted into a 20 mL scintillation vial containing ~5 mL of n-heptane. The resultant dark red solution was concentrated *in vacuo* until precipitate began to form. The solution was filtered and the residue was redissolved in the minimum amount of toluene. The organic fractions were combined. The resultant solution was placed in the glovebox freezer and **3-Mn** was allowed to crystallize at –35 °C as orange-red blocks. The supernatant was decanted, and the resultant crystals were washed with cold n-pentane thrice (3 x 1mL) before being dried briefly *in vacuo* (~2 min). A second crop can be collected from the filtrate by concentrating and crystallizing under identical conditions as above. Yield: 20 mg, 0.017 mmol, 43%.

^{1}H NMR spectroscopy of the single-crystals attained from this preparation showed that a minor product, assigned as **3-Mn'**, co-crystallises with **3-Mn** in an approximately 91 : 9 (**3-Mn** : **3-Mn'**) ratio. **3-Mn** cannot be separated *via* fractional crystallization. **3-Mn'** has been assigned by analogy to **3-Re'** where the latter has been shown to co-crystallise with **3-Re** in a similar fashion.

^{1}H NMR (400 MHz, C_6D_6, 298 K) δ 0.64 (d, $^{3}J_{HH}$ = 6.8 Hz, 6H, $(C\boldsymbol{H_3})_2CH$), 0.70 (d, $^{3}J_{HH}$ = 6.6 Hz, 6H, $(C\boldsymbol{H_3})_2CH$), 0.94 (d, $^{3}J_{HH}$ = 6.8 Hz, 6H, $(C\boldsymbol{H_3})_2CH$), 1.03 (d, $^{3}J_{HH}$ = 6.7 Hz, 6H, $(C\boldsymbol{H_3})_2CH$), 1.07 (d, $^{3}J_{HH}$ = 6.8 Hz, 6H, $(C\boldsymbol{H_3})_2CH$), 1.33 (d, $^{3}J_{HH}$ = 6.8 Hz, 6H, $(C\boldsymbol{H_3})_2CH$), 1.38 (d, $^{3}J_{HH}$ = 6.7 Hz, 6H, $(C\boldsymbol{H_3})_2CH$), 1.41 (s, 6H, $\{(C\boldsymbol{H_3})C\}_2CH$), 1.44 (s, 6H, $\{(C\boldsymbol{H_3})C\}_2CH$), 1.64 (d, $^{3}J_{HH}$ = 6.8 Hz, 6H, $(C\boldsymbol{H_3})_2CH$), 2.02 (s, 3H, Cp'-C$\boldsymbol{H_3}$), 2.93 (hept, $^{3}J_{HH}$ = 6.6 Hz, 2H, 2x $(CH_3)_2C\boldsymbol{H}$), 2.96 (hept, $^{3}J_{HH}$ = 6.8 Hz, 2H, 2x $(CH_3)_2C\boldsymbol{H}$), 3.27 (hept, $^{3}J_{HH}$ = 6.8 Hz, 2H, 2x $(CH_3)_2C\boldsymbol{H}$), 3.40 (hept, $^{3}J_{HH}$ = 6.7 Hz, 2H, 2x $(CH_3)_2C\boldsymbol{H}$), 4.75 (virtual t, 2H, Cp'-C***H***), 4.87 (s, 1H, $\{(CH_3)C\}_2C\boldsymbol{H}$), 4.94 (s, 1H, $\{(CH_3)C\}_2C\boldsymbol{H}$), 5.33 (virtual t, 2H, Cp'-C***H***), 6.78 (dd, $^{3}J_{HH}$ = 5.1, 4.2 Hz, 2H, Ar-***H***), 6.98 – 7.30 (m overlapping signals, 10H, Ar-***H***).

$^{13}C\{^{1}H\}$ NMR (101 MHz, C_6D_6, 298 K) δ 14.5 (Cp'–***C***H_3), 23.6 ($\{(\boldsymbol{C}H_3)C\}_2CH$), 23.6 ($\{(\boldsymbol{C}H_3)C\}_2CH$), 23.8 ($(\boldsymbol{C}H_3)_2CH$), 24.2 ($(\boldsymbol{C}H_3)_2CH$), 24.6 ($(\boldsymbol{C}H_3)_2CH$), 24.8 ($(\boldsymbol{C}H_3)_2CH$), 25.1 ($(\boldsymbol{C}H_3)_2CH$), 25.1 ($(\boldsymbol{C}H_3)_2CH$), 25.7 ($(\boldsymbol{C}H_3)_2CH$), 26.4 ($(\boldsymbol{C}H_3)_2CH$), 28.2 (2x $(CH_3)_2\boldsymbol{C}H$), 28.7 (2x $(CH_3)_2\boldsymbol{C}H$), 28.9 (2x $(CH_3)_2\boldsymbol{C}H$), 29.1 (2x $(CH_3)_2\boldsymbol{C}H$), 84.8 (2x Cp'–***C***H), 87.6 (2x Cp'***C***–H), 98.4 ($\{(CH_3)_2C\}_2\boldsymbol{C}H$), 99.8 ($\{(CH_3)_2C\}_2\boldsymbol{C}H$), 102.1 (Cp'***C***–$CH_3$), 123.7 (Ar***C***), 124.5 (Ar***C***), 125.0 (Ar***C***), 126.1 (Ar***C***), 127.2 (Ar***C***), 129.3 (Ar***C***), 139.1 (Ar***C***), 139.7 (Ar***C***), 142.7 (Ar***C***), 143.1 (Ar***C***), 144.4 (Ar***C***), 146.3 (Ar***C***), 166.7 ($\boldsymbol{C}^2$), 172.3 ($\{(CH_3)_2\boldsymbol{C}\}_2CH$), 172.7 ($\{(CH_3)_2\boldsymbol{C}\}_2CH$), 236.5 ($Mn(\boldsymbol{C}O)_2$, 311.7 ($\boldsymbol{C}^1$).

Some Ar***C*** resonances are overlapping and cannot be observed. The Al–$\boldsymbol{C}^3$ resonance could not be observed in the ^{13}C NMR spectrum due to coupling to the quadrupolar ^{27}Al (I = 5/2) nucleus.

IR (ATR), ν_{CO} (cm^{-1}): 1916 (s), 1847 (s).

Anal. Calc. ($C_{69}H_{89}Al_2MnN_4O_5$): C, 71.24; H, 7.71; N, 4.82. Found: C, 71.98; H, 7.45; N, 4.75.

Preparation of ***4-Mn***

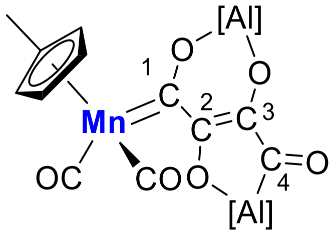

In a glovebox, to an NMR tube charged with **[Al]** (36 mg, 0.08 mmol, 2 equiv.) in C_6D_6 (1.2 mL) was added [(η^5-C_5H_4Me)Mn(CO)$_3$] (8 µL, 11 mg, 0.050 mmol, 1.3 equiv.). The reaction mixture was distributed equally into two separate NMR tubes. The headspace of each NMR tube was evacuated *in vacuo*. The NMR tubes were removed from the glovebox, and CO gas (~ 1 bar) was introduced into the tubes. The resultant solutions were sonicated for 15 minutes to ensure the complete dissolution of **[Al]**. Upon complete dissolution of **[Al]**, the tubes were shaken several times and allowed to stand for 6 h at 25 °C. After 6 h, the tubes were heated at 80°C for 48 h. The CO atmosphere of the tubes was refreshed at the 24 h time interval. The reaction was monitored by ^{1}H NMR spectroscopy and was complete upon consumption of **3-Mn.** The NMR tubes were returned to the glovebox, the headspace evacuated to remove any remaining CO gas, and the solutions from both NMR tubes were combined and decanted into neat n-heptane (~5 mL) in a 20 mL scintillation vial. The resultant dark red solution was concentrated *in vacuo* until precipitated began to form, at which point the solution was filtered and **4-Mn** was allowed to recrystallize at –35 °C in the glovebox freezer as dark red blocks. The supernatant was decanted, and the resultant crystals were washed with cold n-pentane thrice (3 x 1mL) before the crystals were dried briefly *in vacuo* (~2 min). Yield: 24 mg, 0.020 mmol, 50%.

^{1}H NMR (400 MHz, C_6D_6, 298 K) δ 0.57 (d, $^3J_{HH}$ = 6.6 Hz, 6H, (C***H***$_3$)$_2$CH), 0.93 (d, $^3J_{HH}$ = 6.7 Hz, 6H, (C***H***$_3$)$_2$CH), 1.05 (d, $^3J_{HH}$ = 6.9 Hz, 6H, (C***H***$_3$)$_2$CH), 1.08 (d, $^3J_{HH}$ = 6.8 Hz, 6H, (C***H***$_3$)$_2$CH), 1.25 (d, $^3J_{HH}$ = 6.8 Hz, 6H, (C***H***$_3$)$_2$CH), 1.29 (d, $^3J_{HH}$ = 6.6 Hz, 6H, (C***H***$_3$)$_2$CH), 1.41 (s, 6H, {(C***H***$_3$)C}$_2$CH), 1.43 (s, 6H, {(C***H***$_3$)C}$_2$CH), 1.43 (d, $^3J_{HH}$ = 6.7 Hz, 6H, (C***H***$_3$)$_2$CH), 1.48 (d, $^3J_{HH}$ = 6.7 Hz, 6H, (C***H***$_3$)$_2$CH), 1.80 (s, 3H, Cp'-C***H***$_3$), 3.07 (hept, $^3J_{HH}$ = 6.7 Hz, 2H, 2x (CH$_3$)$_2$C***H***), 3.24 (hept, $^3J_{HH}$ = 6.7 Hz, 2H, 2x (CH$_3$)$_2$C***H***), 3.25 (hept overlapping, $^3J_{HH}$ = 6.7 Hz, 2H, 2x (CH$_3$)$_2$C***H***), 3.54 (hept, $^3J_{HH}$ = 6.7 Hz, 2H, 2x (CH$_3$)$_2$C***H***), 4.39 (virtual t, 2H, 2x Cp'-***H***), 4.44 (virtual t, 2H, 2x Cp'-***H***), 4.77 (s, 1H, {(CH$_3$)$_2$C}$_2$C***H***), 4.87 (s, 1H, {(CH$_3$)$_2$C}$_2$C***H***), 7.03 – 7.30 (m, 12H, Ar-***H***).

$^{13}C\{^1H\}$ NMR (101 MHz, C_6D_6, 298 K) δ 13.9 (Cp'**C**H$_3$), 23.4 ({(**C**H$_3$)C}$_2$CH), 23.5 ({(**C**H$_3$)C}$_2$CH), 24.3 ((**C**H$_3$)$_2$CH), 24.3 ((**C**H$_3$)$_2$CH), 24.5 ((**C**H$_3$)$_2$CH), 24.8 ((**C**H$_3$)$_2$CH), 24.9 ((**C**H$_3$)$_2$CH), 24.9 ((**C**H$_3$)$_2$CH), 25.3 ((**C**H$_3$)$_2$CH), 27.0 ((**C**H$_3$)$_2$CH), 28.2 (2x (CH$_3$)$_2$**C**H), 28.4 (2x (CH$_3$)$_2$**C**H), 29.1 (2x (CH$_3$)$_2$**C**H), 29.3 (2x (CH$_3$)$_2$**C**H), 85.1 (2x Cp'**C**H), 88.5 (2x Cp'**C**H), 98.7 ({(CH$_3$)$_2$C}$_2$**C**H), 99.4 ({(CH$_3$)$_2$C}$_2$**C**H), 102.8 (Cp'**C**CH$_3$), 124.3 (2x Ar**C**), 124.8 (Ar**C**), 125.1 (Ar**C**), 127.7 (Ar**C**), 127.9 (Ar**C**), 135.7 (***C^3***), 138.5 (Ar**C**), 139.6 (Ar**C**), 143.2 (Ar**C**), 143.9 (Ar**C**), 145.4 (Ar**C**), 145.4 (Ar**C**), 160.7 (***C^2***), 172.6 ({(CH$_3$)$_2$**C**}$_2$CH), 172.6 ({(CH$_3$)$_2$**C**}$_2$CH), 235.6 (Mn(**C**O)$_2$), 332.3 (***C^1***).

Some Ar**C** resonances are overlapping and cannot be observed. The Al–***C^4*** resonance could not be observed in the ^{13}C NMR spectrum due to coupling to the quadrupolar ^{27}Al (I = 5/2) nucleus.

IR (ATR), ν_{CO} (cm^{-1}): 1920 (s), 1860(s), 1585 (m).

Anal. Calc. ($C_{70}H_{89}Al_2MnN_4O_6$): C, 70.57; H, 7.53; N, 4.70. Found: C, 72.04; H, 7.56; N, 4.26.

Preparation of ***3-Re***

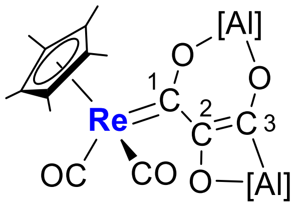

In a glovebox, **4-Re** (24 mg, 0.017 mmol) was suspended in C_6D_6 (0.6 mL) and transferred to a J-Young NMR tube. The tube was heated for 4 days at 100 °C. At 24 h intervals, the headspace of the tube was evacuated *in vacuo* and refilled with N_2. The reaction was monitored by ^{1}H NMR spectroscopy. On complete consumption of **4-Re**, the J-Young NMR tube was returned to the glovebox, the reaction mixture was then diluted with n-heptane (~1 mL), decanted into a 20 mL scintillation vial and concentrated *in vacuo* until precipitation of the product was observed. The reaction mixture was filtered, and any solid residue was dissolved in the minimum amount of C_6H_6, filtered, and combined with the filtrate before the resultant solution was placed in the glovebox freezer. **3-Re** crystallised at –35 °C as orange-red blocks. The supernatant was decanted, and the resultant crystals were washed with cold n-pentane thrice (3 x 1mL) before the crystals were dried briefly *in vacuo* (~2 min). Yield: 8 mg, 0.06 mmol, 35%,

^{1}H NMR (400 MHz, C_6D_6, 298 K) δ 0.59 (d, $^3J_{HH}$ = 6.8 Hz, 6H, $(C\boldsymbol{H}_3)_2CH$), 0.71 (d, $^3J_{HH}$ = 6.7 Hz, 6H, $(C\boldsymbol{H}_3)_2CH$), 0.94 (d, $^3J_{HH}$ = 6.9 Hz, 6H, $(C\boldsymbol{H}_3)_2CH$), 1.07 (d, $^3J_{HH}$ = 6.8 Hz, 6H, $(C\boldsymbol{H}_3)_2CH$), 1.11 (d, $^3J_{HH}$ = 6.9 Hz, 6H, $(C\boldsymbol{H}_3)_2CH$), 1.25 (d, $^3J_{HH}$ = 6.7 Hz, 6H, $(C\boldsymbol{H}_3)_2CH$), 1.37 (d, $^3J_{HH}$ = 6.8 Hz, 6H, $(C\boldsymbol{H}_3)_2CH$), 1.45 (s, 6H, $\{(C\boldsymbol{H}_3)C\}_2CH$), 1.45 (s, 6H, $\{(C\boldsymbol{H}_3)C\}_2CH$), 1.85 (d, $^3J_{HH}$ = 6.8 Hz, 6H, $(C\boldsymbol{H}_3)_2CH$), 2.12 (s, 15H, Cp*$(C\boldsymbol{H}_3)_5$), 3.00 (hept, $^3J_{HH}$ = 6.8 Hz, 2H, 2x $(CH_3)_2C\boldsymbol{H}$), 3.02 (hept, $^3J_{HH}$ = 6.7 Hz, 2H, 2x $(CH_3)_2C\boldsymbol{H}$), 3.09 (hept, $^3J_{HH}$ = 6.7 Hz, 2H, 2x $(CH_3)_2C\boldsymbol{H}$), 3.28 (hept, $^3J_{HH}$ = 6.8 Hz, 2H, 2x $(CH_3)_2C\boldsymbol{H}$), 4.96 (s, 1H, $\{(CH_3)C\}_2C\boldsymbol{H}$), 5.04 (s, 1H, $\{(CH_3)C\}_2C\boldsymbol{H}$), 6.83 (m, 2H, Ar-***H***), 7.05 – 7.38 (m overlapping signals, 10H, Ar-***H***).

$^{13}C\{^1H\}$ NMR (101 MHz, C_6D_6, 298 K) δ 11.2 (Cp*$(\boldsymbol{C}H_3)_5$), 23.4 ($\{(\boldsymbol{C}H_3)C\}_2CH$), 23.5 ($\{(\boldsymbol{C}H_3)C\}_2CH$), 23.8 ($(\boldsymbol{C}H_3)_2CH$), 24.4 ($(\boldsymbol{C}H_3)_2CH$), 24.8 ($(\boldsymbol{C}H_3)_2CH$), 24.9 ($(\boldsymbol{C}H_3)_2CH$), 25.2 ($(\boldsymbol{C}H_3)_2CH$), 25.8 ($(\boldsymbol{C}H_3)_2CH$), 25.8 ($(\boldsymbol{C}H_3)_2CH$), 26.0 ($(\boldsymbol{C}H_3)_2CH$), 28.0 (2x $(CH_3)_2\boldsymbol{C}H$), 28.8 (2x $(CH_3)_2\boldsymbol{C}H$), 28.9 (2x $(CH_3)_2\boldsymbol{C}H$), 29.2 (2x $(CH_3)_2\boldsymbol{C}H$), 98.4 ($\{(CH_3)_2C\}_2\boldsymbol{C}H$), 98.9 (5x Cp*$\boldsymbol{C}CH_3$), 99.3 ($\{(CH_3)_2C\}_2\boldsymbol{C}H$), 124.1 (Ar-***C***), 124.3 (Ar-***C***), 125.3 (Ar-***C***), 126.2 (Ar-***C***), 127.4 (Ar-***C***), 127.5 (Ar-***C***), 139.7 (2x Ar-***C***), 142.8 (Ar-***C***), 142.9 (Ar-***C***), 144.9 (Ar-***C***),

146.0 (Ar-***C***), 170.5 (***C***^{2}), 172.3 ({$(CH_3)_2$***C***}$_2$CH), 172.6 ({$(CH_3)_2$***C***}$_2$CH), 210.3 (Re(***C***O)$_2$), 265.0 (***C***^{1}).

Some Ar***C*** resonances are overlapping and cannot be observed. The Al–***C***^{3} resonance could not be observed in the ^{13}C NMR spectrum due to coupling to the quadrupolar ^{27}Al (I = 5/2) nucleus.

IR (ATR), ν_{CO} (cm^{-1}): 1917 (s), 1848 (s).

Preparation of a mixture of ***3-Re/3-Re'***

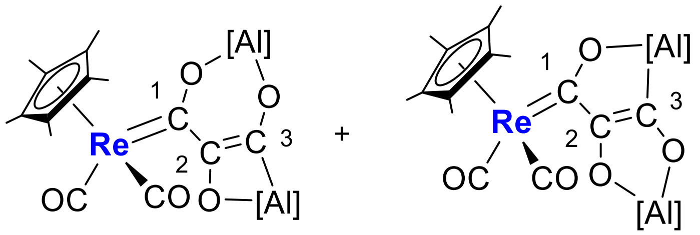

3-Re **3-Re'**

In a glovebox, to a solution of **[Al]** (36 mg, 0.08 mmol, 2 equiv.) in C_6D_6 (0.6 mL) was added a solution of [(η[5]-C_5Me_5)Re(CO)$_3$] (18 mg, 0.044 mmol, 1.1 equiv.) in C_6D_6 (0.6 mL). The reaction mixture was equally divided (2x 0.6 mL) into two J-Young NMR tubes and the headspace of each NMR tube was evacuated *in vacuo*. The NMR tubes were removed from the glovebox, and CO gas (~ 1 bar) was introduced into each of the tubes. The resultant reaction mixtures were sonicated for 15 minutes to ensure the complete dissolution of **[Al]**. At this point, the tubes were shaken several times and allowed to stand for 48 h at 25 °C. The reaction was monitored by ^{1}H NMR spectroscopy and was complete upon complete consumption of **[Al]**. **3-Re** and **3-Re'** were observed to form in ca. 3 : 1 ratio at this timepoint using ^{1}H NMR spectroscopy. The NMR tubes were returned to the glovebox, the headspace evacuated to remove any remaining CO gas, and the solutions from both NMR tubes were combined and decanted into neat n-heptane (~5 mL) in a 20 mL scintillation vial. The resultant dark red solution was concentrated *in vacuo* until approximately 2 mL of solution remains, at which point the solution was filtered into a 4 mL vial. The resultant solution was placed in the glovebox freezer and **3-Re/3Re'** was allowed to crystallise at −35 °C as orange-red blocks. **3-Re** and **3-Re'** co-crystallise and cannot be separated by fractional crystallisation. ^{1}H NMR spectroscopy of single crystals show that the products co-crystallise in a virtually identical ratio to their formation (**3-Re** : **3-Re'** = 3 : 1). The supernatant was decanted, and the resultant crystals were washed with cold n-pentane thrice (3 x 1mL) before being dried briefly *in vacuo* (~2 min). Yield: 33 mg, 0.024 mmol, 61%.

Data for **3-Re** match the data reported above.

*Data for **3-Re'***

^{1}H NMR (400 MHz, C_6D_6, 298 K) δ 0.42 (d, $^3J_{HH}$ = 6.7 Hz, 6H, $(C\boldsymbol{H}_3)_2CH$), 0.96 (d, $^3J_{HH}$ = 6.8 Hz, 6H, $(C\boldsymbol{H}_3)_2CH$), 1.13 (d, $^3J_{HH}$ = 6.8 Hz, 6H, $(C\boldsymbol{H}_3)_2CH$), 1.14 (d, $^3J_{HH}$ = 6.9 Hz, 6H, $(C\boldsymbol{H}_3)_2CH$), 1.24 (d, $^3J_{HH}$ = 6.5 Hz, 6H, $(C\boldsymbol{H}_3)_2CH$), 1.39 (d, $^3J_{HH}$ = 6.7 Hz, 6H, $(C\boldsymbol{H}_3)_2CH$), 1.41 (d, $^3J_{HH}$ = 6.7 Hz, 6H, $(C\boldsymbol{H}_3)_2CH$), 1.44 (s, 6H, $\{(C\boldsymbol{H}_3)C\}_2CH$), 1.44 (s, 6H, $\{(C\boldsymbol{H}_3)C\}_2CH$), 1.89 (d, $^3J_{HH}$ = 6.8 Hz, 6H, $(C\boldsymbol{H}_3)_2CH$), 2.12 (s, 15H, Cp*$(C\boldsymbol{H}_3)_5$), (2.84 – 3.33, m overlapping, 8H, 8x $(CH_3)_2C\boldsymbol{H}$) 4.92 (s, 1H, $\{(CH_3)C\}_2C\boldsymbol{H}$), 5.10 (s, 1H, $\{(CH_3)C\}_2C\boldsymbol{H}$), 6.92 (m, 2H, Ar-***H***), 7.02 – 7.35 (m overlapping signals, 10H, Ar-***H***).

$^{13}C\{^1H\}$ NMR (101 MHz, C_6D_6, 298 K) δ 14.4 (Cp*$(\boldsymbol{C}H_3)_5$, 23.1 ($\{(\boldsymbol{C}H_3)C\}_2CH$), 23.7 ($\{(\boldsymbol{C}H_3)C\}_2CH$), 24.2 ($(\boldsymbol{C}H_3)_2CH$), 24.4 ($(\boldsymbol{C}H_3)_2CH$), 24.5 ($(\boldsymbol{C}H_3)_2CH$), 25.1 ($(\boldsymbol{C}H_3)_2CH$), 25.5 ($(\boldsymbol{C}H_3)_2CH$), 25.6 ($(\boldsymbol{C}H_3)_2CH$), 25.8 ($(\boldsymbol{C}H_3)_2CH$), 29.5 (2x $(CH_3)_2\boldsymbol{C}H$), 29.7 (2x $(CH_3)_2\boldsymbol{C}H$), 32.3 (2x $(CH_3)_2\boldsymbol{C}H$), 98.3 ($\{(CH_3)_2C\}_2\boldsymbol{C}H$), 99.1 (5x Cp*$\boldsymbol{C}CH_3$), 99.8 ($\{(CH_3)_2C\}_2\boldsymbol{C}H$), 124.1 (Ar***C***), 124.7 (Ar***C***), 125.5 (Ar***C***), 126.1 (Ar***C***), 127.4 (Ar***C***), 139.6 (Ar***C***), 140.7 (Ar***C***), 143.0 (Ar***C***), 143.4 (Ar***C***), 143.5 (Ar***C***), 145.9 (Ar***C***), 172.1 ($\{(CH_3)_2\boldsymbol{C}\}_2CH$), 172.3 ($\{(CH_3)_2\boldsymbol{C}\}_2CH$), 178.5 ($\boldsymbol{C}^2$), 210.2 ($Re(\boldsymbol{C}O)_2$), 273.0 ($\boldsymbol{C}^1$).

The spectra for **3-Re'** were measured from a mixture of **3-Re'** and **3-Re**. The $(CH_3)_2C\boldsymbol{H}$ resonances for **3-Re'** in the ^{1}H spectrum overlap with $(CH_3)_2C\boldsymbol{H}$ resonances for **3-Re** and cannot be assigned. Some Ar-***C***, ***C***H_3, and ***C***H resonances overlap with ^{13}C **3-Re** resonances and cannot be identified. The Al–$\boldsymbol{C}^3$ resonance could not be observed in the ^{13}C NMR spectrum due to coupling to the quadrupolar ^{27}Al (I = 5/2) nucleus.

IR (ATR), ν_{CO} (cm^{-1}): 1904 (sh), 1809.

Anal. Calc. ($C_{73}H_{97}Al_2N_4O_5Re$): C, 64.91; H, 7.24; N, 4.15. Found: C, 66.14; H, 7.91; N, 3.97.†

† The high C and H content measured by elemental analysis likely suggests the presence of solvent. One molecule of benzene per molecule of **3-Re/3-Re'** would result in the following elemental analysis: C, 66.41; H, 7.27; N, 3.92.

*Preparation of **4-Re***

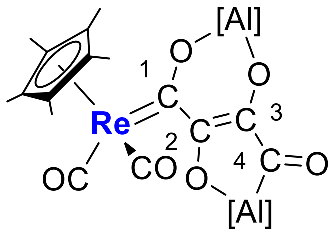

In a glovebox, to an NMR tube charged with **[Al]** (40 mg, 0.090 mmol, 2 equiv.) in C_6D_6 (0.6 mL) was added a solution of [(η^5-C_5Me_5)Re(CO)$_3$] (21 mg, 0.052 mmol, 1.2 equiv.) in C_6D_6 (0.6 mL). The reaction mixture was equally divided (2x 0.6 mL) into two J-Young NMR tubes and the headspace of each NMR tube was evacuated *in vacuo*. The NMR tubes were removed from the glovebox, and CO gas (~ 1 bar) was introduced into the tubes. The tubes were heated at 80°C for 6 h. At this point, the CO atmosphere was refreshed and the tubes heated for a further 48 h at 80°C. The reaction was monitored by ^{1}H NMR spectroscopy. Upon completion complete consumption of **[Al]** and **3-Re** the NMR tubes were returned to the glovebox, the headspace evacuated to remove any remaining CO gas, and the solutions from both NMR tubes were combined and decanted into neat n-heptane (~5 mL) in a 20 mL scintillation vial. The resultant dark red solution was concentrated *in vacuo* until approximately 2 mL of solution remains, at which point the solution was filtered into a 4 mL vial. The resultant solution was placed in the glovebox freezer and **4-Re** was allowed to crystallize at –35 °C as dark red/black blocks. The supernatant was decanted, and the resultant crystals were washed with cold n-pentane thrice (3 x 1mL) before being dried briefly *in vacuo* (~2 min). Yield: 25 mg, 0.018 mmol, 37%.

After recrystallisation, the mother liquor was concentrated *in vacuo* to *ca.* 0.2 mL, layered with pentane (~0.4 mL) and placed in the freezer (–35 °C). Single crystals of **3-Re'** suitable for X-ray diffraction formed as pale-yellow crystals under these conditions. Small amounts of residual **4-Re** also precipitated as a dark red/black solid under these conditions, which could be separated manually on a glass slide from **3-Re'**.

^{1}H NMR (400 MHz, C_6D_6, 298 K) δ 0.76 (d, $^3J_{HH}$ = 6.6 Hz, 6H, (CH_3)$_2$CH), 0.98 (d, $^3J_{HH}$ = 6.7 Hz, 6H, (CH_3)$_2$CH), 1.05 (d, $^3J_{HH}$ = 6.7 Hz, 6H, (CH_3)$_2$CH), 1.06 (d, $^3J_{HH}$ = 6.8 Hz, 6H, (CH_3)$_2$CH), 1.15 (d, $^3J_{HH}$ = 6.8 Hz, 6H, (CH_3)$_2$CH), 1.17 (d, $^3J_{HH}$ = 6.7Hz, 6H, (CH_3)$_2$CH), 1.19 (d, $^3J_{HH}$ = 6.6 Hz, 6H, (CH_3)$_2$CH), 1.39 (s, 6H, {(CH_3)C}$_2$CH), 1.40 (s, 6H, {(CH_3)C}$_2$CH), 1.74

(d, $^3J_{HH}$ = 6.7 Hz, 6H, (C***H***$_3$)$_2$CH), 2.00 (s, 15H, Cp*(***C***H$_3$)$_5$), 3.05 (hept, $^3J_{HH}$ = 6.7 Hz, 2H, 2x (CH$_3$)$_2$C***H***), 3.13 (hept, $^3J_{HH}$ = 6.9 Hz, 2H, 2x (CH$_3$)$_2$C***H***), 3.15 (hept, $^3J_{HH}$ = 6.7 Hz, 2H, 2x (CH$_3$)$_2$C***H***), 3.48 (hept, $^3J_{HH}$ = 6.7 Hz, 2H, 2x (CH$_3$)$_2$C***H***), 4.70 (s, 1H, {(CH$_3$)C}$_2$C***H***), 4.87 (s, 1H, {(CH$_3$)C}$_2$C***H***), 7.08 – 7.28 (m overlapping signals, 12H, Ar-***H***).

^{13}C{^{1}H} NMR (101 MHz, C_6D_6, 298 K) δ 11.1 (Cp*(***C***H$_3$)$_5$), 23.5 ({(***C***H$_3$)$_2$C}$_2$CH), 23.8 ({(***C***H$_3$)$_2$C}$_2$CH), 24.5 ((***C***H$_3$)$_2$CH), 24.6 ((***C***H$_3$)$_2$CH), 25.0 ((***C***H$_3$)$_2$CH), 25.5 ((***C***H$_3$)$_2$CH), 25.7 ((***C***H$_3$)$_2$CH), 25.8 ((***C***H$_3$)$_2$CH), 26.4 ((***C***H$_3$)$_2$CH), 28.1 (2x (CH$_3$)$_2$***C***H), 28.4 (2x (CH$_3$)$_2$***C***H), 28.9 (2x (CH$_3$)$_2$***C***H), 29.5 (2x (CH$_3$)$_2$***C***H), 98.8 ({(CH$_3$)$_2$C}$_2$***C***H), 99.2 ({(CH$_3$)$_2$C}$_2$***C***H), 99.7 (5x Cp****C***CH$_3$), 124.6 (Ar***C***), 124.7 (Ar***C***), 124.8 (Ar***C***), 124.9 (Ar***C***), 127.5 (Ar***C***), 128.7 (Ar***C***), 138.9 (Ar***C***), 139.8 (Ar***C***), 144.0 (Ar***C***), 144.5 (Ar***C***), 144.5 (***C***^{3}), 144.6 (Ar***C***), 144.7 (Ar***C***), 169.1 (***C***^{2}), 172.8 ({(CH$_3$)$_2$***C***}$_2$CH), 173.0 ({(CH$_3$)$_2$***C***}$_2$CH), 209.6 (Re(***C***O)$_2$), 280.6 (***C***^{1}).

Some Ar***C*** resonances are overlapping and cannot be observed. The Al–***C***^{4} resonance could not be observed in the ^{13}C NMR spectrum due to coupling to the quadrupolar ^{27}Al (I = 5/2) nucleus.

IR (ATR), ν_{CO} (cm^{-1}): 1929 (s), 1861 (s), 1584 (m).

Anal. Calc. ($C_{74}H_{97}Al_2N_4O_6Re$): C, 64.46; H, 7.09; N, 4.06. Found: C, 65.63; H, 6.78; N, 3.96.‡

‡ The high C and H content measured by elemental analysis likely suggests the presence of solvent. One molecule of benzene per molecule of **4-Re** would result in the following elemental analysis: C, 65.95; H, 7.13; N, 3.85.

Preparation of ***3-Cr***

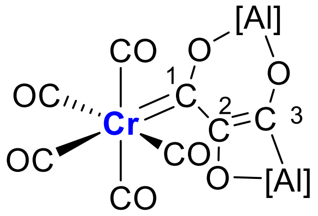

In a glovebox, to an NMR tube charged with a frozen suspension of **[Al]** (18 mg, 0.04 mmol, 2 equiv.) in C_6D_6 (0.3 mL) cooled to –35 °C using a low temperature reactor was added [Cr(CO)$_6$] (5 mg, 0.023 mmol, 1.2 equiv.) slowly as a slurry in C_6D_6 (0.3 mL) *via* Pasteur pipette. Care was taken to ensure that both solutions remain frozen and that the reagents do not mix. The headspace of the NMR tube was evacuated, and the NMR tube was removed from the glovebox quickly and placed into a liquid-nitrogen bath (–196 °C) to keep the two suspensions frozen. The NMR tube was removed from the liquid nitrogen bath and CO gas (~1 bar) was quickly introduced into the headspace of the NMR tube before the frozen solutions began to thaw. The mixture was allowed to thaw, and during this process the tube was shaken vigorously to ensure incorporation of CO gas into solution. The reaction was assumed to be complete once the reaction mixture had completely thawed. At this point, **[Al]** was completely dissolved, and the solution darkens to an orange-brown colour. A precipitate formed. The NMR tube was returned to the glovebox, the headspace of the NMR tube was evacuated to remove the remaining CO gas. The suspension was diluted with ~0.5 mL of toluene and decanted into a 20 mL scintillation vial. The rection mixture was concentrated *in vacuo* until approximately ~0.5 mL of the reaction mixture remained at which point THF was added until all solids were dissolved. The resultant solution was filtered and concentrated *in vacuo* until approximately ~0.6 mL remained. The reaction mixture was placed in the freezer (–35 °C) and **3-Cr** crystallised as bright orange blocks. The crystals were washed thrice with pentane (3 x 1mL) and dried briefly *in vacuo* (~2 min). Yield: 11 mg, 0.009 mmol, 24% yield.

^{1}H NMR (400 MHz, C_6D_6, 298 K) δ 0.51 (d, $^3J_{HH}$ = 6.8 Hz, 6H, (CH_3)$_2$CH), 0.69 (d, $^3J_{HH}$ = 6.7 Hz, 6H, (CH_3)$_2$CH), 0.89 (d, $^3J_{HH}$ = 6.8 Hz, 6H, (CH_3)$_2$CH), 0.99 (d, $^3J_{HH}$ = 6.8 Hz, 6H, (CH_3)$_2$CH), 1.08 (d, $^3J_{HH}$ = 6.8 Hz, 6H, (CH_3)$_2$CH), 1.30 (d, $^3J_{HH}$ = 6.8 Hz, 6H, (CH_3)$_2$CH), 1.33 (d overlapping, $^3J_{HH}$ = 6.7 Hz, (CH_3)$_2$CH), 1.33 (s, 6H, {(CH_3)C}$_2$CH), 1.42 (s, 6H, {(CH_3)C}$_2$CH), 1.70 (d, $^3J_{HH}$ = 6.8 Hz, 6H, (CH_3)$_2$CH), 2.85 (hept, $^3J_{HH}$ = 6.8 Hz, 2H, 2x

$(CH_3)_2C\boldsymbol{H}$), 2.95 (hept, $^3J_{HH}$ = 6.8 Hz, 2H, 2x $(CH_3)_2C\boldsymbol{H}$), 3.15 (hept, $^3J_{HH}$ = 6.8 Hz, 2H, 2x $(CH_3)_2C\boldsymbol{H}$), 3.20 (hept, $^3J_{HH}$ = 6.7 Hz, 2H, 2x $(CH_3)_2C\boldsymbol{H}$), 4.83 (s, 1H, $\{(CH_3)C\}_2C\boldsymbol{H}$), 4.93 (s, 1H, $\{(CH_3)C\}_2C\boldsymbol{H}$), 6.77 (m, 2H, Ar-***H***), 6.96 – 7.35 (m overlapping signals, 10H, Ar-***H***).

$^{13}C\{^1H\}$ NMR (101 MHz, C_6D_6, 298 K) δ 23.4 ($\{(\boldsymbol{C}H_3)C\}_2CH$), 23.5 ($\{(\boldsymbol{C}H_3)C\}_2CH$), 23.7 ($(\boldsymbol{C}H_3)_2CH$), 24.2 ($(\boldsymbol{C}H_3)_2CH$), 24.7 (2x $(\boldsymbol{C}H_3)_2CH$), 24.8 ($(\boldsymbol{C}H_3)_2CH$), 25.0 ($(\boldsymbol{C}H_3)_2CH$), 25.3 ($(\boldsymbol{C}H_3)_2CH$), 26.6 ($(\boldsymbol{C}H_3)_2CH$), 28.1 (2x $(CH_3)_2\boldsymbol{C}H$), 28.8 (2x $(CH_3)_2\boldsymbol{C}H$), 28.9 (2x $(CH_3)_2\boldsymbol{C}H$), 29.0 (2x $(CH_3)_2\boldsymbol{C}H$), 98.6 ($\{(CH_3)_2C\}_2\boldsymbol{C}H$), 100.4 ($\{(CH_3)_2C\}_2\boldsymbol{C}H$), 124.0 (Ar***C***), 124.4 (Ar***C***), 125.4 (Ar***C***), 125.7 (2x Ar***C***), 126.2 (Ar***C***), 138.5 (Ar***C***), 139.1 (Ar***C***), 142.7 (Ar***C***), 142.8 (Ar***C***), 144.4 (Ar***C***), 146.3 (Ar***C***), 169.2 ($\boldsymbol{C^2}$), 172.8 ($\{(CH_3)_2\boldsymbol{C}\}_2CH$), 173.4 ($\{(CH_3)_2\boldsymbol{C}\}_2CH$), 219.9 ($Cr(\boldsymbol{C}O)_4$), 226.2 (Cr***C***O), 314.6 ($\boldsymbol{C^1}$).

Some Ar***C*** resonances are overlapping and cannot be observed. The Al–$\boldsymbol{C^3}$ resonance could not be observed in the ^{13}C NMR spectrum due to coupling to the quadrupolar ^{27}Al (I = 5/2) nucleus.

IR (ATR), ν_{CO} (cm^{-1}): 2042 (m), 1922 (s), 1904 (s), 1886 (s), 1872 (s).

Anal. Calc. ($C_{66}H_{82}Al_2CrN_4O_8$): C, 68.02; H, 7.09; N, 4.81. Found: C, 62.04; H, 6.68; N, 4.62. The low C content, but accurate H and N content likely reflect limitations of the technique (e.g. incomplete C combustion).

Preparation of ***4-Cr***

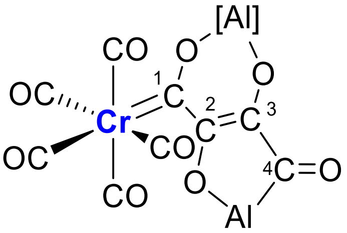

In a glovebox, an NMR tube charged with a frozen suspension of **[Al]** (8.9 mg, 0.02 mmol, 2 equiv.) in C_6D_6 (0.3 mL) was cooled to –35 °C using a low temperature reactor. [Cr(CO)$_6$] (2.2 mg, 0.01 mmol, 1 equiv.) was added slowly as a slurry in C_6D_6 (0.3 mL) *via* Pasteur pipette. Care was taken to ensure that the reaction mixture remained frozen . The headspace of the NMR tube was evacuated, and the NMR tube was removed from the glovebox quickly and placed into a liquid-nitrogen bath (–196 °C). The tube was removed from the liquid nitrogen bath, and CO gas (~1 bar) was introduced into the headspace of the NMR tube while the mixture was still frozen. The mixture was allowed to thaw, and during this process the tube was shaken vigorously to ensure incorporation of CO gas into solution. The resultant mixture was heated at 100 °C and conversion to **4-Cr** was complete after 18 h, as monitored by ^{1}H NMR spectroscopy. The NMR tube was returned to the glovebox and the dark purple reaction mixture was diluted with ~0.5 mL of toluene and decanted into a 20 mL scintillation vial. The resultant solution was concentrated *in vacuo* to ~0.2 mL and placed in the glovebox freezer (–35 °C). **4-Cr** crystallised as purple needles. The supernatant was decanted, and the resultant crystals were washed with cold n-pentane thrice (3 x 0.5mL) before being dried briefly *in vacuo* (~2 min). Yield: 6.2 mg, 0.005 mmol, 52%.

^{1}H NMR (400 MHz, C_6D_6, 298 K) δ 0.36 (d, $^3J_{HH}$ = 6.6 Hz, 6H, (C***H***$_3$)$_2$CH), 0.84 (d, $^3J_{HH}$ = 6.7 Hz, 6H, (C***H***$_3$)$_2$CH), 1.01 (d, $^3J_{HH}$ = 6.7 Hz, 6H, (C***H***$_3$)$_2$CH), 1.07 (d, $^3J_{HH}$ = 6.8 Hz, 6H, (C***H***$_3$)$_2$CH), 1.13 (d, $^3J_{HH}$ = 6.7 Hz, 6H, (C***H***$_3$)$_2$CH), 1.23 (d, $^3J_{HH}$ = 6.7 Hz, 6H, (C***H***$_3$)$_2$CH), 1.38 (s, 6H, {(C***H***$_3$)C}$_2$CH), 1.39 (s, 6H, {(C***H***$_3$)C}$_2$CH), 1.54 (d, $^3J_{HH}$ = 6.6 Hz, 6H, (C***H***$_3$)$_2$CH), 1.64 (d, $^3J_{HH}$ = 6.8 Hz, 6H, (C***H***$_3$)$_2$CH), 2.96 (hept, $^3J_{HH}$ = 6.7 Hz, 2H, (CH$_3$)$_2$C***H***), 3.17 (hept, $^3J_{HH}$ = 6.7 Hz, 2H, (CH$_3$)$_2$C***H***), 3.33 (hept, $^3J_{HH}$ = 6.8 Hz, 2H, (CH$_3$)$_2$C***H***), 3.48 (hept, $^3J_{HH}$ = 6.7 Hz, 2H, (CH$_3$)$_2$C***H***), 4.79 (s, 1H, {(CH$_3$)C}$_2$C***H***), 4.89 (s, 1H, {(CH$_3$)C}$_2$C***H***), 6.94 - 7.27 (m overlapping signals, 12H, Ar-***H***).

$^{13}C\{^1H\}$ NMR (101 MHz, C_6D_6, 298 K) δ 23.4 ({(**C**H$_3$)C}$_2$CH), 23.4 ({(**C**H$_3$)$_2$C}CH), 23.5 ({(**C**H$_3$)$_2$C}$_2$CH), 23.4 ({(**C**H$_3$)$_2$C}$_2$CH), 24.1 (**C**H$_3$)$_2$CH), 24.2 (**C**H$_3$)$_2$CH), 24.2 (**C**H$_3$)$_2$CH), 24.7 (**C**H$_3$)$_2$CH), 24.7 (**C**H$_3$)$_2$CH), 24.8 (**C**H$_3$)$_2$CH), 25.3 (**C**H$_3$)$_2$CH), 27.1, ((**C**H$_3$)$_2$CH), 27.8 (CH$_3$)$_2$**C**H), 28.1 (CH$_3$)$_2$**C**H), 29.4 (CH$_3$)$_2$**C**H), 29.4 (CH$_3$)$_2$**C**H), 98.9 ({(CH$_3$)$_2$C}$_2$**C**H), 99.7 ({(CH$_3$)$_2$C}$_2$**C**H), 124.3 (Ar**C**), 124.7 (Ar**C**), 125.1 (Ar**C**), 125.1 (Ar**C**), 129.3 (Ar**C**), 137.7 (Ar**C**), 139.0 (Ar**C**), 140.1 (**C^3**), 143.5 (Ar**C**), 145,0 (Ar**C**), 145.8 (Ar**C**), 160.8 (**C^2**), 173.0, 173.0, 173.7, 173.7 (all {(CH$_3$)$_2$**C**}$_2$CH), 219.4 (Cr(**C**O)$_4$), 227.8 ((Cr(**C**O)), 348.4 (**C^1**).

Some Ar**C** resonances are overlapping and cannot be observed. The Al–**C^4** resonance could not be observed in the ^{13}C NMR spectrum due to coupling to the quadrupolar ^{27}Al (I = 5/2) nucleus.

IR (ATR), ν_{CO} (cm^{-1}): 2043 (w), 1916 (s), 1886 (s).

Anal. Calc. ($C_{67}H_{82}Al_2CrN_4O_9$): C, 67.43; H, 6.93; N, 4.69. Found: C, 60.64; H, 6.29; N, 4.28. The low C content, but accurate H and N content likely reflect limitations of the technique (e.g. incomplete C combustion).

*Preparation of **3-Mo***

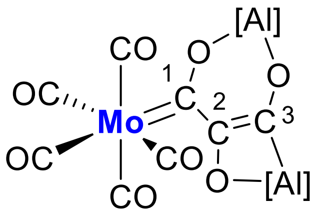

In a glovebox, an NMR tube was charged with a suspension of **[Al]** (18 mg, 0.04 mmol, 2 equiv) in C_6D_6 (0.3 mL), cooled to –35 °C using a low temperature reactor. [Mo(CO)$_6$] (6 mg, 0.023 mmol, 1.2 equiv) was added slowly as a slurry in C_6D_6 (0.3 mL) *via* Pasteur pipette. Care was taken to ensure that the reaction mixture remained frozen. The headspace of the NMR tube was evacuated, and the NMR tube was removed from the glovebox quickly and placed into a liquid-nitrogen bath (–196 °C) to keep the suspension frozen. The tube was removed from the liquid nitrogen bath, and CO gas (~1 bar) was introduced into the headspace of the cold NMR tube while the mixture was still frozen. The mixture was allowed to thaw, and during this process the tube was shaken vigorously to ensure incorporation of CO gas into solution. The reaction was assumed to be complete once the mixture had completely thawed, **[Al]** was completely dissolved, and the solution darkens to an orange-brown colour. The NMR tube was returned to the glovebox, and the solution was diluted with ~0.5 mL of toluene. The resultant solution was concentrated *in vacuo* to ~0.2 mL, filtered, and carefully layered with n-pentane (~1 mL). The vial was placed in the freezer (– 35 °C) and **3-Mo** crystallised as bright orange blocks. Yield: 10 mg, 0.008 mmol, 41% yield.

^{1}H NMR (400 MHz, C_6D_6, 298 K) δ 0.57 (d, $^3J_{HH}$ = 6.7 Hz, 6H, (C***H***$_3$)$_2$CH), 0.61 (d, $^3J_{HH}$ = 6.8 Hz, 6H, (C***H***$_3$)$_2$CH), 0.89 (d, $^3J_{HH}$ = 6.8 Hz, 6H, (C***H***$_3$)$_2$CH), 0.98 (d, $^3J_{HH}$ = 6.8 Hz, 6H, (C***H***$_3$)$_2$CH), 1.07 (d, $^3J_{HH}$ = 6.8 Hz, 6H, (C***H***$_3$)$_2$CH), 1.28 (d, $^3J_{HH}$ = 6.8 Hz, 6H, (C***H***$_3$)$_2$CH), 1.31 (d, $^3J_{HH}$ = 6.7 Hz, 6H, (C***H***$_3$)$_2$CH), 1.34 (s, 6H, {(C***H***$_3$)C}$_2$CH), 1.40 (s, 6H, {(C***H***$_3$)C}$_2$CH), 1.69 (d, $^3J_{HH}$ = 6.7 Hz, 6H, (C***H***$_3$)$_2$CH), 2.87 (hept overlapping, $^3J_{HH}$ = 6.7 Hz, 2H, 2x (CH$_3$)$_2$C***H***), 2.91 (hept overlapping, $^3J_{HH}$ = 6.8 Hz, 2H, 2x (CH$_3$)$_2$C***H***), 3.09 (hept overlapping, $^3J_{HH}$ = 6.8 Hz, 2H, 2x (CH$_3$)$_2$C***H***), 3.12 (hept overlapping, $^3J_{HH}$ = 6.7 Hz, 2H, 2x (CH$_3$)$_2$C***H***), 4.91 (s, 1H, {(CH$_3$)C}$_2$C***H***), 4.93 (s, 1H, {(CH$_3$)C}$_2$C***H***), 6.68 (m, 2H, Ar-***H***), 7.00 – 7.24 (m overlapping signals, 10 H, Ar-***H***).

$^{13}C\{^1H\}$ NMR (101 MHz, C_6D_6, 298 K) δ 23.4 ($\{(\boldsymbol{C}H_3)C\}_2CH$), 23.4 ($\{(\boldsymbol{C}H_3)C\}_2CH$), 23.5 ($(\boldsymbol{C}H_3)_2CH$), 24.1 ($(\boldsymbol{C}H_3)_2CH$), 24.4 ($(\boldsymbol{C}H_3)_2CH$), 24.7 ($(\boldsymbol{C}H_3)_2CH$), 24.8 ($(\boldsymbol{C}H_3)_2CH$), 25.0 ($(\boldsymbol{C}H_3)_2CH$), 26.3 ($(\boldsymbol{C}H_3)_2CH$), 26.7 ($(\boldsymbol{C}H_3)_2CH$), 28.1 (2x $(CH_3)_2\boldsymbol{C}H$), 28.8 (2x $(CH_3)_2\boldsymbol{C}H$), 28.9 (2x $(CH_3)_2\boldsymbol{C}H$), 29.2 (2x $(CH_3)_2\boldsymbol{C}H$), 98.7 ($\{(CH_3)_2C\}_2\boldsymbol{C}H$), 100.2 ($\{(CH_3)_2C\}_2\boldsymbol{C}H$), 123.9 (Ar***C***), 124.3 (Ar***C***), 125.4 (Ar***C***), 126.0 (Ar***C***), 127.5 (Ar***C***), 127.8 (Ar***C***), 138.4 (Ar***C***), 138.9 (Ar***C***), 142.6 (Ar***C***), 142.6 (Ar***C***), 145.0 (Ar***C***), 146.3 (Ar***C***), 170.6 ($\boldsymbol{C}^2$), 172.7 ($\{(CH_3)_2\boldsymbol{C}\}_2CH$), 173.5 ($\{(CH_3)_2\boldsymbol{C}\}_2CH$), 209.7 ($Mo(\boldsymbol{C}O)_4$), 216.1 ($Mo(\boldsymbol{C}O)$), 305.7 ($\boldsymbol{C}^1$).

Some Ar***C*** resonances are overlapping and cannot be observed. The Al–$\boldsymbol{C}^3$ resonance could not be observed in the ^{13}C NMR spectrum due to coupling to the quadrupolar ^{27}Al (I = 5/2) nucleus.

IR (ATR), ν_{CO} (cm^{-1}): 2052 (m), 1935 (s), 1905 (s), 1871 (s).

Anal. Calc. ($C_{66}H_{82}Al_2MoN_4O_8$): C, 65.55; H, 6.83; N, 4.63. Found: C, 65.83; H, 7.09; N, 5.05.

Preparation of ***4-Mo***

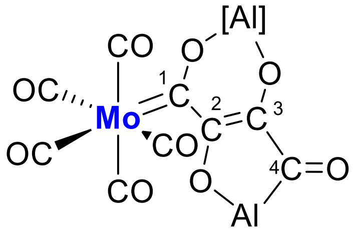

In a glovebox, a NMR tube was charged with a suspension of **[Al]** (8.9 mg, 0.02 mmol, 2 equiv) in C_6D_6 (0.3 mL) was cooled to –35 °C using a low temperature reactor. [Mo(CO)$_6$] (2.5 mg, 0.01 mmol, 1 equiv) was added slowly as a slurry in C_6D_6 (0.3 mL) *via* Pasteur pipette. Care was taken to ensure that the reaction mixture remains frozen. The headspace of the NMR tube was evacuated, and the NMR tube was removed from the glovebox quickly and placed into a liquid-nitrogen bath (–196 °C). The tube was removed from the liquid nitrogen bath, and CO gas (~1 bar) was introduced into the headspace of the NMR tube while the mixture was still frozen. Upon addition of CO gas, the mixture was allowed to thaw, and during this process the tube was shaken vigorously to ensure incorporation of CO gas into solution. The resultant mixture was heated at 100 °C and conversion to **4-Mo** was complete after 18 h, as monitored by ^{1}H NMR spectroscopy. The NMR tube was returned to the glovebox, the headspace of the NMR tube was evacuated to remove the remaining CO gas, and the dark purple reaction mixture was diluted with ~0.5 mL of toluene and decanted into a 20 mL scintillation vial. The resultant solution was concentrated *in vacuo* to ~0.2 mL, filtered into a 4 mL vial, and carefully layered with n-pentane (~2 mL). The vial was placed in the glovebox freezer (-35 °C) and **4-Mo** crystallised as red blocks. The supernatant was decanted, and the resultant crystals were washed with cold n-pentane thrice (3 x 0.5mL) before being dried briefly *in vacuo* (~2 min). Yield: 5.6 mg, 0.004 mmol, 45%.

^{1}H NMR (400 MHz, C_6D_6, 298 K) δ 0.35 (d, $^3J_{HH}$ = 6.6 Hz, 6H, (C***H***$_3$)$_2$CH), 0.79 (d, $^3J_{HH}$ = 6.7 Hz, 6H, (C***H***$_3$)$_2$CH), 1.01 (d, $^3J_{HH}$ = 6.7 Hz, 12H, (C***H***$_3$)$_2$CH), 1.08 (d, $^3J_{HH}$ = 6.8 Hz, 6H, (C***H***$_3$)$_2$CH), 1.25 (d, $^3J_{HH}$ = 6.7 Hz, 6H, (C***H***$_3$)$_2$CH), 1.40 (s, 6H, {(C***H***$_3$)C}$_2$CH), 1.41 (s, 6H, {(C***H***$_3$)C}$_2$CH), 1.57 (d, $^3J_{HH}$ = 6.7 Hz, 6H, (C***H***$_3$)$_2$CH), 1.70 (d, $^3J_{HH}$ = 6.8 Hz, 6H, (C***H***$_3$)$_2$CH), 2.97 (hept, $^3J_{HH}$ = 6.7 Hz, 2H, (CH$_3$)$_2$C***H***), 3.12 (hept, $^3J_{HH}$ = 6.8 Hz, 2H, (CH$_3$)$_2$C***H***), 3.24 (hept, $^3J_{HH}$ = 6.7 Hz, 2H, (CH$_3$)$_2$C***H***), 3.53 (hept, $^3J_{HH}$ = 6.7 Hz, 2H, (CH$_3$)$_2$C***H***), 4.86 (s, 1H, {(CH$_3$)C}$_2$C***H***), 4.92 (s, 1H, {(CH$_3$)C}$_2$C***H***), 6.94 - 7.23 (m overlapping signals, 12H, Ar-***H***).

$^{13}C\{^{1}H\}$ NMR (101 MHz, C_6D_6, 353 K) δ 23.3 ($\{(\boldsymbol{C}H_3)C\}_2CH$), 23.5 ($\{(\boldsymbol{C}H_3)C\}_2CH$), 24.1 ($(\boldsymbol{C}H_3)_2CH$), 24.3 ($(\boldsymbol{C}H_3)_2CH$), 24.4 ($(\boldsymbol{C}H_3)_2CH$), 24.7 ($(\boldsymbol{C}H_3)_2CH$), 24.8 ($(\boldsymbol{C}H_3)_2CH$), 24.9 ($(\boldsymbol{C}H_3)_2CH$), 25.8 ($(\boldsymbol{C}H_3)_2CH$), 27.0 ($(\boldsymbol{C}H_3)_2CH$), 28.0 ($(CH_3)_2\boldsymbol{C}H$), 28.1 ($(CH_3)_2\boldsymbol{C}H$), 29.4 ($(CH_3)_2\boldsymbol{C}H$), 29.4 ($(CH_3)_2\boldsymbol{C}H$), 98.7 ($\{(CH_3)_2C\}_2\boldsymbol{C}H$), 99.7 ($\{(CH_3)_2C\}_2\boldsymbol{C}H$), 124.3 (Ar***C***), 124.8 (Ar***C***), 125.1 (Ar***C***), 125.2 (Ar***C***), 137.8, 139.2 (Ar***C***), 143.9 (Ar***C***), 144.0 (Ar***C***), 144.7 ($\boldsymbol{C}^{3}$), 145.3 (Ar***C***), 146.3 (Ar***C***), 161.7 ($\boldsymbol{C}^{2}$), 173.2 ($\{(CH_3)_2\boldsymbol{C}\}_2CH$), 173.2 ($\{(CH_3)_2\boldsymbol{C}\}_2CH$), 173.8 ($\{(CH_3)_2\boldsymbol{C}\}_2CH$), 173.8 ($\{(CH_3)_2\boldsymbol{C}\}_2CH$), 209.3 ($Mo(\boldsymbol{C}O)_4$), 216.6 ($Mo(\boldsymbol{C}O)$), 348.8 ($\boldsymbol{C}^{1}$).

Some Ar***C*** resonances are overlapping and cannot be observed. The Al–$\boldsymbol{C}^{4}$ resonance could not be observed in the ^{13}C NMR spectrum due to coupling to the quadrupolar ^{27}Al (I = 5/2) nucleus.

IR (ATR), ν_{CO} (cm^{-1}): 2050 (m),1916 (s), 1895 (s).

Anal. Calc. ($C_{67}H_{82}Al_2MoN_4O_9$): C, 65.04; H, 6.68; N, 4.53. Found: C, 65.03; H, 6.20; N, 4.10.

*Preparation of **3-Co***

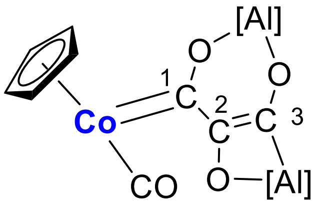

In a glovebox, a solution of **[Al]** (36 mg, 0.08 mmol, 2 equiv) in C_6D_6 (0.6 mL) was equally divided into two separate J-Young NMR tubes. The solutions were frozen using a low-temperature reactor cooled to –35 °C. A solution of [(η^5-C_5H_5)Co(CO)$_2$] (6.0 µL, 8.2 mg, 0.044 mmol, 1.1 equiv) in C_6D_6 (0.6 mL) was divided into two portions (2x 0.3 mL) and one portion was added to each NMR tube. Care was taken to ensure that the reaction mixture in each NMR tube remained frozen. The headspace of each NMR tube was evacuated, and the NMR tubes were removed from the glovebox quickly and placed into a liquid-nitrogen bath (–196 °C). The NMR tubes were removed from the liquid nitrogen bath and CO gas (~ 1 bar) was introduced into the tubes while the mixture was still frozen. Upon addition of CO gas, the reaction mixture was allowed to thaw, and during this process the tube was shaken vigorously to ensure incorporation of CO gas into solution. The reaction was assumed complete upon the formation of a dark homogeneous solution. The NMR tubes were returned to the glovebox, and the solutions from both NMR tubes were combined and into a 20 mL scintillation vial containing ~5 mL of n-heptane. The resultant solution was concentrated *in vacuo* until a precipitate began to form, at which point the solution was filtered. The residue was redissolved in the minimum amount of toluene (< 0.1 mL) and filtered. All organic fractions were combined. The resultant solution was placed in the glovebox freezer and **3-Co** was allowed to crystallise at –35 °C as dark purple-red crystals. The supernatant was decanted, and the resultant crystals were washed with cold n-pentane thrice (3 x 1mL) before the crystals were dried briefly *in vacuo* (~2 min). Yield: 21 mg, 0.019 mmol, 48%.

^{1}H NMR (400 MHz, C_6D_6, 298 K) δ 0.80 (d, $^3J_{HH}$ = 6.8 Hz, 6H, (C$\boldsymbol{H}_3$)$_2$CH), 0.84 (s br, 6H, (C$\boldsymbol{H}_3$)$_2$CH), 1.00 (d, $^3J_{HH}$ = 6.9 Hz, 6H, (C$\boldsymbol{H}_3$)$_2$CH), 1.03 (d, $^3J_{HH}$ = 6.8 Hz, 6H, (C$\boldsymbol{H}_3$)$_2$CH), 1.06 (d, $^3J_{HH}$ = 6.8 Hz, 6H, (C$\boldsymbol{H}_3$)$_2$CH), 1.27 (s br, 6H, (C$\boldsymbol{H}_3$)$_2$CH), 1.31 (d, $^3J_{HH}$ = 6.8 Hz, 6H, (C$\boldsymbol{H}_3$)$_2$CH), 1.41 (s, 6H, {(C$\boldsymbol{H}_3$)C}$_2$CH), 1.47 (s, 6H, {(C$\boldsymbol{H}_3$)C}$_2$CH), 1.76 (s br, 6H, (C$\boldsymbol{H}_3$)$_2$CH), 2.86 (hept, $^3J_{HH}$ = 6.7 Hz, 2H, 2x (CH$_3$)$_2$C$\boldsymbol{H}$), 2.89 (hept, $^3J_{HH}$ = 6.8 Hz, 2H, 2x (CH$_3$)$_2$C$\boldsymbol{H}$), 3.28 (hept, $^3J_{HH}$ = 6.7 Hz, 2H, 2x (CH$_3$)$_2$C$\boldsymbol{H}$), 3.41 (hept, $^3J_{HH}$ = 6.7 Hz, 2H, 2x (CH$_3$)$_2$C$\boldsymbol{H}$),

4.87 (s, 1H, {(CH3)C}2C***H***), 4.94 (s, 1H, {(CH3)C}2C***H***), 5.02 (s, 5H, Cp-***H***), 6.59 – 6.78 (m, 2H, Ar-***H***), 7.00-7.25 (m overlapping signals, 10H, Ar-***H***).

$^{13}C\{^{1}H\}$ NMR (101 MHz, C_6D_6, 298 K) δ 23.2 ({(***C***H3)C}2CH), 23.4 ({(***C***H3)C}2CH), 24.4 ((***C***H3)2CH), 24.6 ((***C***H3)2CH), 24.6 ((***C***H3)2CH), 24.9 (2x (***C***H3)2CH), 24.9 ((***C***H3)2CH), 26.5 ((***C***H3)2CH), 27.4 ((***C***H3)2CH), 28.6 (2x (CH3)2***C***H), 28.2 (2x (CH3)2***C***H), 28.8 (2x (CH3)2***C***H), 29.3 (2x (CH3)2***C***H), 85.1 (5x Cp'***C***H), 98.0 ({(CH3)2C}2***C***H), 98.7 ({(CH3)2C}2***C***H), 123.6 (Ar***C***), 124.0 (Ar***C***), 125.0 (Ar***C***), 125.9 (Ar***C***), 127.1 (Ar***C***), 128.6 (Ar***C***), 138.5 (Ar***C***), 139.4 (Ar***C***), 142.4 (Ar***C***), 142.9 (Ar***C***), 145.2 (Ar***C***), 145.8 (Ar***C***), 171.7 ({(CH3)2***C***}2CH), 172.1 ({(CH3)2***C***}2CH).

Some Ar***C*** resonances are overlapping and cannot be observed. The Al–$\boldsymbol{C^3}$ and Co–$\boldsymbol{C^1}$ resonance could not be observed in the ^{13}C NMR spectrum due to coupling to the quadrupolar ^{27}Al (I = 5/2) and ^{59}Co (I = 7/2) nuclei respectively. The low solubility of **3-Co** in C_6D_6 precluded observation of the $\boldsymbol{C^2}$ resonance.

IR (ATR), ν_{CO} (cm^{-1}): 1938 (s).

Anal. Calc. ($C_{67}H_{87}Al_2CoN_4O_4$): C, 71.51; H, 7.79; N, 4.98. Found: C, 68.63; H, 7.44; N, 5.07. The low C content, but accurate H and N content likely reflect limitations of the technique (e.g. incomplete C combustion).

*Preparation of **4-Co***

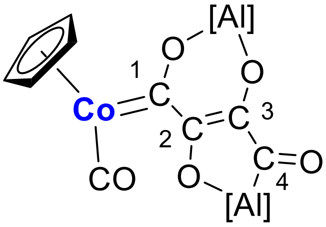

In a glovebox, to an NMR tube charged with a frozen suspension of **[Al]** (26 mg, 0.058 mmol, 2 equiv) in C_6D_6 (0.3 mL) was added a solution of [(η^5-C_5H_5)Co(CO)$_2$] (4.0 µL, 5.5 mg, 0.029 mmol, 1 equiv) in C_6D_6 (0.3 mL). Care was taken to ensure that the reaction mixture remained frozen and that the reagents do not mix. The headspace of the NMR tube was evacuated, and the NMR tube was removed from the glovebox quickly and placed into a liquid-nitrogen bath (–196 °C) to keep the suspension frozen. The NMR tube was removed from the liquid nitrogen bath and CO gas (~ 1 bar) was introduced while the mixture was still frozen. Upon addition of CO gas, the mixture was allowed to thaw, and during this process the tube was shaken vigorously to ensure incorporation of CO gas into solution. The CO gas atmosphere of the NMR tube was refreshed at this timepoint. - The NMR tube was then heated at 100°C for 18 h. The NMR tube was returned to the glovebox, diluted with toluene (~0.6 mL). The reaction mixture was concentrated *in vacuo* until ~0.3 mL of the solution remained, at which point the solution was filtered and n-pentane (~2 mL) was layered on top of the filtrate. The resultant solution was placed in the glovebox freezer and **4-Co** was allowed to crystallize at –35 °C as black needles. The supernatant was decanted, and the resultant crystals were washed with cold n-pentane thrice (3 x 1mL) before the crystals were dried briefly *in vacuo* (~2 min). Yield: 18 mg, 0.016 mmol, 54%.

^{1}H NMR (400 MHz, C_6D_6, 298 K) δ 0.37 (d, $^3J_{HH}$ = 6.6 Hz, 6H, (C$\boldsymbol{H}_3$)$_2$CH), 0.87 (d, $^3J_{HH}$ = 6.7 Hz, 6H, (C$\boldsymbol{H}_3$)$_2$CH), 1.07 (d, $^3J_{HH}$ = 6.5 Hz, 6H, (C$\boldsymbol{H}_3$)$_2$CH), 1.08 (d, $^3J_{HH}$ = 6.8 Hz, 6H, (C$\boldsymbol{H}_3$)$_2$CH), 1.08 (d, $^3J_{HH}$ = 6.7 Hz, 6H, (C$\boldsymbol{H}_3$)$_2$CH), 1.35 (d, $^3J_{HH}$ = 6.8 Hz, 6H, (C$\boldsymbol{H}_3$)$_2$CH), 1.45 (s, 6H, {(C$\boldsymbol{H}_3$)C}$_2$CH), 1.46 (s, 6H, {(C$\boldsymbol{H}_3$)C}$_2$CH), 1.61 (d, $^3J_{HH}$ = 6.8 Hz, 6H, (C$\boldsymbol{H}_3$)$_2$CH), 1.66 (d, $^3J_{HH}$ = 6.7 Hz, 6H, (C$\boldsymbol{H}_3$)$_2$CH), 3.08 (hept, $^3J_{HH}$ = 6.6 Hz, 2H, (CH$_3$)$_2$C$\boldsymbol{H}$), 3.18 (hept, $^3J_{HH}$ = 6.8 Hz, 2H, (CH$_3$)$_2$C$\boldsymbol{H}$), 3.22 (hept, $^3J_{HH}$ = 6.6 Hz, 2H, (CH$_3$)$_2$C$\boldsymbol{H}$), 3.65 (hept, $^3J_{HH}$ = 6.6 Hz, 2H, (CH$_3$)$_2$C$\boldsymbol{H}$), 4.64 (s, 5H, 5x **Cp-H**), 4.78 (s, 1H, {(CH$_3$)C}$_2$C$\boldsymbol{H}$), 4.93 (s, 1H, {(CH$_3$)C}$_2$C$\boldsymbol{H}$), 7.00 – 7.31 (m, 12H, Ar-$\boldsymbol{H}$).

$^{13}C\{^1H\}$ NMR (101 MHz, C_6D_6, 298 K) δ 23.2 ($\{(\boldsymbol{C}H_3)_2C\}_2CH$), 23.3 ($\{(\boldsymbol{C}H_3)_2C\}_2CH$), 24.3 (2x $(\boldsymbol{C}H_3)_2CH$), 24.6 ($(\boldsymbol{C}H_3)_2CH$), 24.7 ($(\boldsymbol{C}H_3)_2CH$), 24.9 ($(\boldsymbol{C}H_3)_2CH$), 25.0 (2x $(\boldsymbol{C}H_3)_2CH$), 27.0 ($(\boldsymbol{C}H_3)_2CH$), 28.1 (2x $(CH_3)_2\boldsymbol{C}H$), 28.1 (2x $(CH_3)_2\boldsymbol{C}H$), 29.1 (2x $(CH_3)_2\boldsymbol{C}H$), 29.6 (2x $(CH_3)_2\boldsymbol{C}H$), 84.9 ($\boldsymbol{C}_5H_5$), 98.6 ($\{(CH_3)_2C\}_2\boldsymbol{C}H$), 98.8 ($\{(CH_3)_2C\}_2\boldsymbol{C}H$), 124.0 (Ar***C***), 124.2 (Ar***C***), 125.0 (Ar***C***), 125.3 (Ar***C***), 127.6 (Ar***C***), 127.7 (Ar***C***), 137.3 ($\boldsymbol{C}^2$), 138.6 (Ar***C***), 139.2 (Ar***C***), 143.2 (Ar***C***), 143.6 (Ar***C***), 145.6 (Ar***C***), 145.9 (Ar***C***), 163.5 ($\boldsymbol{C}^3$), 172.0 ($\{(CH_3)_2\boldsymbol{C}\}_2CH$), 172.4 ($\{(CH_3)_2\boldsymbol{C}\}_2CH$).

Some Ar***C*** resonances are overlapping and cannot be observed. The Al–$\boldsymbol{C}^3$ and Co–$\boldsymbol{C}^1$ resonance could not be observed in the ^{13}C NMR spectrum due to coupling to the quadrupolar ^{27}Al ($I = 5/2$) and ^{59}Co ($I = 7/2$) nuclei respectively.

IR (ATR), ν_{CO} (cm^{-1}): 1941 (s), 1569 (m).

2.1.1 – Key ^{13}C NMR spectroscopic data of the carbon chain

	**3-Cr**	**3-Mo**	**3-W**	**3-Mn**	**3-Re**	**3-Re'**	**3-Co**
C^1 (ppm)	314.6	305.7	288.6	311.7	265.0	273.0	-
C^2 (ppm)	169.2	170.6	172.4	166.7	170.5	178.5	-
C^3 (ppm)	-	-	176.5[a]	-	-	-	-

Table S1: ^{13}C key resonances for complexes **3-M**. a - Measured from a ^{13}C labelled sample.

	**4-Cr**	**4-Mo**[a]	**4-W**[b]	**4-Mn**	**4-Re**	**4-Co**
C^1 (ppm)	348.4	348.8	329.2	332.3	280.6	-
C^2 (ppm)	160.8	161.7	164.6	160.7	169.1	163.5
C^3 (ppm)	140.1	144.7	135.3	135.7	144.5	137.3
C^4 (ppm)	-	-	260.6	-	-	-

Table S2: ^{13}C key resonances for complexes **4-M**. a - Spectra acquired at 353K. b -Measured from a ^{13}C labelled sample.

2.1.2 – Synthesis of a ^{13}C labelled sample of 3-W

The preparation of a ^{13}C labelled sample of 3-W was performed using the literature procedure[3] using $[W(^{13}CO)_6]$ and ^{13}CO gas.

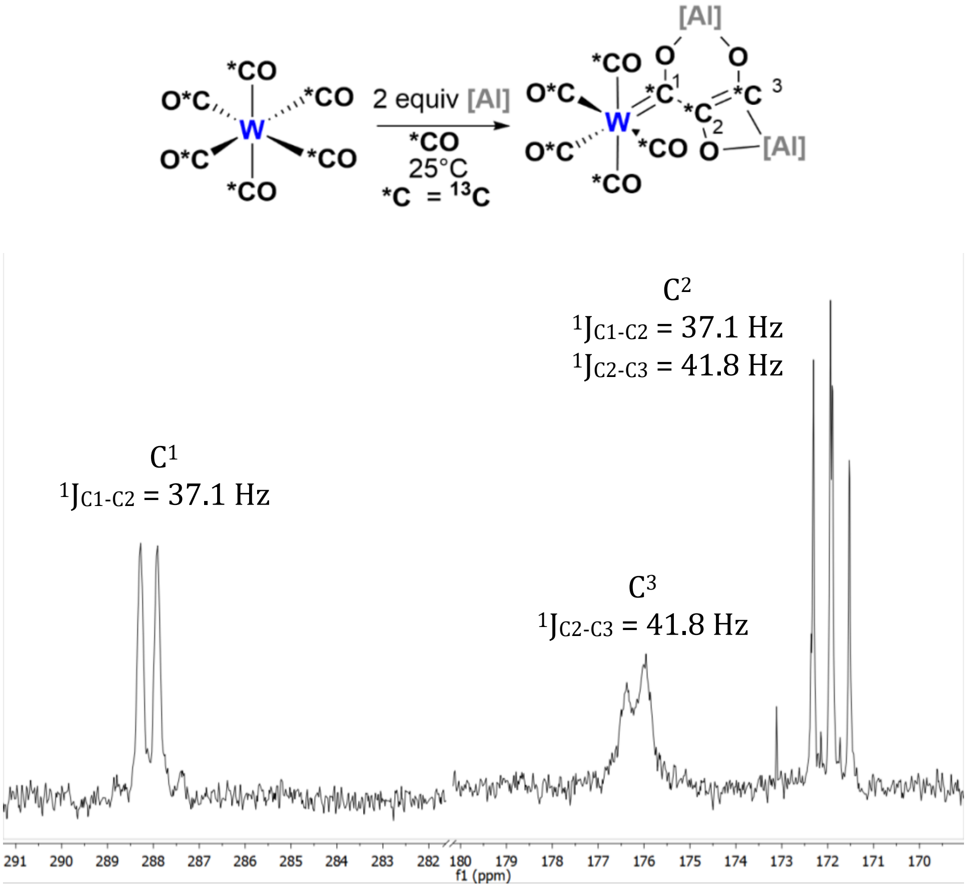

Figure 2.1: ^{13}C NMR spectrum of C^1, C^3, and C^3 ^{13}C labelled **2-3-W**.

2.2 – VT NMR of 2-Mn

In a glovebox, **2-Mn** (6.7 mg, 0.0059 mmol) was dissolved in toluene-*d*$_8$ (0.55 mL) and transferred to a J-Young NMR tube. The sample was then placed into a pre-cooled spectrometer (−67 °C). The spectrometer was warmed to −60 °C and subsequently gradually warmed by 10 °C intervals until 80 °C. ^{1}H NMR spectra were taken at each temperature.

Fluxionality is clear at low and high temperatures. A broad isopropyl methyl environment which resonates at δ = 0.49 ppm at 25 °C (*vide supra*) resolve to two doublets at lower temperatures. Similar resolution is observed for the isopropyl methine resonances, and broad manganese methylcyclopentadienyl resonances. The data suggest that one of the aluminium β-diketiminate environments is fluxional.

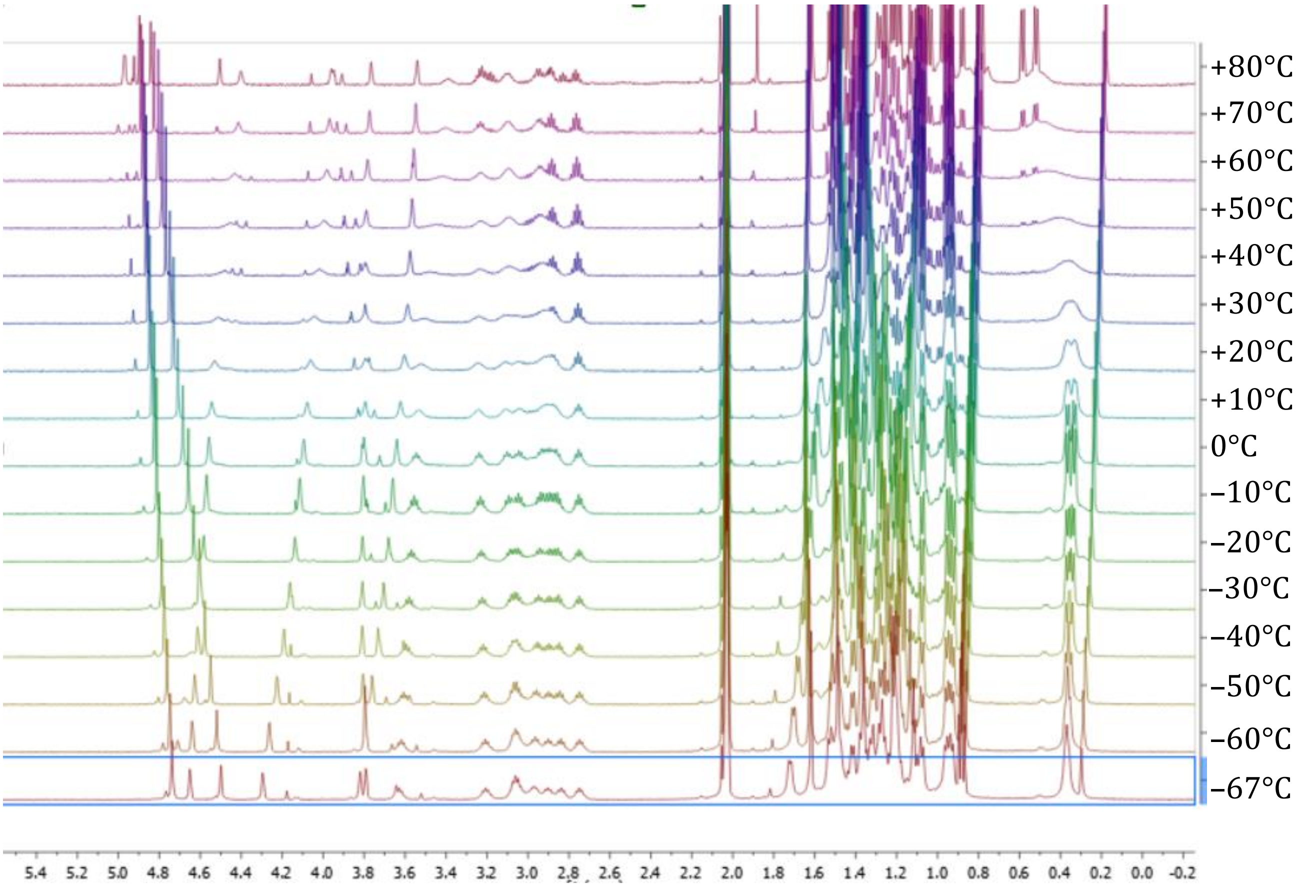

Figure S1: Variable-temperature ^{1}H NMR spectra of **2-Mn**.

3 X-Ray Data

The X-ray structure of **2-Mn**

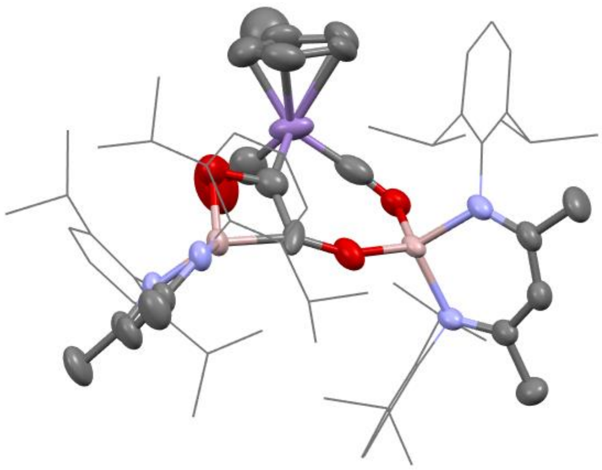

Figure S2: The X-ray structure of **2-Mn**. All hydrogen atoms are omitted for clarity.

2-Mn was found to crystallise in the $P2_1/n$ space group with one included hexane molecule in the asymmetric unit for a total of four within the unit cell.

The methylcyclopentadienyl ligand on manganese was found to be disordered. The carbon atoms C5>C10 were modelled as disordered over two sites in *ca.* 55:45 occupancies for the major and minor orientations respectively. The thermal parameters of adjacent atoms in the major and minor components were restrained to be similar, and only the non-hydrogen atoms in the major orientation were refined anisotropically (those in the minor orientation were refined isotropically).

The included hexane molecule was found to be disordered. The carbon atoms C69>C74 were modelled as disordered over two sites in *ca.* 74:26 occupancies for the major and minor orientations respectively. The thermal parameters of adjacent atoms in the major and minor components were restrained to be similar, their geometries optimized, and only the non-hydrogen atoms in the major orientation were refined anisotropically (those in the minor orientation were refined isotropically).

Crystal Data for $C_{74}H_{103}Al_2MnN_4O_4$, *M* =1221.50, monoclinic, space group $P2_1/n$ (no. 14), *a* = 12.8408(5) Å, *b* = 27.5925(9) Å, *c* = 19.8503(6) Å, *β* = 92.256(3)°, *V* = 7027.7(4) Å^3, *Z* = 4, ρ_{calc}g/cm^3 = 1.154, μ(MoKα) = 0.262 mm^{-1}, *T* = 173.00(14), black blocks, F^2 refinement, R_1(obs) = 0.0843, wR_2(all) = 0.2668, 14077 independent observed reflections (R_{int} = 0.0309), 8735 independent measured reflections [$|F_o| > 4\sigma(|F_o|)$, $2\theta_{full}$ = 56.704], 820 parameters. CCDC 2095244.

The X-ray structure of **3-Mn**

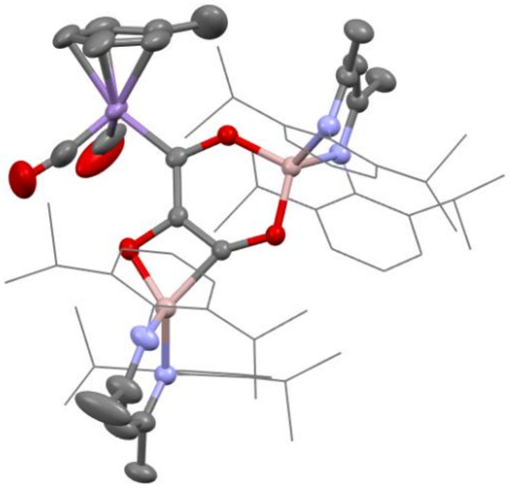

Figure S3: The X-ray structure of **3-Mn**. All hydrogen atoms are omitted for clarity.

3-Mn was found to crystallise in the $P2_1/n$ space group with one included toluene molecule for a total of four within the unit cell.

The included benzene molecule (C70>C76) was found to be disordered. As a result, the molecule was modelled as disordered over two sites in *ca.* 63:37 occupancies for the major and minor orientations respectively. The thermal parameters of adjacent atoms in the major and minor components were restrained to be similar, and only the non-hydrogen atoms in the major orientation were refined anisotropically (those in the minor orientation were refined isotropically).

The iso-propyl group C52>C54 was found to be disordered. C53 and C54 were modelled as disordered over two sites in *ca.* 81:19 occupancies for the major and minor orientations respectively. The thermal parameters of adjacent atoms in the major and minor components were restrained to be similar, and only the non-hydrogen atoms in the major orientation were refined anisotropically (those in the minor orientation were refined isotropically).

Crystal Data for $C_{76}H_{96}Al_2MnN_4O_5$, *M* =1254.46, monoclinic, space group $P2_1/n$ (no. 14), *a* = 16.8912(5) Å, *b* = 20.7695(8) Å, *c* = 19.8211(8) Å, *β* = 90.526(3)°, *V* = 6953.4(4) Å^3, *Z* = 4, ρ_{calc}g/cm^3 = 1.198, μ(MoKα) = 0.268 mm^{-1}, *T* = 173.00(14), red blocks, F^2 refinement, R_1(obs) = 0.0765, wR_2(all) = 0.2194, 14003 independent observed reflections (R_{int} = 0.0484), 9373 independent measured reflections [$|F_o| > 4\sigma(|F_o|)$, $2\theta_{full}$ = 56.614], 844 parameters. CCDC 2095245.

The X-ray structure of **4-Mn**

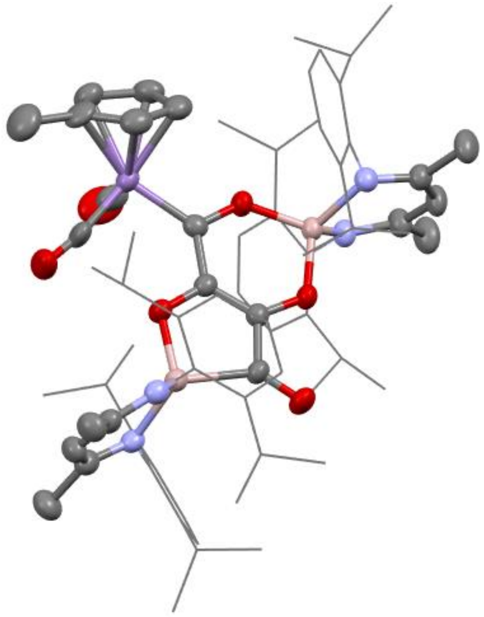

Figure S4: The X-ray crystal structure of **4-Mn**. All hydrogen atoms are omitted for clarity.

4-Mn was found to crystallise in the $P2_1/n$ space group with two included toluene molecules in the asymmetric unit, for a total of eight toluene molecules in the unit cell. The crystal was modelled as a two-component twin in ca. 75:25 ratio. The two twin lattices are related by the approximate twin law [-1.0 0.0 0.0 0.0 -1.0 0.0 0.0 0.0 1.0].

The included toluene (C70>C77) was found to be disordered. As a result the molecule was modelled as disordered over two sites in *ca.* 65:35 occupancies for the major and minor orientations respectively. The thermal parameters of adjacent atoms in the major and minor components were restrained to be similar, and only the non-hydrogen atoms in the major orientation were refined anisotropically (those in the minor orientation were refined isotropically).

The included toluene (C78>C83) was found to be disordered. As a result the molecule was modelled as disordered over two sites in *ca.* 58:42 occupancies for the major and minor orientations respectively. The thermal parameters of adjacent atoms in the major and minor components were restrained to be similar, and only the non-hydrogen atoms in the major orientation were refined anisotropically (those in the minor orientation were refined isotropically).

Crystal Data for $C_{84}H_{105}Al_2MnN_4O_6$, *M* =1375.61, monoclinic, space group $P2_1/n$ (no. 14), *a* = 16.8869(7) Å, *b* = 22.7289(9) Å, *c* = 19.8568(9) Å, *β* = 91.256(4)°, *V* = 7619.7(6) Å^3, *Z* = 4, ρ_{calc}g/cm^3 = 1.199, μ(CuKα) = 2.057 mm^{-1}, *T* = 173.1(2), red blocks, F^2 refinement, R_1(obs) = 0.0752, wR_2(all) = 0.2248, 24570 independent observed reflections (R_{int} = 0.0737), 11769 independent measured reflections [$|F_o| > 4\sigma(|F_o|)$, $2\theta_{full}$ = 147.336], 909 parameters. CCDC 2095246.

The X-ray structure of **3-Re**

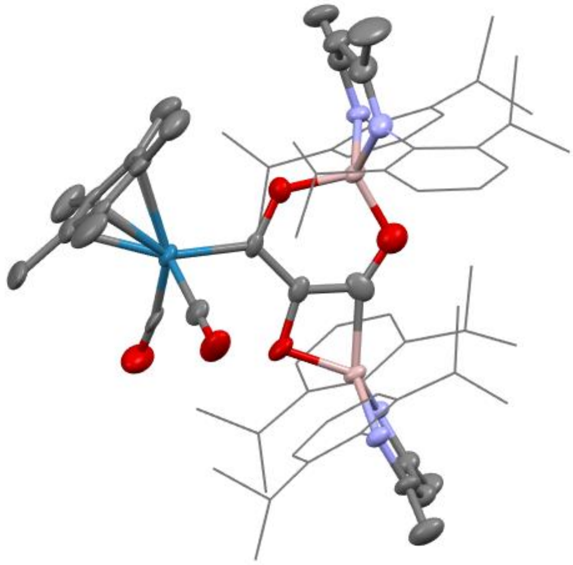

Figure S5: The solid-state structure of **3-Re**. All hydrogen atoms are omitted for clarity.

3-Re was found to crystallise in the Pbca space group. **3-Re** is isomorphous to **3-Re'** (*vide infra*) and as a result, both **3-Re** and **3-Re'** co-crystallise from the solution. ^{1}H NMR spectroscopy of the single crystals suggests that the **3-Re** and **3-Re'** compounds are present in the measured crystal in a ca. 75:25 ratio. As a result, disorder of the two isomeric forms of the C1>C3 carbon chain (in **3-Re** and **3-Re'** respectively) were present. The high degree of overlap (Figure S6) between the two isomeric fragments precluded a reasonable model of the minor-component of the disorder. A single Q-peak ca. 1.0 Å away from C3 and corresponding to 2.0 e^{-} A^{-3} was observed and is consistent with a ca. 30% occupancy oxygen atom.

The iso-propyl group C30>C32 was found to be disordered. C31 and C32 were modelled as disordered over two sites in *ca.* 69:31 occupancies for the major and minor orientations respectively. The thermal parameters of adjacent atoms in the major and minor components were restrained to be similar, and their geometries were optimized.

The iso-propyl group C59>C61 was found to be disordered. C59>C61 were modelled as disordered over two sites in *ca.* 57:43 occupancies for the major and minor orientations respectively. The thermal parameters of adjacent atoms in the major and minor components were restrained to be similar, their geometries were optimized, and only the

non-hydrogen atoms in the major orientation were refined anisotropically (those in the minor orientation were refined isotropically).

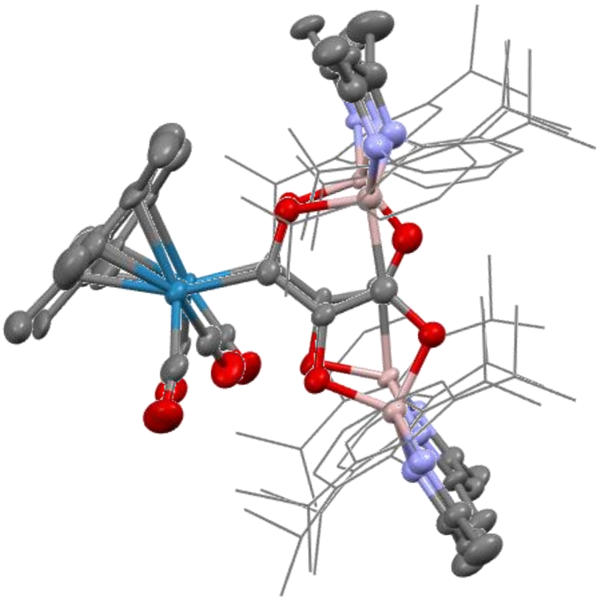

Figure S6: Overlay of **3-Re'** (front) and **3-Re** (behind). All hydrogen atoms are omitted for clarity.

3-Re crystallises with 1.5 pentane equivalents in the asymmetric unit, for a total of 12 pentane molecules within the unit cell. The included pentane molecule spanning carbon atoms C79>C83 was found to be in 50% occupancy and disordered over two positions in *ca*. 26:24 ratio for the major and minor orientations respectively. The thermal parameters of adjacent atoms in the major and minor components were restrained to be similar, their geometries were optimized, and only the non-hydrogen atoms in the major orientation were refined anisotropically (those in the minor orientation were refined isotropically).

The included pentane molecule spanning carbon atoms C74>C78 was found to be disordered over three positions in *ca.* 40:34:26 ratio for the three orientations. The thermal parameters of the entire disordered pentane component were restrained to be similar, their geometries were optimized, and all atoms were refined isotropically.

Crystal Data for $C_{80.5}H_{115}Al_2N_4O_5Re$, M =1458.92, orthorhombic, space group Pbca (no. 61), a = 45.706(2) Å, b = 17.2177(17) Å, c = 20.3938(17) Å, V = 16049(2) Å^3, Z = 8, ρ_{calc}g/cm^3 = 1.208, μ(MoKα) = 1.585 mm^{-1}, T = 173.00(14), red plates, F^2 refinement, R_1(obs) = 0.0766, wR_2(all) = 0.1662, 16110 independent observed reflections (R_{int} =

0.0589), 11818 independent measured reflections [$|F_o| > 4\sigma(|F_o|)$, $2\theta_{full} = 56.75$], 956 parameters. CCDC 2095247.

The X-ray structure of **3-Re'**

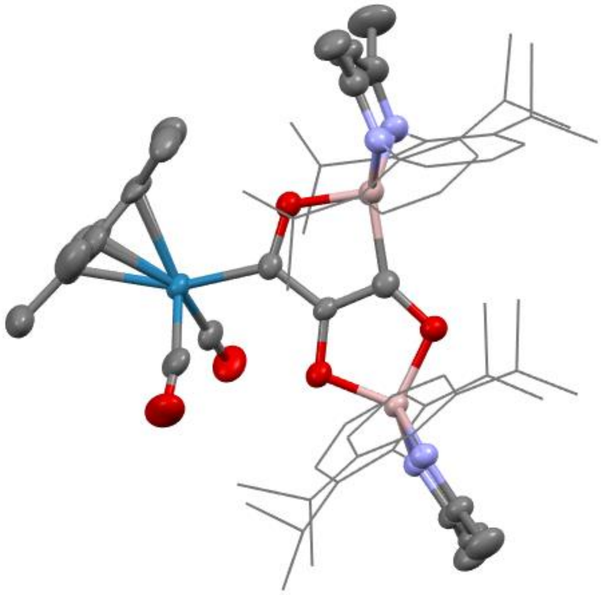

Figure S7: The X-ray structure of **3-Re'**. All hydrogen atoms are omitted for clarity.

3-Re' was found to crystallise in the Pbca space group with 0.25 of a toluene molecule and 0.25 of a pentane molecule included in the asymmetric unit for a total of four toluene molecules and four pentane molecules within the unit cell. The included toluene molecule was (C74>C79) was fixed at 25% occupancy based on inspection of thermal parameters at various occupancies.

The included pentane (C81>C85) molecule was fixed at 25% occupancy on the basis of inspection of thermal parameters at various occupancies. The geometry of the molecule was optimized, and the thermal parameters of carbon atoms were restrained to be similar.

The iso-propyl group C27>C29 was found to be disordered. C28 and C29 were modelled as disordered over two sites in *ca.* 56:44 occupancies for the major and minor orientations respectively. The thermal parameters of adjacent atoms in the major and minor components were restrained to be similar, their geometries were optimized, and only the non-hydrogen atoms in the major orientation were refined anisotropically (those in the minor orientation were refined isotropically).

The iso-propyl group C68>C70 was found to be disordered. C69 and C70 were modelled as disordered over two sites in *ca.* 58:42 occupancies for the major and minor orientations respectively. The thermal parameters of adjacent atoms in the major and minor components were restrained to be similar, their geometries were optimized, and only the non-hydrogen atoms in the major orientation were refined anisotropically (those in the minor orientation were refined isotropically).

Crystal Data for $C_{76}H_{102}Al_2N_4O_5Re$, M =1391.77, orthorhombic, space group Pbca (no. 61), a = 17.3541(4) Å, b = 20.3340(5) Å, c = 45.6601(19) Å, V = 16112.5(9) Å^3, Z = 8, ρ_{calc}g/cm^3 = 1.147, μ(CuKα) = 3.513 mm^{-1}, T = 173.00(14), yellow plates, F^2 refinement, R_1(obs) = 0.0535, wR_2(all) = 0.1669, 15643 independent observed reflections (R_{int} = 0.0447), 11650 independent measured reflections [$|F_o| > 4\sigma(|F_o|)$, $2\theta_{full}$ = 146.892], 849 parameters. CCDC 2095248.

The X-ray structure of **4-Re**

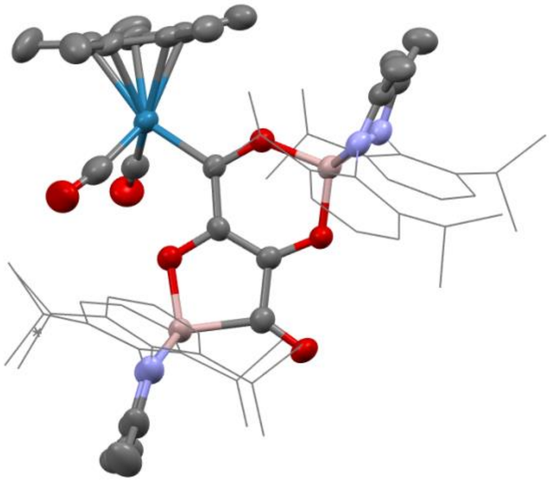

Figure S8: The X-ray crystal structure of **4-Re**. All hydrogen atoms are omitted for clarity.

4-Re was found to crystallise in the $P2_1/n$ space group with 1.5 benzene molecules in the asymmetric unit for a total of six within the unit cell.

The included benzene molecule (C80, C81, C83, C84, C86, C89) was modelled as disordered over two sites in *ca.* 75:25 occupancies for the major and minor orientations respectively. The thermal parameters of adjacent atoms in the major and minor components were restrained to be similar, and only the non-hydrogen atoms in the major orientation were refined anisotropically (those in the minor orientation were refined isotropically).

The included benzene molecule (C90, C92, C93, C96, C97, C100) was found to be disordered over a special position in two separate orientations. As a result, the molecule was modelled as disordered over two sites in *ca.* 37:13 occupancies for the major and minor orientations respectively, to generate one 50% occupancy molecule of benzene (with the symmetry element generating the other 50%.). The thermal parameters of adjacent atoms in the major and minor components were restrained to be similar, and only the non-hydrogen atoms in the major orientation were refined anisotropically (those in the minor orientation were refined isotropically).

The iso-propyl group C55>C57 was found to be disordered. C55>C57 were modelled as disordered over two sites in *ca.* 75:25 occupancies for the major and minor orientations respectively. The thermal parameters of adjacent atoms in the major and minor

components were restrained to be similar, and only the non-hydrogen atoms in the major orientation were refined anisotropically (those in the minor orientation were refined isotropically).

The iso-propyl group C70>C72 was found to be disordered. C70>C72 were modelled as disordered over two sites in *ca.* 62:38 occupancies for the major and minor orientations respectively. The thermal parameters of adjacent atoms in the major and minor components were restrained to be similar, and only the non-hydrogen atoms in the major orientation were refined anisotropically (those in the minor orientation were refined isotropically).

Crystal Data for $C_{83}H_{106}Al_2N_4O_6Re$, M =1495.87, monoclinic, space group $P2_1/n$ (no. 14), a = 13.5551(2) Å, b = 15.5294(3) Å, c = 36.6963(10) Å, β = 95.819(2)°, V = 7684.9(3) Å^3, Z = 4, ρ_{calc}g/cm^3 = 1.293, μ(CuKα) = 3.731 mm^{-1}, T = 173.1(2), red plates, F^2 refinement, R_1(obs) = 0.0491, wR_2(all) = 0.1287, 14739 independent observed reflections (R_{int} = 0.0461), 10643 independent measured reflections [$|F_o| > 4\sigma(|F_o|)$, $2\theta_{full}$ = 146.804], 918 parameters. CCDC 2095249.

The X-ray structure of **3-Cr**

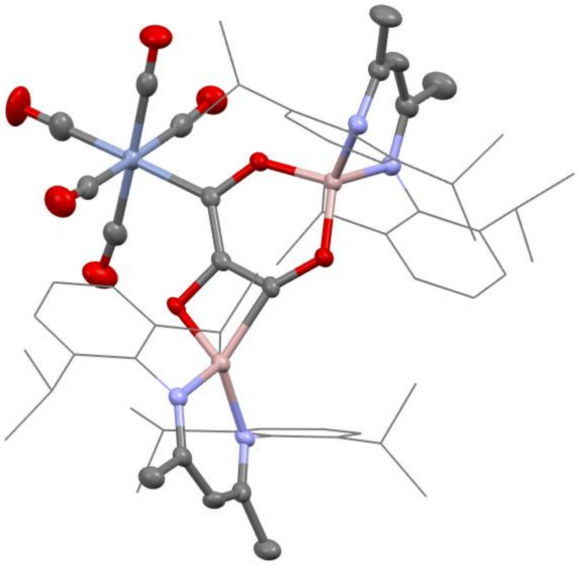

Figure S9: The X-ray crystal structure of **3-Cr**. All hydrogen atoms are omitted for clarity.

3-Cr was found to crystallise in the $P2_1/c$ space group, with 3.5 molecules of benzene in the asymmetric unit for a total of 14 benzene molecules in the unit cell.

The iso-propyl group C20>C22 was found to be disordered. C21 and C22 were modelled as disordered over two sites in *ca.* 69:31 occupancies for the major and minor orientations respectively. The thermal parameters of adjacent atoms in the major and minor components were restrained to be similar, and only the non-hydrogen atoms in the major orientation were refined anisotropically (those in the minor orientation were refined isotropically).

The included benzene molecule (C66>C71) was found to be disordered. As a result, the molecule was modelled as disordered over two sites in *ca.* 80:20 occupancies for the major and minor orientations respectively. The thermal parameters of adjacent atoms in the major and minor components were restrained to be similar, their geometries optimised and only the non-hydrogen atoms in the major orientation were refined anisotropically (those in the minor orientation were refined isotropically).

The included benzene molecule (C73>C78) was found to be disordered. As a result, the molecule was modelled as disordered over two sites in *ca.* 75:25 occupancies for the

major and minor orientations respectively. The thermal parameters of adjacent atoms in the major and minor components were restrained to be similar, their geometries optimised and only the non-hydrogen atoms in the major orientation were refined anisotropically (those in the minor orientation were refined isotropically).

The included benzene molecule (C73>C78) was found to be disordered over a special position in two different orientations. As a result, the molecule was modelled as disordered over two sites in *ca.* 31:19 occupancies for the major and minor orientations respectively to yield one 50% occupancy molecule (with the symmetry element generating the other 50%). The thermal parameters of adjacent atoms in the major and minor components were restrained to be similar, their geometries optimised and only the non-hydrogen atoms in the major orientation were refined anisotropically (those in the minor orientation were refined isotropically).

Crystal Data for $C_{87}H_{100}Al_2CrN_4O_8$, M =1435.66, monoclinic, space group P2₁/c (no. 14), a = 27.4194(8) Å, b = 12.1333(4) Å, c = 24.9290(9) Å, β = 102.408(3)°, V = 8099.9(5) Å^3, Z = 4, ρ_{calc}g/cm^3 = 1.177, μ(MoKα) = 0.219 mm^{-1}, T = 173.00(14), orange plates, F^2 refinement, R_1(obs) = 0.0584, wR_2(all) = 0.1390, 16211 independent observed reflections (R_{int} = 0.0435), 9491 independent measured reflections [$|F_o| > 4\sigma(|F_o|)$, $2\theta_{full}$ = 56.598], 951 parameters. CCDC 2095250.

The X-ray structure of **4-Cr**

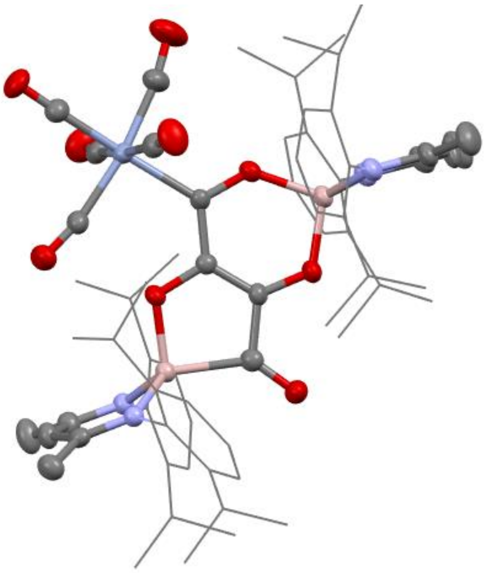

Figure S10: The X-ray crystal structure of **4-Cr**. All hydrogen atoms are omitted for clarity.

4-Cr was found to crystallise in the $P2_1/c$ space group, with one full toluene molecule in the asymmetric unit for a total of four toluene molecules in the unit cell.

Crystal Data for $C_{74}H_{90}Al_2CrN_4O_9$, M =1285.45, monoclinic, space group $P2_1/c$ (no. 14), a = 15.2826(5) Å, b = 21.3743(9) Å, c = 21.5531(8) Å, β = 92.741(3)°, V = 7032.4(5) Å^3, Z = 4, ρ_{calc}g/cm^3 = 1.214, μ(CuKα) = 2.041 mm^{-1}, T = 173.0(3), dull dark violet needles, F^2 refinement, R_1(obs) = 0.0635, wR_2(all) = 0.1480, 13489 independent observed reflections (R_{int} = 0.0806), 8280 independent measured reflections [$|F_o| > 4\sigma(|F_o|)$, $2\theta_{full}$ = 147.074], 832 parameters. CCDC 2095251.

The X-ray structure of **3-Mo**

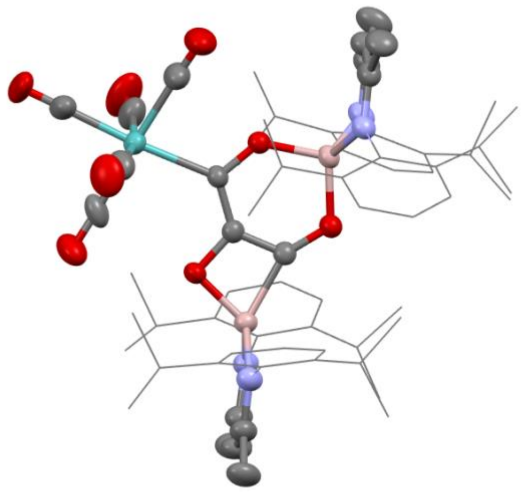

Figure S11: The X-ray crystal structure of **3-Mo**. One of two independent molecules shown for clarity. All hydrogen atoms are omitted for clarity.

3-Mo was found to crystallise in the $P2_1/c$ space group with two independent molecules, one quarter of an included toluene molecule, and one quarter of an included hexane molecule in the asymmetric unit for a total of eight whole molecules of **3-Mo**, one molecule of toluene and one molecule of hexane in the unit cell. The crystal was modelled as a two-component twin in ca. 63:37 ratio. The two twin lattices are related by the approximate twin law [-1.0 0.0 -0.0 -0.0 1.0 0.0 0.0 0.0 -1.0].

The included toluene (C73>C80) was found to be approximately 0.25 occupancy by inspection of thermal ellipsoids. The thermal parameters of the atoms were restrained to be similar, and the molecule was refined isotropically.

The included hexane (C67>C72) was found to be approximately 0.25 occupancy by inspection of thermal ellipsoids. The thermal parameters of the atoms were restrained to be similar, and the molecule was refined isotropically.

Crystal Data for $C_{67.625}H_{84.75}Al_2MoN_4O_8$, M =1231.54, monoclinic, space group $P2_1/c$ (no. 14), a = 28.5349(5) Å, b = 20.7875(3) Å, c = 26.1992(5) Å, β = 112.641(2)°, V = 14342.9(4) Å^3, Z = 8, ρ_{calc}g/cm^3 = 1.141, μ(CuKα) = 2.136 mm^{-1}, T = 173.0(3), orange blocks, F^2 refinement, R_1(obs) = 0.0702, wR_2(all) = 0.2193, 27591 independent observed reflections (R_{int} = 0.0509), 16366 independent measured reflections [$|F_o| > 4\sigma(|F_o|)$, $2\theta_{full}$ = 147.312], 1540 parameters. CCDC 2095252.

The X-ray structure of **4-Mo**

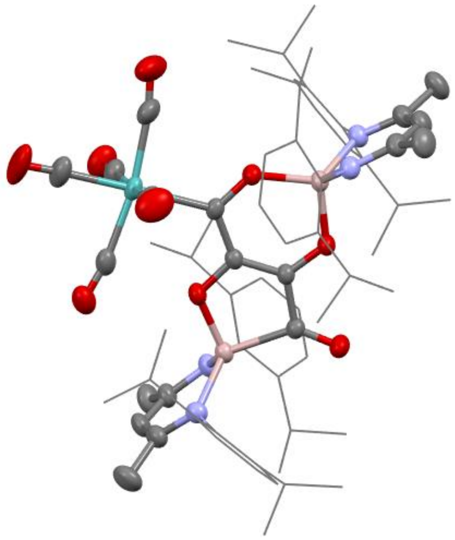

Figure S12: The X-ray crystal structure of **4-Mo**. All hydrogen atoms are omitted for clarity.

4-Mo was found to crystallise in the $P2_1/n$ space group, with one full toluene molecule in the asymmetric unit for a total of four toluene molecules in the unit cell.

Crystal Data for $C_{74}H_{90}Al_2MoN_4O_9$, M =1329.39, monoclinic, space group $P2_1/n$ (no. 14), a = 15.5122(5) Å, b = 12.6927(3) Å, c = 36.0314(9) Å, β = 96.194(3)°, V = 7052.9(3) Å^3, Z = 4, ρ_{calc}g/cm^3 = 1.252, μ(CuKα) = 2.222 mm^{-1}, T = 173.05(10), clear reddish red blocks, F^2 refinement, R_1(obs) = 0.0440, wR_2(all) = 0.1248, 13474 independent observed reflections (R_{int} = 0.0351), 10739 independent measured reflections [$|F_o| > 4\sigma(|F_o|)$, $2\theta_{full}$ = 147.13], 832 parameters. CCDC 2095253.

The X-ray structure of **3-Co**

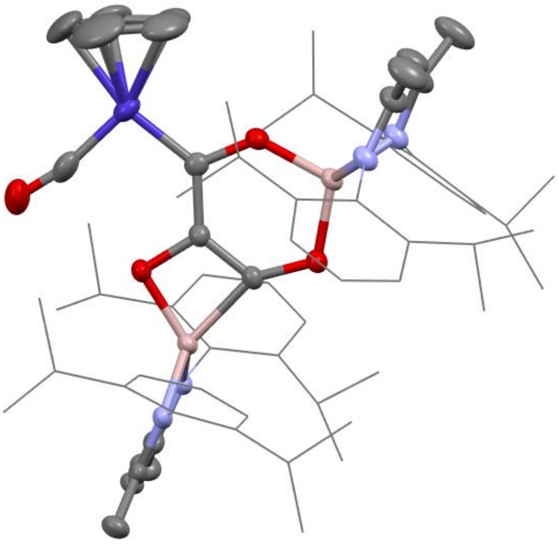

Figure S13: The X-ray crystal structure of **3-Co**. All hydrogen atoms are omitted for clarity.

3-Co was found to crystallise in the $P2_1/n$ space group, with 1.5 toluene molecules and 0.5 pentane molecules in the asymmetric unit for a total of six toluene molecules and two pentane molecules in the unit cell.

The included toluene molecule (C75>C81) was found to be disordered over a special position in two different orientations. As a result, the molecule was modelled as disordered over two sites in *ca.* 41:9 occupancies for the major and minor orientations respectively to yield one 50% occupancy molecule (with the symmetry element generating the other 50%). The thermal parameters of adjacent atoms in the major and minor components were restrained to be similar, their geometries optimised and only the non-hydrogen atoms in the major orientation were refined anisotropically (those in the minor orientation were refined isotropically).

The included pentane molecule (C82>C86) was found to be disordered over a special position in two different orientations. As a result the molecule was modelled as disordered over two sites in *ca.* 32:18 occupancies for the major and minor orientations respectively to yield one 50% occupancy molecule (with the symmetry element generating the other 50%). The thermal parameters of adjacent atoms in the major and minor components were restrained to be similar, their geometries optimised and only

the non-hydrogen atoms in the major orientation were refined anisotropically (those in the minor orientation were refined isotropically).

The iso-propyl group C21>C23 was found to be disordered. C22 and C23 were modelled as disordered over two sites in *ca.* 71:29 occupancies for the major and minor orientations respectively. The thermal parameters of adjacent atoms in the major and minor components were restrained to be similar, and only the non-hydrogen atoms in the major orientation were refined anisotropically (those in the minor orientation were refined isotropically).

Crystal Data for $C_{80}H_{105}Al_2CoN_4O_4$, M =1299.56, monoclinic, space group $P2_1/n$ (no. 14), a = 13.0460(2) Å, b = 25.0267(5) Å, c = 22.6264(5) Å, β = 96.9581(19)°, V = 7333.1(3) Å^3, Z = 4, ρ_{calc}g/cm^3 = 1.177, μ(MoKα) = 0.309 mm^{-1}, T = 173.00(14), red blocks, F^2 refinement, R_1(obs) = 0.0527, wR_2(all) = 0.1386, 14683 independent observed reflections (R_{int} = 0.0263), 10344 independent measured reflections [$|F_o| > 4\sigma(|F_o|)$, $2\theta_{full}$ = 56.698], 893 parameters. CCDC 2095254.

3.1 Normalised M=C for 3

	**3-Cr**	**3-Mo**	**3-W**	**3-Mn**	**3-Re**	**3-Re'**	**3-Co**
**M=C (Å)**	2.055(3)	2.250(4)	2.195(3)	1.922(3)	2.019(6)	2.015(4)	1.810(2)
**FSR**	1.04	1.06	1.04	0.99	0.98	0.98	0.97

Table S3: Calculated formal shortness ratios (FSR) for **3-M** using the Pyykko definition for covalent radii.[4] FSR is defined as the quotient of measured bond length (M=C) divided by the sum of the covalent radii of the constituent atom (M and C).

4 Density functional theory calculations

4.1 – Computational methods

DFT calculations were performed using Gaussian 09 (Revision D.01) using an ultrafine integration grid (int=ultrafine).[5] A hybrid basis set in which Al and transition metal centres were described with Stuttgart SDDAll pseudopotential and associated basis sets and the 6-31G** basis set were used for all other atoms in all calculations.[6,7]

Geometry optimisations and frequency calculations were performed using the ωB97X functional.[8] Frequency analyses for all stationary points were performed using the enhanced criteria to confirm the nature of the structures as either minima (no imaginary frequency) or transition states (only one imaginary frequency). Functional testing was performed with the B3LYP,[9] M062X,[10] and B3PW91[11] in addition to the M06L[10,12] and ωB97X functional.

The electronic energies of the optimised geometries were calculated using the M06L functional with the same basis set with solvent corrections (PCM, benzene, ε = 2.2706) and an empirical dispersion correction (Grimme, D3: B3LYP, M062X, B3PW91, M06L' Grimme D2: ωB97X) with Becke-Johnson damping in two cases (B3LYP, B3PW91).[13–15] The Gibbs free energy correction from the frequency calculation was added to this electronic energy to generate Gibbs free energy values for the calculated stationary points.

Intrinsic reaction coordinate (IRC) calculations were used to connect transition states and minima located on the potential energy surface allowing a full energy profile (calculated at 298.15 K, 1 atm) of the reaction to be constructed.[16] Natural Bond Orbital analysis was carried out using NBO 6.0 with the ωB97x functional.[17] NICS(0) calculations were performed using the gauge-independent atomic orbital method in Gaussian 09 (Revision D.01) with the ωB97X functional.

ETS-NOCV calculations were performed in the Orca 4.2.1 suite[18] with the def2-tzvpp basis set,[19] RIJCOSX approximation,[20] and ωB97X functional. All calculations were performed with an ultrafine integration grid (grid6).

4.2 –Calculated stationary points

Manganese pathway to **3-Mn**

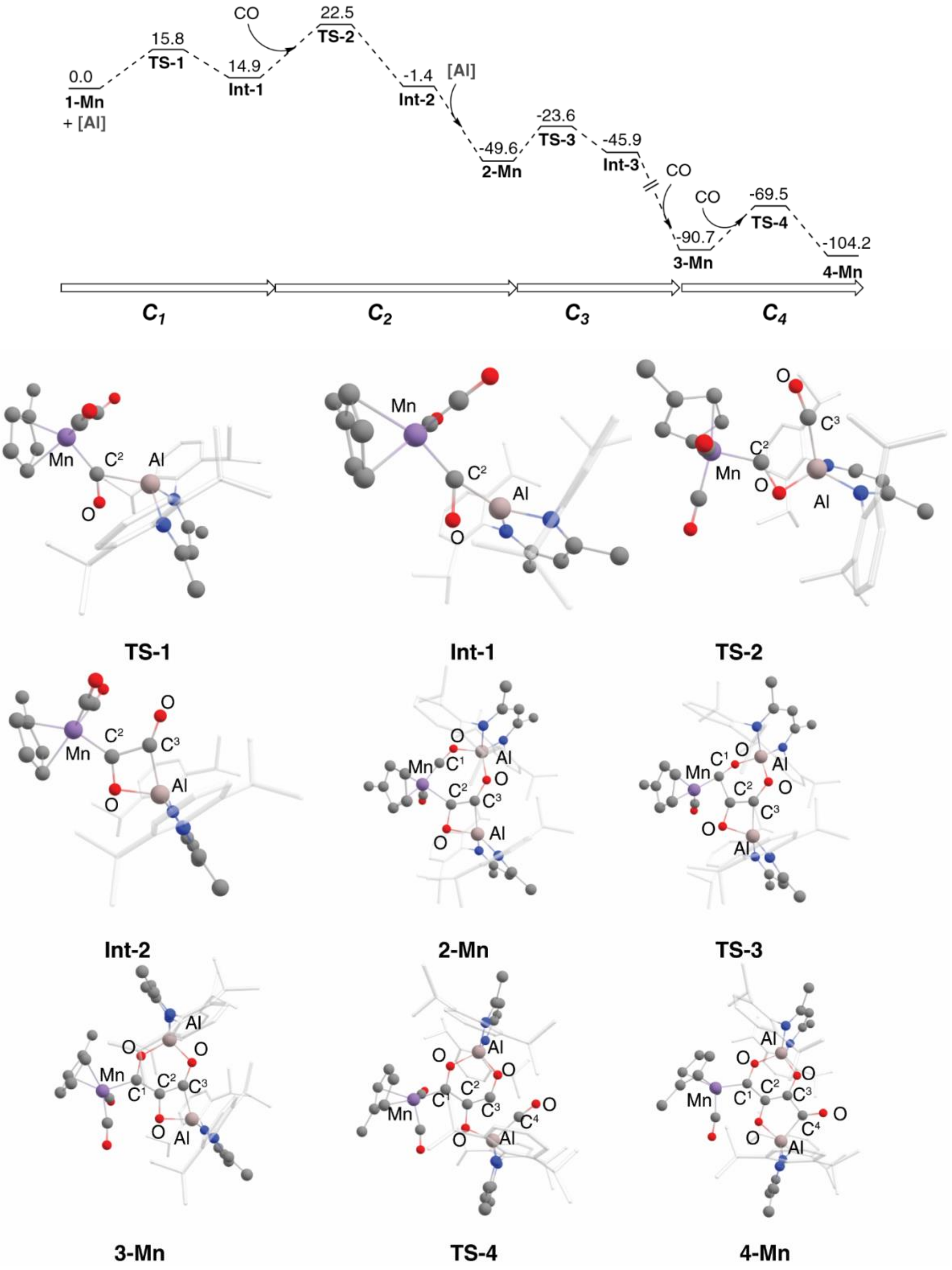

Figure S14: Calculated pathway to **4-Mn** from [(η^5-C_5H_4Me)Mn$(CO)_3$] (**1-Mn**), **[Al]**, and CO gas. All energies in kcal mol^{-1}.

Manganese pathway to **3-Mn’**

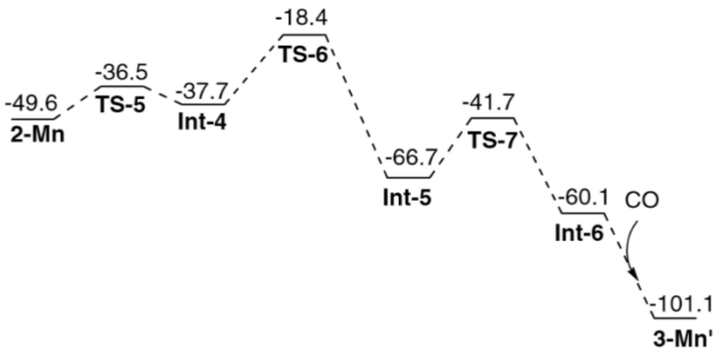

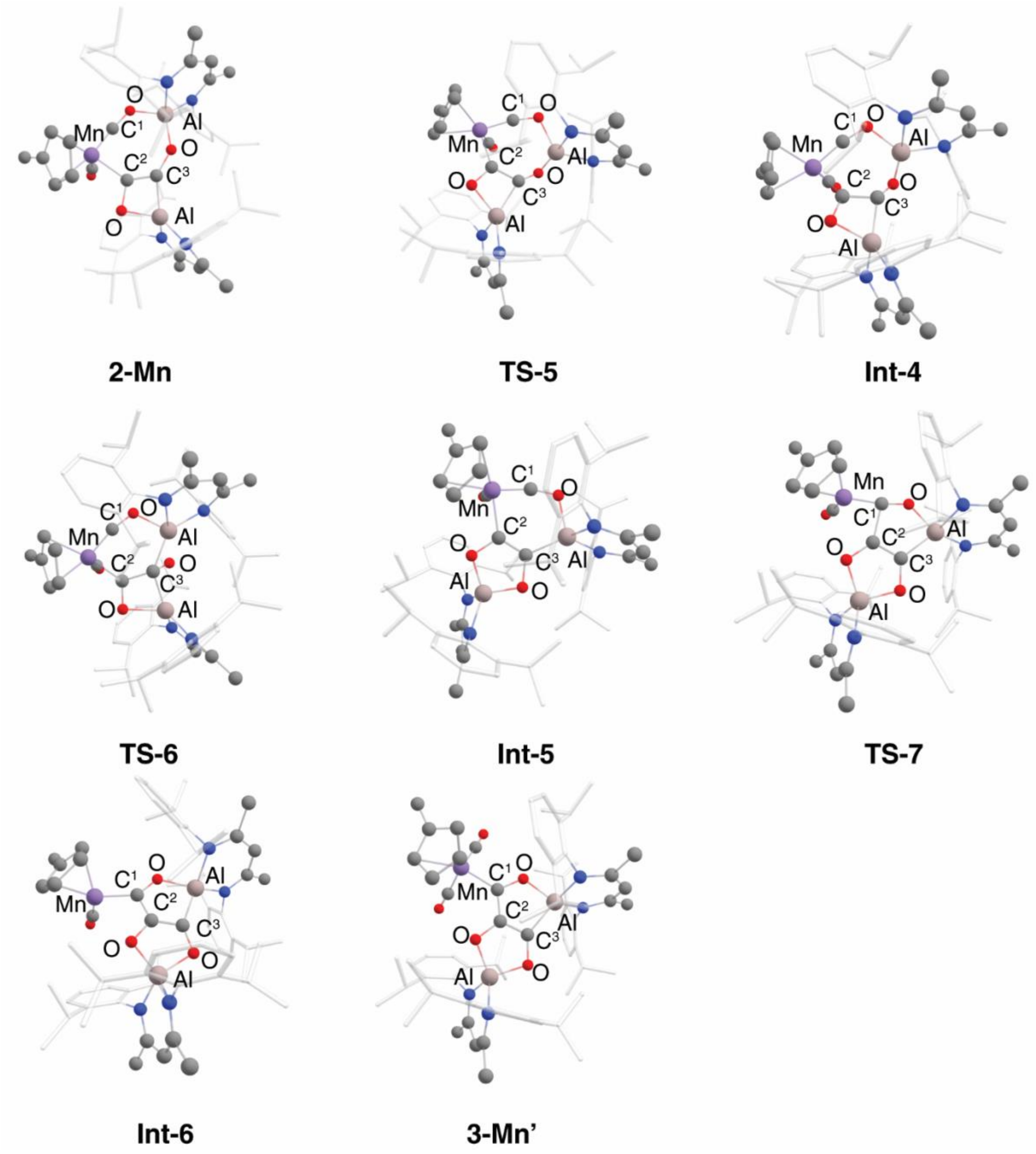

Figure S15: Calculated pathway to **3-Mn’** from **2-Mn**. All energies in kcal mol^{-1}.

*Tungsten pathway to **4-W***

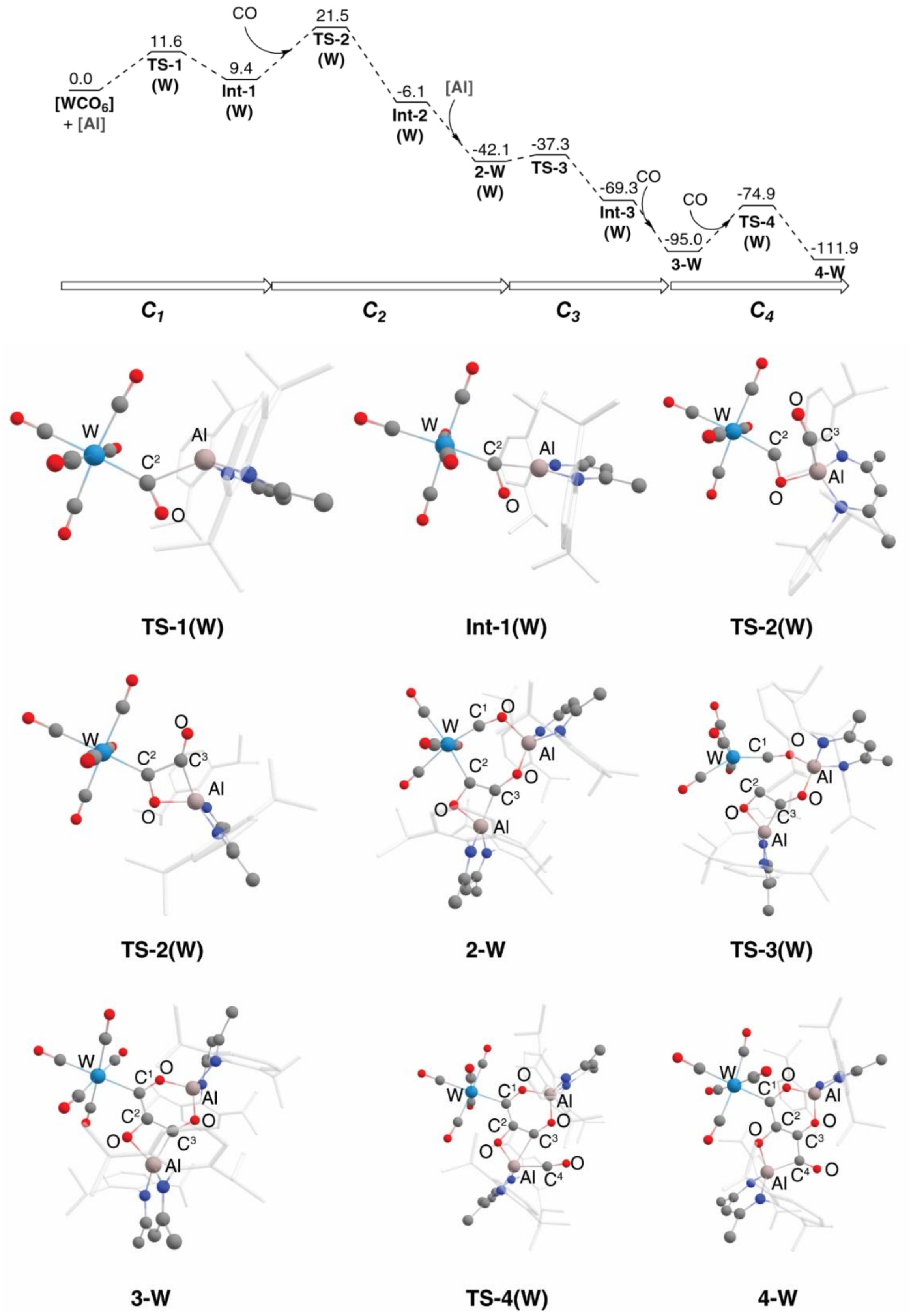

Figure S16: Calculated pathway to C_4 formation from $[W(CO)_6]$ and [Al].

The calculated pathway towards the formation of observed products **3-W** and **4-W** is similar to the calculated pathway for Mn. **TS-1(W)** and **TS-2(W)** have a similar activation barrier to the analogous transition states for the Mn pathway. The key difference in the W pathway to the Mn pathway is the activation energy to convert **2-W** to **3-W**: **TS-3(W)** is lower in energy ($\Delta G^{\ddagger}_{298K}$ = 4.8 kcal mol^{-1} from **2-W**) than the analogous activation barrier for Mn ($\Delta G^{\ddagger}_{298K}$ = 26.0 kcal mol^{-1} from **2-Mn**). The formation of **Int-3(W)** ($\Delta G°_{298K}$ = –27.2 kcal mol^{-1}) from **2-W** is also more exergonic than the analogous reaction for Mn ($\Delta G°_{298K}$ = +3.7 kcal mol^{-1} from **2-Mn**). The data are consistent with the formation of **3-W** in 5 minutes at 25 °C, compared to the slower reaction times required for the formation **3-Mn** and reflects the isolable nature of **2-Mn**.

4.3 – NBO data

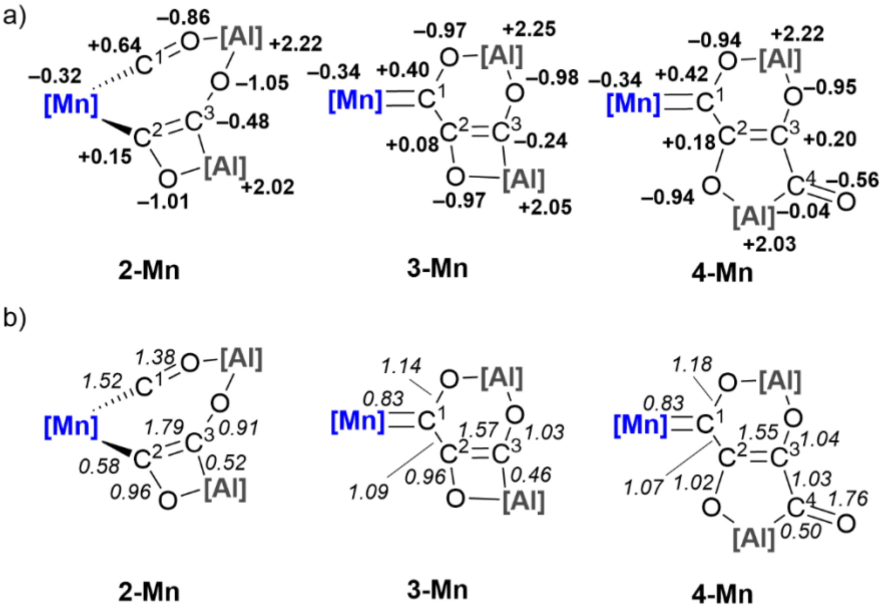

Figure S17: Calculated NBO data of **2-Mn**, **3-Mn**, and **4-Mn**. a) Natural charges provided in **bold** and b) Wiberg bond indices provided in *italics*.

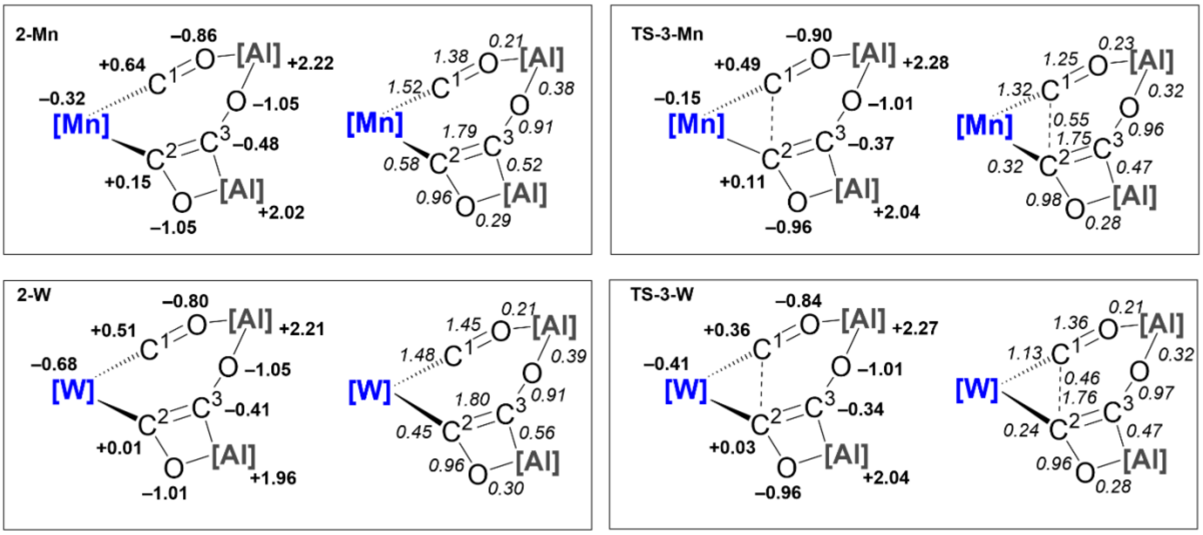

Figure S18: Comparison of calculated NBO data of **2-Mn** and **TS-3-Mn** with **2-W** and **TS-3-W**. Natural charges provided in **bold** and Wiberg bond indices provided in *italics*.

4.4 – NICS calculations on 3-Mn and 4-Mn

NICS(0) calculations were performed on the ring-systems involving the carbon chain in **3-Mn** and **4-Mn** respectively. The calculated values (Table S4) suggest that the ring systems are non-aromatic.

Ring Size	3-Mn	4-Mn
**6**	+1.95	+0.14
**4**	−6.20	–
**5**	–	+4.55

Table S4: Calculated NICS(0) values of the ring systems involving the carbon chain in **3-Mn** and **4-Mn**

4.5 – ETS-NOCV Calculations

2-Mn

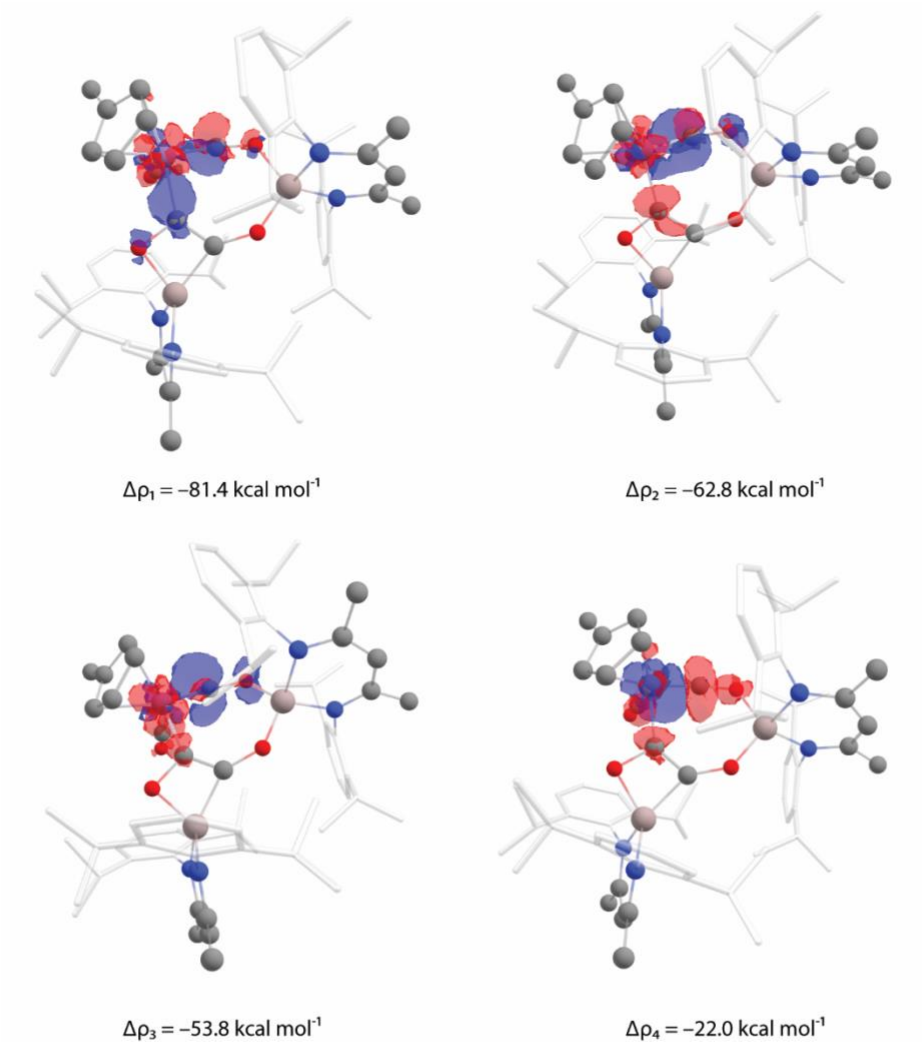

Figure S19: Deformation density plots for $\Delta\rho_{1\text{-}4}$ calculated for **2-Mn**. Charge flow from red to blue.

3-Mn

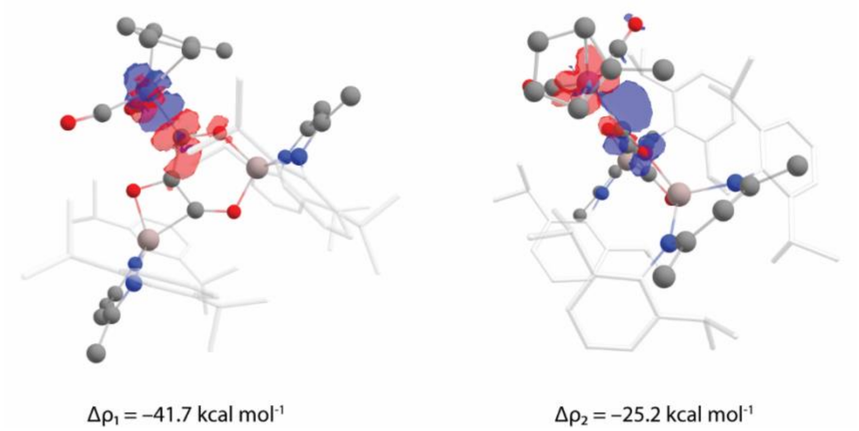

Figure S20: Deformation density plots for $\Delta\rho_{1\text{-}4}$ calculated for 3-**Mn**. Charge flow from red to blue.

4-Mn

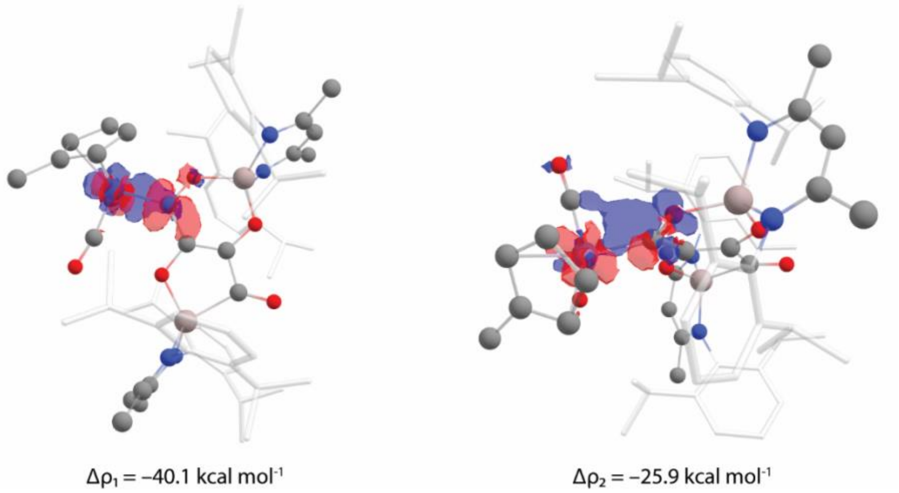

Figure S21: Deformation density plots for $\Delta\rho_{1\text{-}4}$ calculated for **2-Mn**. Charge flow from red to blue

4.6 – Functional testing on key stationary points

To investigate the formation of **3-Mn** and **3-Mn'**, functional testing was performed on key stationary points within the calculated pathway. The activation barriers of **TS-3** and **TS-6** were chosen to be compared.

Single point energy calculations on the geometries of **Int-3**, **TS-3** and **TS-6** which were optimised using the ωB97X functional were performed. Single point energies were calculated using the functionals B3LYP, B3PW91, M062X, ωB97XD, and M06L and solvent corrections (PCM, benzene) were applied in all cases. For the B3LYP and B3PW91 functionals, Grimme's D3 dispersion correction with Becke-Johnson damping was applied. In the case of M06L and M062X, the undamped D3 dispersion correction was applied. The free energy correction determined in the frequency calculation using the ωB97X functional (see Section 4.1) was added to the single point electronic energy to generate the Gibbs free energy.

A consistent trend is observed in which **TS-6** is higher in activation energy than **TS-3**. None of the functionals accurately reproduce the observed product distribution (91 : 9) which would imply an approximate $\Delta\Delta G^{\ddagger}$ = 1.4 kcal mol^{-1} between the two transition states. The M06L produces the smallest difference between these two activation energies ($\Delta\Delta G^{\ddagger}_{298K}$ = 5.3 kcal kcal mol^{-1}). M06L was found to have the best agreement with the experimental results: observation of the formation of **2-3-Mn'** in a 91:9 ratio imply a $\Delta\Delta G^{\ddagger}_{298K}$ = 1.4 kcal mol^{-1} for the two transition states.

Functional	TS-3	TS-6	ΔΔG
**M06L**	25.9	31.2	5.3
**M062X**	12.5	35.9	23.4
**B3LYP**	22.2	36.4	14.3
**B3PW91**	22.7	34.0	11.3
**ωB97xD**	20.3	35.3	15.0

Table S5: All Gibbs free energies provided in kcal mol^{-1}. Gibbs free energies for **TS-3** and **TS-6** provided relative to **Int-3**.

5 Computational coordinates

2-Mn.log

Lowest Frequency = 18.6680cm-1

Mn	3.236064	18.986447	4.254482
Al	-0.187948	18.755391	6.129572
Al	2.659698	14.957835	5.506820
O	3.600527	16.107119	4.486228
O	0.510268	17.221921	6.003945
N	-1.150554	18.954367	7.748339
O	1.143503	20.025386	5.967395
N	1.988540	13.237542	4.963410
N	3.918795	14.258986	6.779842
N	-1.531897	19.384930	4.951075
C	1.645798	16.593102	5.520668
C	-2.469450	19.169245	7.782820
C	4.994273	16.001730	8.168542
C	1.141811	13.182786	3.797078
C	-0.460731	18.783719	9.009518
C	-2.814086	19.495995	5.317954
C	-0.255496	13.097425	3.942212
C	-0.951722	13.198901	5.294313
H	-0.198142	13.076705	6.080182
C	5.058254	15.086923	7.107176
C	-1.192525	19.800269	3.605285
C	2.207593	12.106897	5.643660
C	-3.256181	19.355539	6.638663
H	-4.319426	19.474811	6.803605
C	-1.566392	14.596038	5.464742
H	-0.801782	15.380482	5.444070
H	-2.098270	14.663023	6.422879
H	-2.294732	14.792279	4.666712
C	3.886611	13.022141	7.266516
C	3.721614	16.243082	8.965102
H	2.973109	15.513424	8.637202
C	-1.066140	16.275477	9.011841
H	-1.582019	16.566500	8.090413
C	-0.849447	21.150155	3.390440
C	0.045498	15.293997	8.616353
H	0.704934	15.748179	7.871142
H	-0.387636	14.383991	8.183397
H	0.637001	14.999607	9.492333
C	-0.463566	17.525506	9.642815
C	3.023858	12.026777	6.778613
H	3.085883	11.058160	7.259236
C	1.743413	13.238652	2.522539
C	-1.033464	12.975843	2.788187
H	-2.114498	12.897996	2.880424
C	0.245559	21.254417	8.903362
H	-0.216883	21.165232	7.915512
C	-1.478131	17.389693	2.756616
H	-1.718163	17.223965	3.812911
C	6.220603	14.956956	6.322082
C	0.921175	13.117605	1.401260
H	1.362074	13.147204	0.409472
C	-0.453874	12.967180	1.528274
H	-1.075113	12.867699	0.641932
C	0.187188	19.892701	9.583310
C	-0.762814	22.170726	4.520143
H	-0.828094	21.633202	5.470546
C	-1.245166	18.878496	2.545475
C	-0.188395	16.616835	2.450736
H	0.106413	16.754623	1.402256
H	-0.337786	15.546447	2.618662
H	0.635228	16.941022	3.093931
C	6.136493	16.746708	8.469110
H	6.101881	17.464939	9.285001
C	-3.178589	19.227091	9.112198
H	-3.140295	18.255117	9.611410
H	-4.221907	19.514555	8.979998

H	-2.689711	19.942182	9.779662
C	1.570331	10.818051	5.180003
H	0.489990	10.835868	5.348243
H	1.990787	9.968288	5.719131
H	1.724304	10.673789	4.107043
C	3.927645	16.047014	10.471616
H	4.318336	15.050103	10.705844
H	2.975504	16.174269	10.999751
H	4.629550	16.782893	10.879843
C	2.687170	17.139824	4.824658
C	-0.612571	21.568487	2.079899
H	-0.355786	22.608295	1.891542
C	0.149009	17.420379	10.892648
H	0.153063	16.459182	11.401952
C	7.301792	16.605941	7.733000
H	8.179029	17.197901	7.980209
C	-1.931925	23.163257	4.466314
H	-1.936756	23.714228	3.518866
H	-1.850391	23.893659	5.278519
H	-2.899638	22.660436	4.566877
C	-2.022548	12.119945	5.498700
H	-2.879354	12.273089	4.833358
H	-2.400251	12.157009	6.526082
H	-1.639800	11.110255	5.314580
C	-1.015536	19.351984	1.251463
H	-1.058762	18.655473	0.417556
C	-2.083376	15.584887	9.929441
H	-2.550891	14.745186	9.403876
H	-2.880537	16.260442	10.257936
H	-1.603245	15.181102	10.827642
C	-0.712446	20.684855	1.013677
H	-0.535264	21.031754	-0.000795
C	-3.861455	19.805806	4.277688
H	-3.619945	20.719332	3.728802
H	-4.843879	19.913289	4.738348
H	-3.901539	18.998682	3.539849
C	7.333871	15.724433	6.660485
H	8.240684	15.636585	6.065760
C	3.166753	17.643011	8.668746
H	3.909012	18.415780	8.900761
H	2.274979	17.834869	9.272139
H	2.899235	17.729506	7.610481
C	0.575856	22.917974	4.514400
H	1.419584	22.222224	4.532813
H	0.647870	23.563275	5.397039
H	0.676001	23.559003	3.630937
C	6.297470	14.040402	5.106497
H	5.345208	13.506292	5.015948
C	4.852515	12.623369	8.354805
H	5.880020	12.637522	7.978693
H	4.626797	11.623027	8.726806
H	4.812507	13.336794	9.182916
C	3.250524	13.421843	2.368466
H	3.570607	14.140660	3.131252
C	-2.640747	16.843094	1.919930
H	-3.583666	17.364905	2.119036
H	-2.786866	15.779823	2.141221
H	-2.435927	16.926069	0.846729
C	0.762539	18.512405	11.492133
H	1.232702	18.406969	12.466301
C	1.694208	21.707178	8.686830
H	2.200997	21.896798	9.639911
H	1.716423	22.635954	8.106268
H	2.264504	20.952632	8.137797
C	0.791230	19.732064	10.832126
H	1.290850	20.580679	11.293494
C	2.071713	19.650444	5.217909
C	-0.546459	22.306717	9.689234
H	-1.599007	22.023378	9.800004
H	-0.510956	23.273407	9.175569
H	-0.130957	22.446567	10.693577
C	3.649852	14.040738	1.026818

H	3.516450	13.342737	0.191501
H	4.708362	14.318777	1.055514
H	3.071472	14.946035	0.814148
C	4.008937	12.105824	2.586430
H	3.860767	11.705106	3.594054
H	5.085642	12.260120	2.449159
H	3.682844	11.347652	1.863928
C	3.335555	18.085300	2.286429
H	3.028133	17.058207	2.140882
C	4.631010	18.492725	2.684663
H	5.478221	17.838655	2.849586
C	4.617391	19.913595	2.875412
C	3.301265	20.355555	2.599081
H	2.955718	21.380751	2.668168
C	2.503357	19.228568	2.236458
H	1.454414	19.249388	1.976653
C	6.482369	14.862033	3.823672
H	5.659411	15.577490	3.721013
H	6.498591	14.201161	2.947246
H	7.432801	15.409073	3.841522
C	7.399810	12.984062	5.249090
H	8.391295	13.446754	5.310210
H	7.399449	12.315324	4.380956
H	7.263795	12.369488	6.146032
C	5.795690	20.775178	3.224477
H	5.473064	21.699239	3.713338
H	6.358181	21.046826	2.323920
H	6.480224	20.256628	3.902445
C	4.450698	18.968575	5.556423
O	5.267914	19.023997	6.371284

3-Mn.log

Lowest Frequency = 18.3965cm-1

Mn	3.191717	16.076688	15.212659
Al	7.410033	17.762882	14.947053
Al	7.406912	13.171481	14.854887
O	5.709749	17.332334	15.100975
O	8.244818	16.239775	14.847691
O	5.684645	13.712692	14.956683
N	7.670353	18.917388	13.467060
N	7.904901	11.958725	13.477106
N	7.837173	19.007252	16.311690
N	8.310872	12.260541	16.265286
C	7.255060	20.176476	13.612788
C	7.764710	12.411694	12.110829
C	5.092149	16.145618	15.088860
C	9.488643	18.166102	20.118020
H	9.912816	17.979462	21.101525
C	6.602338	12.098054	11.383044
C	9.536423	18.824434	11.873277
C	7.404992	17.766325	11.291629
C	8.202953	18.496699	12.188920
C	8.401389	18.679089	17.605276
C	7.330629	20.237052	16.127790
C	8.804231	13.176723	11.547917
C	8.654762	13.626344	10.234632
H	9.443746	14.224912	9.784342
C	10.036813	18.456223	10.624969
H	11.064377	18.704134	10.369057
C	7.447614	15.140218	14.843678
C	8.420982	10.746545	13.702820
C	7.952848	17.422806	10.052106
H	7.344683	16.863901	9.345471
C	7.518370	13.323015	9.498972
H	7.419460	13.679159	8.476348
C	9.777123	18.914861	17.825118
C	9.249631	17.771093	9.709896
H	9.651522	17.499171	8.737105
C	8.684408	9.829218	12.535673
H	7.751254	9.598645	12.012699

H	9.142755	8.897989	12.869853
H	9.343334	10.311454	11.807766
C	6.503884	12.568896	10.072897
H	5.611364	12.344051	9.494410
O	2.770890	14.981084	12.536038
C	7.144096	21.147829	17.315054
H	6.381441	20.728612	17.979472
H	6.826241	22.141243	16.997072
H	8.063503	21.234008	17.898983
C	5.986034	17.316123	11.605626
H	5.765076	17.539907	12.653959
C	5.459328	11.285008	11.975976
H	5.812719	10.831997	12.909108
C	7.586111	18.143872	18.617323
C	6.963835	20.741176	14.870187
H	6.537535	21.736527	14.859897
C	5.948270	16.261237	18.387879
H	6.626399	15.773259	17.678529
H	4.924523	15.996940	18.100941
H	6.155169	15.841671	19.379576
C	9.553973	13.958834	17.531001
C	8.520197	12.997854	17.491863
C	8.153939	17.897113	19.869666
H	7.530998	17.482295	20.658589
C	7.135686	21.072018	12.407593
H	8.130992	21.391454	12.080689
H	6.550838	21.961118	12.647012
H	6.676335	20.544394	11.568282
C	10.291835	18.659419	19.096743
H	11.343062	18.840858	19.296080
O	2.874366	13.442757	16.456852
C	6.127551	17.784906	18.391126
H	5.841125	18.146068	17.400487
C	8.765406	10.277457	14.976590
H	9.153741	9.268067	15.029350
C	5.849605	15.802411	11.408525
H	5.968017	15.526110	10.355702
H	4.862959	15.461118	11.726679
H	6.603963	15.255248	11.983592
C	10.698905	19.414977	16.714852
H	10.377092	18.917945	15.791071
C	11.514827	18.475295	13.345580
H	11.031598	17.623286	13.838331
H	12.215843	18.933693	14.050944
H	12.094887	18.085081	12.501547
C	7.680372	12.769399	18.594604
C	2.664227	18.174153	14.922085
C	3.111039	17.980195	16.254990
H	3.988616	18.445005	16.682114
C	7.914908	13.499248	19.762067
H	7.278091	13.337250	20.628072
C	8.784785	11.016549	16.168580
C	5.016795	10.146189	11.048933
H	4.540575	10.529658	10.140072
H	4.280996	9.515482	11.558041
H	5.855584	9.511135	10.740620
C	8.929214	14.440907	19.825460
H	9.089279	15.012892	20.736055
C	11.162187	20.739459	12.286131
H	11.834087	20.483944	11.459629
H	11.764389	21.231950	13.057667
H	10.435949	21.467802	11.909722
C	5.191403	18.431802	19.418766
H	5.364602	18.035615	20.425516
H	4.147798	18.222356	19.159428
H	5.314641	19.519578	19.466595
C	2.257562	17.054788	16.902037
H	2.350774	16.700569	17.921611
C	9.731186	14.674844	18.715499
H	10.497048	15.442156	18.774477
C	10.464582	14.210385	16.330807
C	6.496621	11.814597	18.532878

H	6.636705	11.144449	17.678136
C	4.278068	12.196303	12.328222
H	4.564688	12.931532	13.086294
H	3.446157	11.605631	12.728206
H	3.916725	12.731999	11.443130
C	10.313362	15.007727	12.439943
H	10.381279	15.501687	11.463062
H	11.254392	15.197566	12.972177
H	9.505621	15.482268	13.006454
C	10.093072	13.496972	12.296048
H	10.019627	13.075354	13.305084
C	3.109262	14.459347	15.953878
C	1.510534	17.351308	14.758092
H	0.931354	17.262736	13.845795
C	1.251996	16.665854	15.968552
H	0.450964	15.959930	16.145527
C	10.475934	19.496642	12.863734
H	9.893849	19.818520	13.734158
C	11.299771	12.833807	11.618501
H	11.173008	11.749055	11.532449
H	12.211659	13.021785	12.195984
H	11.455155	13.231985	10.609476
C	3.012969	15.380645	13.601388
C	4.952484	18.062575	10.753091
H	4.960873	19.141399	10.947782
H	3.947406	17.687281	10.974159
H	5.139155	17.910893	9.683480
C	5.208966	12.604066	18.274797
H	5.025863	13.321324	19.084630
H	4.345423	11.934383	18.211999
H	5.264204	13.154034	17.330484
C	12.166569	19.044299	16.951960
H	12.595583	19.612472	17.785037
H	12.760715	19.287877	16.066167
H	12.291327	17.978092	17.161717
C	6.367723	10.940079	19.784614
H	7.301360	10.416655	20.021638
H	5.583760	10.190724	19.636014
H	6.085779	11.529984	20.663412
C	10.602802	20.930981	16.486259
H	9.622730	21.242681	16.116979
H	11.344167	21.244427	15.741460
H	10.813060	21.475491	17.414547
C	3.238872	19.127354	13.917234
H	4.328905	19.168060	14.011129
H	2.844865	20.141195	14.060260
H	2.993969	18.816050	12.896617
C	6.082343	15.060552	14.952572
C	9.407191	10.355707	17.374051
H	10.140997	11.015468	17.844314
H	9.893530	9.421124	17.092057
H	8.643264	10.138993	18.127214
C	11.126127	15.588599	16.357069
H	10.384581	16.378467	16.509737
H	11.627691	15.773015	15.400414
H	11.887820	15.657986	17.143978
C	11.539541	13.124441	16.182079
H	12.156278	13.062137	17.086674
H	12.200882	13.363382	15.340695
H	11.112574	12.136073	15.989088
H	9.838843	14.194117	15.432094

3-Mn'.log

Lowest Frequency = 21.4048cm-1

Mn	2.283265	2.232461	-1.201995
Al	-1.663095	1.346603	0.781969
Al	0.836571	-2.371318	0.385794
O	1.404505	-0.896371	-0.416635
O	-0.672287	-1.612970	0.973974
N	-2.620007	1.690677	2.394578

O	-0.202262	2.272679	0.222854
N	0.318732	-4.047087	-0.358232
N	2.238771	-3.031134	1.478939
N	-3.024999	1.992462	-0.402002
C	-0.595125	-0.291058	0.646053
C	-3.905803	2.058561	2.439657
C	3.329628	-1.343360	2.925997
C	-0.524684	-4.102198	-1.529660
C	-1.940743	1.518558	3.657799
C	-4.286013	2.221730	-0.028887
C	-1.915578	-4.271482	-1.395019
C	-2.633953	-4.225522	-0.050633
H	-1.879502	-4.223298	0.743474
C	3.386832	-2.217715	1.834399
C	-2.643984	2.397005	-1.738990
C	0.620024	-5.205909	0.240552
C	-4.712801	2.200407	1.305076
H	-5.754779	2.437292	1.480858
C	-3.426838	-2.916620	0.078616
H	-2.756916	-2.056921	0.000321
H	-3.924629	-2.869176	1.055316
H	-4.199732	-2.849106	-0.697350
C	2.276864	-4.303535	1.888394
C	2.028420	-0.999322	3.628770
H	1.210550	-1.514751	3.111580
C	-2.597743	-0.965049	3.590004
H	-2.490407	-0.829045	2.509472
C	-2.352972	3.757037	-1.971936
C	-1.865603	-2.267740	3.925696
H	-0.785951	-2.165929	3.783916
H	-2.215092	-3.061662	3.256695
H	-2.055637	-2.595153	4.954981
C	-1.976986	0.250922	4.274416
C	1.452205	-5.306834	1.361786
H	1.572905	-6.297369	1.782302
C	0.084702	-3.995543	-2.795253
C	-2.670193	-4.434686	-2.559863
H	-3.745549	-4.577501	-2.480229
C	-1.145354	3.953842	3.536105
H	-1.804911	3.949154	2.660026
C	-2.838952	-0.032148	-2.541977
H	-3.064156	-0.180496	-1.479799
C	4.571242	-2.381472	1.091169
C	-0.712727	-4.170561	-3.926819
H	-0.263501	-4.107171	-4.913497
C	-2.076247	-4.410088	-3.813488
H	-2.680465	-4.549094	-4.706036
C	-1.293069	2.615930	4.249065
C	-2.414166	4.821192	-0.880854
H	-2.820420	4.361701	0.027326
C	-2.566298	1.446369	-2.768174
C	-1.605659	-0.876752	-2.875758
H	-1.341445	-0.792708	-3.937144
H	-1.810296	-1.928785	-2.669615
H	-0.737525	-0.572658	-2.281758
C	4.513446	-0.731572	3.344874
H	4.490070	-0.062277	4.201655
C	-4.539298	2.405832	3.765725
H	-4.298319	1.684233	4.547722
H	-5.622675	2.483481	3.664232
H	-4.153455	3.376820	4.097129
C	0.062166	-6.499064	-0.302140
H	-1.014786	-6.557527	-0.124925
H	0.540816	-7.352296	0.179353
H	0.211186	-6.565954	-1.383065
C	2.016621	-1.462657	5.089191
H	2.142510	-2.548623	5.169800
H	1.068238	-1.193202	5.566376
H	2.824065	-0.988638	5.658976
C	0.514816	0.085028	-0.058098
C	-1.987452	4.137752	-3.262645
H	-1.749496	5.180507	-3.460659

C	-1.425862	0.137536	5.551652	C	3.299743	-4.737245	2.908966
H	-1.459317	-0.820098	6.064494	H	4.289516	-4.805714	2.446055
C	5.707637	-0.947266	2.677870	H	3.038532	-5.715496	3.315444
H	6.618950	-0.463652	3.018855	H	3.381338	-4.010086	3.720219
C	-3.343367	5.981346	-1.262697	C	1.574281	-3.700388	-2.934119
H	-2.954703	6.536640	-2.123269	H	1.868009	-3.073887	-2.083486
H	-3.425615	6.686729	-0.429151	C	-4.051606	-0.522913	-3.342342
H	-4.353411	5.641928	-1.517843	H	-4.964607	0.024442	-3.083456
C	-3.566411	-5.423619	0.171119	H	-4.225729	-1.587160	-3.145955
H	-4.411924	-5.406496	-0.525375	H	-3.887698	-0.406799	-4.419711
H	-3.979817	-5.392801	1.184582	C	-0.821380	1.223161	6.175783
H	-3.058231	-6.385259	0.044899	H	-0.395934	1.109653	7.169541
C	-2.210700	1.881786	-4.047574	C	0.293175	4.128492	3.028939
H	-2.150181	1.158103	-4.857323	H	1.001619	4.127975	3.865905
C	-4.092453	-1.115863	3.906235	H	0.403878	5.074004	2.487689
H	-4.476318	-2.045826	3.471409	H	0.573651	3.327590	2.338301
H	-4.690546	-0.295776	3.498606	C	-0.735432	2.441267	5.517963
H	-4.260972	-1.158553	4.989216	H	-0.227270	3.274942	5.996176
C	-1.920228	3.213827	-4.297787	C	0.778540	1.531171	-0.281244
H	-1.634287	3.532604	-5.296513	C	-1.562722	5.137933	4.415176
C	-5.313585	2.600085	-1.065808	H	-2.577520	5.017785	4.811172
H	-5.138981	3.620066	-1.421110	H	-1.531437	6.064731	3.833360
H	-6.318911	2.546021	-0.645879	H	-0.886200	5.266036	5.267183
H	-5.246779	1.945608	-1.938262	C	1.906395	-2.892487	-4.191400
C	5.724541	-1.742219	1.539406	H	1.788024	-3.485045	-5.105712
H	6.650270	-1.855907	0.980951	H	2.949324	-2.561349	-4.153328
C	1.758292	0.506542	3.517100	H	1.274957	-2.001282	-4.271635
H	2.498922	1.086969	4.079292	C	2.412616	-4.983863	-2.878258
H	0.770479	0.746483	3.918240	H	2.293091	-5.509685	-1.925085
H	1.799387	0.833511	2.472984	H	3.477356	-4.751807	-2.996967
C	-1.012864	5.343264	-0.541319	H	2.124434	-5.668257	-3.684951
H	-0.360193	4.526455	-0.226705	C	2.500868	0.931060	-2.916026
H	-1.067778	6.083606	0.266078	H	2.562854	-0.145941	-2.834608
H	-0.555304	5.833595	-1.409094	C	3.589405	1.855991	-2.867804
C	4.596831	-3.143654	-0.227096	H	4.636804	1.599432	-2.755296
H	3.695648	-3.765117	-0.290375	C	3.082182	3.172460	-2.982356

C	1.659239	3.065882	-3.091925
H	0.964481	3.891589	-3.189043
C	1.313843	1.690809	-3.056697
H	0.305623	1.301245	-3.111881
C	4.543750	-2.128670	-1.379235
H	3.661090	-1.484710	-1.290922
H	4.521040	-2.641514	-2.349216
H	5.427799	-1.480980	-1.359518
C	5.798517	-4.081877	-0.370065
H	6.742933	-3.529417	-0.418005
H	5.714381	-4.662046	-1.295694
H	5.864975	-4.787421	0.465658
C	3.885471	4.438690	-3.046763
H	3.365427	5.264141	-2.550927
H	4.070491	4.734163	-4.086339
H	4.854176	4.311212	-2.554592
C	2.238712	3.745765	-0.289265
O	2.236561	4.771525	0.259514
C	3.410654	1.451139	-0.076005
O	4.192845	0.952915	0.621569

4-Mn.log

Lowest Frequency = 11.9528cm-1

Mn	12.546094	17.424637	4.213090
Al	8.581049	14.383414	5.141990
Al	8.694784	19.749061	4.729818
O	10.307137	19.047004	4.630120
O	7.696097	18.333143	4.749548
O	9.958479	15.500430	4.988836
C	10.711385	17.779655	4.600711
O	6.102402	16.111241	4.913993
N	8.459740	12.964450	3.881274
N	8.604794	13.310855	6.707757
O	12.578742	14.492988	4.278907
N	8.307981	20.869774	6.197630
N	8.324475	20.971362	3.352114
C	9.215720	11.287768	7.963178
H	9.813025	11.877089	8.663082
H	9.708345	10.332080	7.780383
H	8.253943	11.098091	8.449661
C	7.318903	15.949785	4.958471
O	13.380195	17.771915	6.996813
C	7.049640	22.396422	4.818010
H	6.355093	23.227253	4.848390
C	7.949704	13.266719	2.561084
C	5.577984	13.171716	3.545977
H	6.036299	13.585147	4.450329
C	8.139884	13.848994	7.968033
C	9.595761	16.806219	4.847723
C	8.251204	17.117312	4.854746
C	8.835304	11.720169	4.184870
C	9.198799	11.320329	5.481336
H	9.545841	10.299851	5.586553
C	6.376033	14.065818	9.594851
H	5.353964	13.869903	9.911084
C	8.298190	13.778476	0.236374
H	8.971162	13.945304	-0.601404
C	10.348943	13.439515	1.640277
H	10.600299	13.101231	2.651063
C	6.552195	13.384361	2.391666
C	7.508848	21.927611	6.063843
C	8.951639	20.807943	2.060750
C	9.009763	12.036353	6.670798
C	7.476071	21.989045	3.549838
C	6.810401	13.587108	8.359187
C	8.835666	13.478388	1.491200
C	8.867084	10.661894	3.111571
H	7.988486	10.725809	2.465281
H	8.918364	9.666414	3.554204
H	9.748265	10.804546	2.478518

C	8.784669	20.472386	7.505624
C	10.925878	14.848828	1.487513
H	10.663398	15.276893	0.511675
H	12.017020	14.818250	1.566458
H	10.557801	15.517117	2.271714
C	10.977241	21.657821	6.933563
H	10.691379	21.397646	5.909514
C	11.010748	12.487791	0.634584
H	10.569083	11.485741	0.652928
H	12.079101	12.394676	0.854849
H	10.919218	12.864772	-0.390196
C	7.975902	19.690505	8.349758
C	7.224150	14.784765	10.426178
H	6.872656	15.137205	11.392636
C	10.011948	21.673571	1.718122
C	7.034203	22.674829	7.283655
H	6.149460	22.173526	7.689256
H	6.755669	23.696762	7.021819
H	7.790589	22.691717	8.070181
C	9.236707	19.607002	-0.001585
H	8.938660	18.807049	-0.675258
C	6.930454	13.874245	0.044233
H	6.530666	14.106921	-0.939340
C	4.259903	13.926170	3.362512
H	4.441227	14.986573	3.168750
H	3.667486	13.856431	4.279135
H	3.654145	13.506205	2.551001
C	8.997338	14.616967	8.774536
C	12.453090	15.646896	4.298095
C	6.605822	19.163335	7.944117
H	6.378957	19.526370	6.936495
C	10.503474	22.775695	2.651295
H	9.747036	22.947258	3.424092
C	8.546053	19.770328	1.202957
C	10.092442	20.849050	7.873309
C	10.664295	21.469273	0.503401
H	11.493857	22.115063	0.227232
C	6.069621	13.683744	1.117619
H	5.000074	13.777801	0.960333
C	10.286606	20.441383	-0.351577
H	10.813494	20.292744	-1.290219
C	7.380230	18.841236	1.514538
H	7.029010	19.046817	2.528937
C	5.822645	12.825574	7.483730
H	6.347447	12.481435	6.585487
C	8.485191	19.346627	9.604786
H	7.878723	18.737183	10.271251
C	10.412747	14.995225	8.363296
H	10.587947	14.648636	7.340036
C	8.516350	15.063108	10.008947
H	9.173732	15.644120	10.651508
C	6.990369	22.781409	2.363638
H	6.137163	23.402207	2.640216
H	7.786021	23.433858	1.989233
H	6.707401	22.116644	1.543252
C	5.496705	19.634200	8.893956
H	5.644623	19.228285	9.901006
H	4.522494	19.283747	8.537853
H	5.453682	20.724354	8.986041
C	7.800875	17.366088	1.476717
H	8.034380	17.034595	0.457548
H	6.992285	16.729427	1.854312
H	8.684581	17.187403	2.098069
C	10.552170	20.478833	9.136233
H	11.555668	20.757948	9.443382
C	9.751773	19.744844	10.003000
H	10.128585	19.465270	10.983571
C	12.992573	17.595545	5.913781
C	5.312810	11.679390	3.786946
H	4.890752	11.211159	2.889813
H	4.594105	11.547981	4.604659
H	6.223297	11.135344	4.058606

C	4.683811	13.750080	7.033596
H	4.145738	14.155226	7.898405
H	3.960124	13.190094	6.429285
H	5.055469	14.589650	6.434975
C	10.743289	23.164376	7.106859
H	9.711030	23.449130	6.877627
H	11.400756	23.732912	6.439168
H	10.959691	23.473129	8.136297
C	12.465018	21.327059	7.072132
H	12.882650	21.708512	8.011137
H	13.022881	21.803273	6.257708
H	12.646201	20.248690	7.028808
C	5.261117	11.584703	8.189369
H	6.053738	10.907242	8.524529
H	4.608475	11.026864	7.508944
H	4.665620	11.858770	9.067269
C	10.579354	16.518716	8.363032
H	9.876783	16.998803	7.672382
H	11.591952	16.792046	8.061380
H	10.405236	16.944063	9.357646
C	10.723506	24.108732	1.927683
H	11.555791	24.052653	1.218166
H	10.966594	24.893224	2.651767
H	9.833866	24.423272	1.370865
C	11.782036	22.328436	3.369213
H	11.613831	21.398952	3.922995
H	12.120025	23.098517	4.073491
H	12.589944	22.142514	2.652006
C	6.617496	17.629867	7.881439
H	7.479146	17.267205	7.315553
H	5.716897	17.256322	7.384595
H	6.671011	17.191086	8.884842
C	6.209046	19.094347	0.556728
H	5.857077	20.130758	0.609804
H	5.367927	18.439690	0.807062
H	6.495821	18.893018	-0.482010
C	11.462769	14.344665	9.272727
H	11.347669	14.675762	10.311492
H	12.468903	14.622349	8.942507
H	11.396592	13.251209	9.262287
C	14.460554	18.237569	3.634545
H	15.340183	18.202831	4.266926
C	14.099844	17.252054	2.688327
C	12.875931	17.675616	2.085426
H	12.336014	17.150617	1.308081
C	12.511627	18.926997	2.648957
H	11.614772	19.483914	2.410375
C	14.897808	16.029977	2.338033
H	15.555495	15.744888	3.164386
H	15.521598	16.215923	1.456527
H	14.252514	15.174138	2.118554
C	13.481830	19.282673	3.609108
H	13.489045	20.171999	4.225538

Int-1.log

Lowest Frequency = 15.4924cm-1

Mn	3.182919	18.890724	3.752798
O	0.645181	20.166230	4.498037
C	1.654452	19.675932	4.204176
C	3.460920	17.471460	2.168218
H	3.160551	16.440244	2.300964
C	4.720171	18.016206	2.534973
C	4.691108	19.420873	2.277641
C	3.408007	19.734629	1.770549
H	3.068928	20.723819	1.487879
C	2.643354	18.527742	1.704794
H	1.618943	18.440183	1.365091
C	5.834349	20.376931	2.457016
H	5.472206	21.392791	2.639433
H	6.463589	20.397858	1.560192

H	6.465404	20.086689	3.302205
C	4.066320	19.706515	5.051626
O	4.669698	20.224285	5.898323
C	2.780648	17.529798	4.934461
O	2.777658	16.199245	4.772920
H	5.562318	17.465433	2.936003
Al	2.209225	16.543785	6.473951
N	3.133128	15.703480	7.879150
N	0.464383	16.046943	6.960893
C	2.560790	14.812925	8.693731
C	1.192268	14.501965	8.658168
H	0.853471	13.751953	9.362210
C	0.198040	15.127333	7.889748
C	3.408853	14.091683	9.709751
H	4.038035	13.351072	9.205101
H	2.784268	13.575854	10.440006
H	4.078739	14.782200	10.227827
C	-1.237118	14.771358	8.174935
H	-1.776350	14.555210	7.248901
H	-1.743856	15.621079	8.645219
H	-1.300802	13.912095	8.843379
C	4.522242	16.066936	8.044564
C	5.530821	15.265357	7.484950
C	6.857048	15.668496	7.656865
H	7.655332	15.064733	7.231974
C	7.171477	16.829331	8.345636
H	8.209672	17.125814	8.468258
C	6.157154	17.628834	8.857641
H	6.414731	18.553178	9.364972
C	4.816765	17.275168	8.710066
C	5.234932	14.016934	6.664252
H	4.167186	13.788746	6.756843
C	5.520673	14.271770	5.177400
H	4.903871	15.088006	4.789013
H	5.304359	13.372444	4.590147
H	6.576094	14.526500	5.025679
C	6.020323	12.796199	7.160181
H	5.875866	12.617364	8.231388
H	7.095653	12.917801	6.990691
H	5.704868	11.898491	6.618639
C	3.712314	18.177644	9.251983
H	2.862261	18.110350	8.559322
C	3.214954	17.712402	10.626874
H	2.781635	16.707910	10.593229
H	2.441948	18.393319	11.000780
H	4.037536	17.705755	11.351439
C	4.109374	19.654780	9.300247
H	4.503707	19.994227	8.338068
H	4.857111	19.849487	10.077571
H	3.230630	20.262183	9.541294
C	-0.610493	16.781836	6.331196
C	-1.098359	17.937389	6.970294
C	-2.101479	18.666650	6.334500
H	-2.483446	19.570334	6.802698
C	-2.607488	18.268936	5.104442
H	-3.387881	18.851791	4.623030
C	-2.100530	17.137925	4.483098
H	-2.488487	16.840789	3.511563
C	-1.087614	16.378674	5.073120
C	-0.521603	18.444118	8.286398
H	0.110443	17.657292	8.715359
C	0.369613	19.669185	8.037005
H	1.174246	19.444537	7.327625
H	0.819701	20.015724	8.975171
H	-0.211736	20.493974	7.610913
C	-1.607372	18.761385	9.320383
H	-2.264573	17.902234	9.494272
H	-2.234690	19.600996	9.002903
H	-1.149879	19.038186	10.276022
C	-0.534574	15.171551	4.328611
H	0.286306	14.751391	4.919166
C	-1.597529	14.078741	4.160069

H	-1.991547	13.738783	5.124264
H	-1.173914	13.210280	3.644756
H	-2.443858	14.439628	3.564808
C	0.044016	15.582667	2.968504
H	0.793579	16.371103	3.083710
H	-0.742951	15.945743	2.297310
H	0.523677	14.723849	2.486728

Int-2.log

Lowest Frequency = 15.3826cm-1

Mn	3.608522	18.842359	3.220622
O	1.452691	20.840422	3.287106
C	2.309332	20.064911	3.298799
C	3.721380	16.949509	2.222544
H	3.385553	16.049752	2.721224
C	5.024126	17.506205	2.305188
C	5.035801	18.733572	1.571970
C	3.734311	18.928825	1.058722
H	3.415156	19.780688	0.469935
C	2.914850	17.828281	1.460446
H	1.868006	17.692815	1.220355
C	6.225857	19.618896	1.342205
H	5.915755	20.650035	1.150549
H	6.802198	19.272178	0.477267
H	6.892589	19.624169	2.209368
C	4.725207	19.871824	4.158235
O	5.513340	20.513551	4.708389
C	2.988256	18.055495	4.765064
O	2.742283	16.771448	5.035764
H	5.874025	17.075860	2.821050
Al	2.140596	16.868543	6.767668
N	3.061766	15.817841	8.034677
N	0.413641	16.184504	7.097453
C	2.507807	14.812876	8.718348
C	1.155241	14.460969	8.608160
H	0.827841	13.616956	9.202367
C	0.160905	15.137844	7.886757
C	3.359334	14.029734	9.682074
H	4.225933	13.603187	9.169326
H	2.786775	13.227190	10.147726
H	3.749413	14.689476	10.463341
C	-1.262393	14.676359	8.056071
H	-1.737836	14.516438	7.084968
H	-1.845770	15.447837	8.569219
H	-1.308051	13.755514	8.638210
C	4.432784	16.203816	8.288510
C	5.470907	15.633343	7.529489
C	6.774153	16.073733	7.766424
H	7.591511	15.647337	7.189578
C	7.043804	17.050513	8.714029
H	8.064987	17.383409	8.878092
C	6.004261	17.609048	9.443985
H	6.219392	18.383117	10.176825
C	4.683899	17.203252	9.248121
C	5.229928	14.575989	6.459891
H	4.158146	14.350062	6.436878
C	5.620312	15.100264	5.072053
H	5.066414	16.010033	4.825993
H	5.398734	14.348869	4.305718
H	6.693493	15.319985	5.024079
C	5.977807	13.272821	6.770481
H	5.713932	12.867666	7.753387
H	7.062486	13.426249	6.758407
H	5.743259	12.512458	6.018342
C	3.578863	17.858071	10.068687
H	2.624092	17.393513	9.794801
C	3.784132	17.623837	11.570971
H	3.847203	16.557509	11.814356
H	2.952317	18.052079	12.139898
H	4.705923	18.099144	11.923705

C	3.470483	19.358353	9.764881
H	3.273412	19.554153	8.705780
H	4.395415	19.879929	10.035556
H	2.657242	19.803397	10.349332
C	-0.675828	16.917403	6.489796
C	-1.246442	17.990186	7.201428
C	-2.260608	18.722278	6.584195
H	-2.709690	19.559655	7.112952
C	-2.697322	18.408066	5.304864
H	-3.486732	18.992230	4.840024
C	-2.113742	17.354930	4.616048
H	-2.451520	17.120978	3.609213
C	-1.090425	16.594511	5.185129
C	-0.785797	18.391513	8.597659
H	-0.042067	17.661723	8.938959
C	-0.112114	19.770640	8.581924
H	0.744220	19.809090	7.900284
H	0.239201	20.031243	9.586917
H	-0.820878	20.545083	8.267456
C	-1.942132	18.367541	9.605345
H	-2.438837	17.391518	9.636678
H	-2.701528	19.117019	9.357586
H	-1.573446	18.592500	10.611517
C	-0.467763	15.465480	4.374301
H	0.338057	15.021306	4.968494
C	-1.488959	14.362311	4.067528
H	-1.925431	13.938439	4.978373
H	-1.013136	13.547182	3.512345
H	-2.311812	14.746490	3.454454
C	0.156275	15.995041	3.077170
H	0.891386	16.777835	3.282882
H	-0.610043	16.404937	2.408794
H	0.663619	15.183752	2.542867
C	2.600169	18.741563	6.117306
O	2.662515	19.924509	6.359447

Int-3.log

Lowest Frequency = 12.2529cm-1

Mn	3.417177	15.708189	15.158136
Al	7.467183	17.865144	14.816916
Al	7.445667	13.267047	14.869562
O	5.744229	17.469040	14.932908
O	8.330897	16.349487	14.769989
O	5.732581	13.854626	14.982593
N	7.764861	19.010653	13.331502
N	7.942106	12.042519	13.504232
N	7.926681	19.107538	16.174657
N	8.198005	12.295884	16.317290
C	7.380718	20.279326	13.472875
C	7.832523	12.509332	12.139318
C	5.190321	16.251059	14.941709
C	9.676644	18.260160	19.938311
H	10.126643	18.069162	20.909351
C	6.679828	12.211443	11.389681
C	9.613179	18.832793	11.719838
C	7.432799	17.865219	11.164361
C	8.270491	18.564501	12.050748
C	8.520490	18.781683	17.454756
C	7.452768	20.349934	15.986780
C	8.881294	13.282076	11.605332
C	8.744347	13.767271	10.303359
H	9.537362	14.377392	9.876556
C	10.088924	18.423544	10.474353
H	11.124089	18.624867	10.207999
C	7.553636	15.231669	14.806881
C	8.420867	10.817630	13.738869
C	7.956715	17.480222	9.926970
H	7.317247	16.945712	9.229146
C	7.614459	13.486189	9.549304
H	7.525121	13.872222	8.536670

C	9.905919	18.998900	17.636189
C	9.267307	17.759718	9.574402
H	9.650828	17.455590	8.603671
C	8.711300	9.905689	12.574226
H	7.787781	9.671331	12.035448
H	9.168048	8.975472	12.913441
H	9.378839	10.393790	11.858617
C	6.595495	12.714533	10.090786
H	5.709530	12.504743	9.496862
O	2.705081	15.035016	12.395451
C	7.299311	21.271169	17.170667
H	6.555020	20.859613	17.859974
H	6.976153	22.262926	16.852711
H	8.235227	21.360249	17.727794
C	5.991840	17.499038	11.486011
H	5.778278	17.763466	12.526134
C	5.530300	11.384391	11.949757
H	5.875369	10.901257	12.870884
C	7.730299	18.260657	18.492504
C	7.098512	20.856414	14.727660
H	6.697008	21.861955	14.711598
C	6.099318	16.382091	18.370628
H	6.747292	15.884381	17.639819
H	5.069072	16.088777	18.151951
H	6.365078	15.995695	19.360347
C	9.392384	13.900584	17.732489
C	8.318651	12.999269	17.573609
C	8.333275	18.006806	19.727929
H	7.730305	17.598847	20.535383
C	7.293404	21.176322	12.265908
H	8.299636	21.470173	11.948477
H	6.729706	22.080440	12.499678
H	6.828485	20.660648	11.422515
C	10.454108	18.743035	18.893008
H	11.512979	18.910039	19.060674
C	6.261450	17.907427	18.323927
H	5.928133	18.250281	17.338097
C	8.689742	10.325367	15.023061
H	9.050562	9.306085	15.081919
C	5.776785	15.989624	11.331469
H	5.939000	15.667677	10.297391
H	4.754561	15.721102	11.601548
H	6.463741	15.419789	11.967699
C	10.807941	19.468342	16.497330
H	10.444501	18.976571	15.586594
C	11.614988	18.463505	13.161271
H	11.130833	17.640585	13.700045
H	12.353051	18.927367	13.823193
H	12.154191	18.031218	12.310767
C	7.362387	12.802029	18.584323
C	2.367581	17.534041	15.551853
C	3.003422	17.088017	16.745926
H	3.779713	17.633146	17.264213
C	7.545477	13.469125	19.797564
H	6.824209	13.324749	20.598348
C	8.637214	11.038422	16.229344
C	5.091119	10.275254	10.986072
H	4.622056	10.686893	10.085997
H	4.350328	9.631126	11.470695
H	5.931219	9.648341	10.664951
C	8.615181	14.330485	19.988434
H	8.735706	14.849732	20.936282
C	11.285686	20.715204	12.072223
H	11.938669	20.431151	11.239830
H	11.909849	21.210173	12.824574
H	10.567751	21.450335	11.693221
C	5.380203	18.578549	19.385801
H	5.626920	18.223798	20.392394
H	4.324628	18.346740	19.210897
H	5.490594	19.668570	19.383949
C	2.480389	15.817302	17.099521
H	2.794752	15.211025	17.942522

C	9.516431	14.558788	18.956444
H	10.319456	15.275041	19.104238
C	10.406059	14.144940	16.618078
C	6.121357	11.942308	18.380325
H	6.270239	11.331755	17.482988
C	4.350386	12.288910	12.321440
H	4.634938	13.003133	13.100584
H	3.513056	11.690980	12.699324
H	3.995395	12.852656	11.451701
C	10.446428	15.063362	12.511263
H	10.549346	15.553349	11.535196
H	11.386078	15.216817	13.057312
H	9.648643	15.568228	13.064853
C	10.171929	13.562459	12.365939
H	10.075593	13.145188	13.375090
C	1.443203	16.519381	15.187679
H	0.803835	16.549702	14.312302
C	1.499721	15.456597	16.138631
H	0.902585	14.554489	16.131461
C	10.585618	19.495929	12.683626
H	10.025912	19.843419	13.558797
C	11.359369	12.856806	11.696328
H	11.199503	11.776105	11.614542
H	12.275342	13.019131	12.274849
H	11.528162	13.245383	10.685705
C	3.076407	15.240244	13.481730
C	5.000734	18.275136	10.609423
H	5.100901	19.358963	10.737302
H	3.975084	17.997956	10.874207
H	5.147922	18.047613	9.547035
C	4.897528	12.834457	18.123052
H	4.729173	13.508505	18.972487
H	3.998797	12.220443	17.993875
H	5.030695	13.434380	17.215812
C	12.269795	19.053311	16.694753
H	12.736355	19.599997	17.521838
H	12.849359	19.289060	15.797833
H	12.367602	17.981303	16.891108
C	5.862828	10.988891	19.552741
H	6.743647	10.383643	19.795595
H	5.039153	10.310108	19.309413
H	5.576034	11.533064	20.459133
C	10.748932	20.985581	16.265708
H	9.766397	21.323932	15.927467
H	11.474036	21.275367	15.495679
H	11.004787	21.526531	17.184609
C	2.616765	18.833379	14.844838
H	3.687459	19.062398	14.842094
H	2.081805	19.663973	15.322304
H	2.284592	18.774901	13.803321
C	6.194607	15.183296	14.901224
C	9.117508	10.327846	17.470325
H	9.816021	10.950507	18.035173
H	9.602142	9.385445	17.212401
H	8.271459	10.115736	18.132072
C	11.007274	15.551358	16.651103
H	10.222800	16.312272	16.697974
H	11.588246	15.721229	15.737069
H	11.685514	15.688453	17.502654
C	11.517526	13.087181	16.621655
H	12.049480	13.086749	17.580436
H	12.246171	13.300192	15.831118
H	11.128251	12.079098	16.446806
H	9.875507	14.068672	15.663197

Int-4.log

Lowest Frequency = 14.9412cm-1

Mn	3.623379	19.194477	4.482132
Al	0.190642	18.529300	6.084539
Al	2.347118	15.297364	5.597250

O	3.021717	16.311883	4.223012
O	1.572943	17.989404	7.036480
N	-1.065498	18.589472	7.554834
O	1.117680	20.163573	5.506633
N	1.505986	13.609750	5.221512
N	3.808986	14.545231	6.637963
N	-1.204504	18.857112	4.737633
C	1.771348	17.142542	5.909519
C	-2.392213	18.536421	7.457814
C	5.249444	16.161811	7.856318
C	0.591757	13.461915	4.110950
C	-0.498148	18.651294	8.886061
C	-2.514105	18.687277	4.965514
C	-0.797017	13.545924	4.316855
C	-1.416385	13.898922	5.662801
H	-0.625612	13.882063	6.421508
C	5.014377	15.342004	6.738604
C	-0.846449	19.516252	3.493686
C	1.733635	12.513891	5.970312
C	-3.067436	18.480077	6.231602
H	-4.143665	18.367429	6.276876
C	-1.985097	15.320383	5.619143
H	-1.187881	16.047325	5.431288
H	-2.476786	15.577405	6.563489
H	-2.729840	15.421232	4.819757
C	3.767234	13.340305	7.185125
C	4.372445	16.152021	9.101820
H	3.573822	15.418753	8.946701
C	-0.549509	16.085313	9.024504
H	-0.947900	16.220412	8.015267
C	-0.855646	20.930879	3.481605
C	0.787062	15.346474	8.886809
H	1.479839	15.968476	8.313450
H	0.653031	14.377656	8.385574
H	1.230811	15.148454	9.870615
C	-0.313039	17.466089	9.622256

C	2.718512	12.426204	6.951518
H	2.776667	11.490048	7.493345
C	1.122699	13.195442	2.829617
C	-1.646590	13.314856	3.231486
H	-2.722763	13.378773	3.374868
C	-0.212432	21.185858	8.608988
H	-0.601687	20.943256	7.614997
C	-0.601400	17.273732	2.251739
H	-0.886573	16.886764	3.236894
C	5.928400	15.277912	5.668030
C	0.231541	12.939115	1.787350
H	0.616099	12.717170	0.796864
C	-1.143086	12.990503	1.982103
H	-1.818792	12.791918	1.154106
C	-0.134123	19.898290	9.416756
C	-1.308683	21.759423	4.679778
H	-1.322582	21.107557	5.557410
C	-0.518673	18.789313	2.339502
C	0.745446	16.652424	1.874105
H	1.100155	17.040314	0.910743
H	0.632486	15.569022	1.774100
H	1.511908	16.842289	2.628584
C	6.386654	16.973042	7.843431
H	6.571194	17.633860	8.686358
C	-3.242830	18.559686	8.704130
H	-3.013078	17.714681	9.357468
H	-4.302879	18.537543	8.448794
H	-3.033644	19.464418	9.283362
C	0.917239	11.265909	5.729409
H	-0.139572	11.444626	5.941672
H	1.268606	10.450941	6.362890
H	0.983824	10.955349	4.682791
C	5.184848	15.707829	10.328611
H	5.717516	14.765626	10.160858
H	4.523767	15.580958	11.193106
H	5.931954	16.463166	10.596468

C	2.750957	17.457916	4.911186
C	-0.491576	21.589147	2.309512
H	-0.494836	22.676064	2.285965
C	0.168847	17.565774	10.926215
H	0.311991	16.659093	11.510706
C	7.275626	16.957663	6.780148
H	8.152759	17.599846	6.793054
C	-2.739055	22.278153	4.472590
H	-2.796346	22.918268	3.584502
H	-3.058990	22.870774	5.336824
H	-3.456294	21.461179	4.343600
C	-2.510589	12.914292	6.096128
H	-3.391448	12.983924	5.448611
H	-2.837683	13.144467	7.116027
H	-2.171127	11.873662	6.076874
C	-0.155789	19.498244	1.188174
H	0.100854	18.945682	0.287305
C	-1.553801	15.243001	9.819293
H	-1.710139	14.276914	9.324118
H	-2.529606	15.731705	9.911817
H	-1.190031	15.036437	10.832102
C	-0.131745	20.882447	1.167912
H	0.150956	21.412817	0.262239
C	-3.496164	18.715399	3.817035
H	-3.259346	19.482848	3.079056
H	-4.513381	18.867570	4.181145
H	-3.457078	17.747353	3.304985
C	7.054685	16.098520	5.711148
H	7.776567	16.056687	4.898146
C	3.711399	17.507520	9.374641
H	4.467791	18.285156	9.529259
H	3.100849	17.446263	10.284278
H	3.059827	17.818901	8.551404
C	-0.365475	22.925297	4.993939
H	0.663703	22.577694	5.113452
H	-0.675128	23.411167	5.927113
H	-0.387192	23.690564	4.209908
C	5.779571	14.274723	4.530939
H	4.784157	13.825410	4.600256
C	4.869731	12.861670	8.096811
H	5.825839	13.336054	7.871933
H	4.975457	11.777304	8.031235
H	4.608700	13.114068	9.130681
C	2.626478	13.206810	2.566593
H	3.052132	14.012442	3.175807
C	-1.668773	16.836337	1.236244
H	-2.638241	17.312109	1.414083
H	-1.801867	15.749657	1.279717
H	-1.362800	17.093608	0.215537
C	0.490094	18.796603	11.484764
H	0.864697	18.854158	12.503381
C	1.185132	21.788902	8.418977
H	1.602817	22.124248	9.375312
H	1.139510	22.654558	7.748737
H	1.873137	21.055315	7.987615
C	0.354207	19.947765	10.724760
H	0.636662	20.908441	11.149809
C	2.206039	19.882810	5.034356
C	-1.175031	22.201687	9.234117
H	-2.186147	21.791264	9.333880
H	-1.236106	23.102516	8.612873
H	-0.839209	22.508222	10.231223
C	2.975306	13.539265	1.112379
H	2.713439	12.722478	0.429704
H	4.053475	13.701373	1.022406
H	2.470652	14.449616	0.773964
C	3.300604	11.888240	2.970743
H	3.223816	11.690420	4.044121
H	4.367469	11.917266	2.719161
H	2.852920	11.045148	2.430730
C	4.140515	18.404272	2.541713
H	3.720955	17.466801	2.203376

C	5.321210	18.544254	3.317502
H	5.985298	17.749002	3.630056
C	5.483776	19.929988	3.630152
C	4.390246	20.623589	3.061216
H	4.211335	21.688658	3.145844
C	3.554140	19.680313	2.385736
H	2.627627	19.901377	1.869517
C	5.887664	14.917637	3.146782
H	5.090559	15.654034	3.009760
H	5.796345	14.149494	2.369585
H	6.855496	15.410330	2.998556
C	6.800616	13.139777	4.692429
H	7.825555	13.521141	4.618704
H	6.665382	12.384753	3.909343
H	6.700293	12.641470	5.663048
C	6.638761	20.533490	4.375026
H	6.342692	21.453960	4.886600
H	7.455581	20.776303	3.686118
H	7.026940	19.838412	5.126278
C	4.322690	19.326569	6.114580
O	4.817820	19.537147	7.138063

Int-5.log

Lowest Frequency = 14.6866cm-1

Mn	1.697587	2.098079	-1.246071
Al	-1.560763	1.586857	0.502988
Al	0.863689	-2.082056	0.144745
O	1.597592	-0.780291	-0.792270
O	-0.532584	-1.140898	0.622045
N	-2.571965	1.687514	2.135959
O	-0.446946	3.139775	0.361475
N	0.271671	-3.799154	-0.472888
N	2.241065	-2.750744	1.290137
N	-2.986194	2.154497	-0.667928
C	-0.284377	0.183294	0.166180
C	-3.879994	1.968360	2.180794
C	3.439450	-1.054440	2.643208
C	-0.571680	-3.902134	-1.642193
C	-1.917482	1.467537	3.409504
C	-4.262552	2.245478	-0.285164
C	-1.968520	-4.003941	-1.504795
C	-2.684404	-3.835951	-0.171094
H	-1.931927	-3.844729	0.624586
C	3.409960	-1.935485	1.552613
C	-2.682556	2.559745	-2.025831
C	0.520173	-4.923074	0.210027
C	-4.682112	2.141317	1.046609
H	-5.739997	2.284250	1.227052
C	-3.373003	-2.464702	-0.127174
H	-2.621290	-1.673115	-0.202460
H	-3.914984	-2.338033	0.817662
H	-4.097886	-2.366773	-0.945281
C	2.239581	-3.991723	1.777488
C	2.233447	-0.816613	3.534873
H	1.458439	-1.540485	3.259590
C	-2.475830	-1.034379	3.202695
H	-2.393703	-0.820211	2.133752
C	-2.435813	3.925777	-2.279506
C	-1.648617	-2.301344	3.437561
H	-0.589969	-2.119081	3.233995
H	-1.989829	-3.089097	2.756423
H	-1.753168	-2.687151	4.458533
C	-1.923735	0.169020	3.960423
C	1.361435	-4.988580	1.328985
H	1.440912	-5.953745	1.813462
C	0.037854	-3.905807	-2.912840
C	-2.732157	-4.205560	-2.657344
H	-3.812599	-4.295386	-2.568942
C	-1.220873	3.942622	3.486555
H	-1.709181	3.935220	2.506815

C	-2.860745	0.124678	-2.829803
H	-3.082480	-0.028391	-1.768331
C	4.514325	-2.060858	0.688748
C	-0.768119	-4.115605	-4.032593
H	-0.317260	-4.135330	-5.020148
C	-2.140911	-4.284691	-3.909253
H	-2.751486	-4.451263	-4.792786
C	-1.347491	2.553202	4.096738
C	-2.432428	4.988705	-1.185173
H	-2.431782	4.480991	-0.217359
C	-2.678687	1.615008	-3.066664
C	-1.560905	-0.625522	-3.143206
H	-1.276541	-0.491828	-4.195014
H	-1.694817	-1.695358	-2.966739
H	-0.744038	-0.267176	-2.509307
C	4.608980	-0.329503	2.879025
H	4.642202	0.365086	3.714783
C	-4.564814	2.172724	3.512700
H	-4.276480	1.429610	4.256553
H	-5.648780	2.162021	3.390161
H	-4.274552	3.151386	3.910487
C	-0.105090	-6.224231	-0.233703
H	-1.185274	-6.210335	-0.066294
H	0.322148	-7.061461	0.319111
H	0.049316	-6.385235	-1.304127
C	2.544640	-1.018540	5.022009
H	2.952720	-2.015143	5.226326
H	1.629581	-0.895977	5.612266
H	3.270298	-0.281736	5.384029
C	0.864118	0.378547	-0.563062
C	-2.222947	4.324016	-3.600436
H	-2.041066	5.374303	-3.815280
C	-1.425036	0.005854	5.253829
H	-1.438108	-0.981019	5.708706
C	5.715573	-0.459565	2.055856
H	6.615030	0.117234	2.253574
C	-3.688145	5.869097	-1.252464
H	-3.767078	6.368206	-2.225231
H	-3.649953	6.644955	-0.480096
H	-4.604453	5.290838	-1.095750
C	-3.699477	-4.949512	0.114999
H	-4.550057	-4.904351	-0.574069
H	-4.098163	-4.838679	1.129306
H	-3.262911	-5.950597	0.032386
C	-2.468799	2.067096	-4.371740
H	-2.467661	1.347444	-5.187059
C	-3.948182	-1.304321	3.546330
H	-4.277057	-2.245958	3.091711
H	-4.613647	-0.516815	3.179934
H	-4.087961	-1.393595	4.630708
C	-2.251030	3.409170	-4.644196
H	-2.090614	3.741186	-5.666470
C	-5.339941	2.477794	-1.317253
H	-5.123503	3.343053	-1.946935
H	-6.310719	2.614004	-0.839378
H	-5.395057	1.610526	-1.983242
C	5.659062	-1.313572	0.963230
H	6.520034	-1.397541	0.303550
C	1.673418	0.587670	3.280584
H	2.440888	1.350155	3.458516
H	0.830966	0.786990	3.946769
H	1.327206	0.689577	2.246283
C	-1.172945	5.861849	-1.242068
H	-0.264447	5.253023	-1.253660
H	-1.132344	6.517265	-0.365437
H	-1.167223	6.503625	-2.130620
C	4.496159	-2.947603	-0.548968
H	3.548639	-3.497442	-0.567114
C	3.262254	-4.389812	2.811827
H	4.267164	-4.376398	2.378347
H	3.056722	-5.390529	3.194114
H	3.268839	-3.677489	3.641544

C	1.537453	-3.685204	-3.072733
H	1.849302	-2.982628	-2.291065
C	-4.023397	-0.462908	-3.638990
H	-4.973502	0.042506	-3.432739
H	-4.141970	-1.525760	-3.399653
H	-3.837602	-0.390567	-4.716542
C	-0.907469	1.078266	5.970575
H	-0.528831	0.928254	6.978426
C	0.256793	4.290879	3.261691
H	0.788625	4.373688	4.216836
H	0.347900	5.248747	2.738448
H	0.758526	3.528579	2.658783
C	-0.849986	2.333009	5.383772
H	-0.409829	3.162591	5.932242
C	0.523534	2.853114	-0.349981
C	-1.901867	5.020016	4.339221
H	-2.962739	4.804085	4.505333
H	-1.830099	5.994415	3.844751
H	-1.425813	5.113436	5.321606
C	1.901315	-3.028998	-4.407499
H	1.779347	-3.717477	-5.251699
H	2.949622	-2.715331	-4.393017
H	1.287201	-2.142030	-4.596403
C	2.324065	-4.988871	-2.884271
H	2.192330	-5.406423	-1.880602
H	3.396375	-4.815600	-3.032143
H	2.001222	-5.741502	-3.613527
C	1.867696	1.065568	-3.153969
H	1.540431	0.038830	-3.255297
C	3.152062	1.468511	-2.723683
H	3.983872	0.813324	-2.495306
C	3.162703	2.899558	-2.614569
C	1.871275	3.349039	-2.979528
H	1.546903	4.383408	-2.983006
C	1.062773	2.220247	-3.308922
H	0.025657	2.244788	-3.614315
C	4.552894	-2.083094	-1.814799
H	3.702366	-1.392333	-1.835833
H	4.530291	-2.711995	-2.713116
H	5.479295	-1.497112	-1.845855
C	5.621708	-3.988246	-0.534666
H	6.608672	-3.513367	-0.561919
H	5.545850	-4.643161	-1.410079
H	5.579734	-4.618062	0.360960
C	4.345550	3.755572	-2.266860
H	4.023605	4.718652	-1.859756
H	4.960422	3.951360	-3.152997
H	4.980997	3.271010	-1.519237
C	2.850569	2.059622	0.108555
O	3.634843	2.099440	0.958111

Int-6.log

Lowest Frequency = 19.5630cm-1

Mn	2.249072	1.655742	-1.478184
Al	-1.694506	1.468495	0.608042
Al	0.769448	-2.258669	0.159850
O	1.303638	-0.765395	-0.652913
O	-0.737180	-1.551017	0.801918
N	-2.686494	1.845819	2.194933
O	-0.249623	2.395073	-0.010134
N	0.307671	-3.958880	-0.542742
N	2.259400	-2.826620	1.179264
N	-3.021818	2.120143	-0.611483
C	-0.676880	-0.207854	0.514780
C	-3.972011	2.220226	2.203210
C	3.262560	-0.954239	2.446302
C	-0.576338	-4.054489	-1.681274
C	-2.033488	1.680516	3.472773
C	-4.286111	2.377482	-0.276748
C	-1.961402	-4.221081	-1.495444

C	-2.634185	-4.159935	-0.128863
H	-1.853745	-4.100048	0.637241
C	3.355777	-1.911958	1.425888
C	-2.590406	2.497523	-1.940897
C	0.692697	-5.092906	0.057901
C	-4.746721	2.370837	1.047692
H	-5.789885	2.620607	1.196372
C	-3.477302	-2.881570	-0.013991
H	-2.848155	-1.997812	-0.145846
H	-3.940623	-2.820731	0.978630
H	-4.280576	-2.872754	-0.761156
C	2.402633	-4.087223	1.590717
C	1.983051	-0.722348	3.234388
H	1.156078	-1.206278	2.701067
C	-2.656923	-0.813128	3.402099
H	-2.558164	-0.677019	2.321165
C	-2.208195	3.835766	-2.166896
C	-1.910828	-2.108276	3.736091
H	-0.833687	-1.997797	3.581478
H	-2.259311	-2.907519	3.073299
H	-2.088779	-2.432724	4.768515
C	-2.047640	0.409725	4.083688
C	1.595654	-5.138515	1.127345
H	1.789359	-6.116012	1.551526
C	-0.013589	-3.963104	-2.969850
C	-2.757708	-4.394198	-2.630829
H	-3.829857	-4.532783	-2.510314
C	-1.319186	4.141259	3.385442
H	-2.027602	4.146886	2.548706
C	-2.843210	0.070676	-2.728764
H	-3.076872	-0.063017	-1.666339
C	4.498551	-2.002594	0.609935
C	-0.850570	-4.152630	-4.069737
H	-0.436450	-4.102313	-5.072542
C	-2.209843	-4.385490	-3.905208
H	-2.846294	-4.532548	-4.773751
C	-1.416606	2.788379	4.078912
C	-2.172347	4.889002	-1.063923
H	-2.464746	4.410211	-0.122846
C	-2.542616	1.541838	-2.966298
C	-1.612890	-0.787214	-3.043301
H	-1.377194	-0.760101	-4.114686
H	-1.807207	-1.828691	-2.775824
H	-0.731170	-0.442182	-2.492770
C	4.382894	-0.165563	2.712487
H	4.333902	0.579335	3.501557
C	-4.639433	2.567883	3.512672
H	-4.415545	1.847095	4.300606
H	-5.720278	2.641618	3.384432
H	-4.266260	3.540696	3.853639
C	0.147833	-6.413182	-0.428140
H	-0.919864	-6.489339	-0.205849
H	0.665316	-7.243005	0.054078
H	0.253310	-6.504605	-1.512456
C	2.052029	-1.345621	4.633362
H	2.181580	-2.433180	4.587766
H	1.130759	-1.137280	5.189550
H	2.891620	-0.929171	5.202251
C	0.403663	0.176025	-0.209310
C	-1.833825	4.202200	-3.458903
H	-1.537764	5.230495	-3.654869
C	-1.488195	0.297091	5.358072
H	-1.500673	-0.665109	5.862897
C	5.542667	-0.285594	1.963198
H	6.397834	0.348708	2.178386
C	-3.163964	6.028841	-1.333075
H	-2.916699	6.556362	-2.261150
H	-3.132166	6.760092	-0.518232
H	-4.195256	5.670094	-1.419400
C	-3.506100	-5.388982	0.159776
H	-4.364108	-5.438142	-0.519827
H	-3.900540	-5.336998	1.180002

H	-2.956107	-6.330535	0.060500
C	-2.164115	1.959888	-4.245055
H	-2.130065	1.232337	-5.053168
C	-4.148486	-0.973600	3.728156
H	-4.531028	-1.904073	3.293352
H	-4.752762	-0.154639	3.327046
H	-4.309323	-1.019677	4.812165
C	-1.820190	3.278837	-4.496927
H	-1.523795	3.585650	-5.496316
C	-5.280316	2.750771	-1.347893
H	-5.018068	3.708995	-1.804590
H	-6.286984	2.820484	-0.933934
H	-5.269551	2.009318	-2.151455
C	5.586088	-1.181167	0.904506
H	6.476694	-1.231693	0.282534
C	1.645535	0.770464	3.314470
H	2.345476	1.311867	3.961124
H	0.644984	0.901732	3.732108
H	1.674425	1.235256	2.324061
C	-0.755136	5.444410	-0.869353
H	-0.056740	4.634261	-0.640681
H	-0.745040	6.162847	-0.040703
H	-0.409729	5.973662	-1.765804
C	4.552866	-2.881151	-0.633519
H	3.682573	-3.548082	-0.633069
C	3.522707	-4.449523	2.532767
H	4.464731	-4.529662	1.980559
H	3.321923	-5.410679	3.009084
H	3.665050	-3.680810	3.295488
C	1.466610	-3.659043	-3.172120
H	1.807470	-3.076925	-2.308547
C	-4.052269	-0.420764	-3.533240
H	-4.966062	0.129666	-3.283941
H	-4.230023	-1.483400	-3.331102
H	-3.881334	-0.311790	-4.610332
C	-0.902342	1.388137	5.988815
H	-0.468649	1.274536	6.978918
C	0.086414	4.344095	2.800877
H	0.845179	4.287297	3.590157
H	0.163306	5.328903	2.326160
H	0.322900	3.592523	2.040373
C	-0.851212	2.615330	5.344105
H	-0.364622	3.456470	5.831206
C	0.687457	1.591134	-0.505419
C	-1.698120	5.303589	4.310061
H	-2.677827	5.152102	4.777084
H	-1.732133	6.238437	3.741415
H	-0.964272	5.439014	5.111915
C	1.727087	-2.788076	-4.404299
H	1.540257	-3.327292	-5.339707
H	2.774922	-2.471022	-4.418091
H	1.102565	-1.888304	-4.391400
C	2.305304	-4.942221	-3.219929
H	2.233345	-5.509550	-2.285702
H	3.362924	-4.704454	-3.382486
H	1.974982	-5.591172	-4.039568
C	2.828674	1.191842	-3.507996
H	3.014462	0.175881	-3.840728
C	3.791855	2.061715	-2.952850
H	4.847046	1.851257	-2.825138
C	3.125772	3.280112	-2.573088
C	1.760604	3.134176	-2.910566
H	0.987748	3.874151	-2.739364
C	1.564229	1.840834	-3.489678
H	0.625972	1.442627	-3.855006
C	4.454449	-1.991328	-1.881458
H	3.548722	-1.374366	-1.855590
H	4.449466	-2.602787	-2.792480
H	5.310983	-1.309838	-1.938975
C	5.802108	-3.766770	-0.692374
H	6.716356	-3.169275	-0.774371
H	5.759568	-4.422140	-1.569301

H	5.897905	-4.399855	0.196649
C	3.774259	4.511800	-2.011891
H	3.032877	5.137527	-1.504969
H	4.237562	5.110336	-2.805406
H	4.550869	4.260140	-1.282929
C	3.253332	2.028869	-0.063148
O	3.923597	2.419102	0.807256

TS-1.log

Lowest Frequency = -205.5151cm-1

Mn	3.366321	18.384571	3.354296
O	1.143677	20.101649	4.234355
C	2.022554	19.431432	3.895048
C	3.328458	16.908161	1.801152
H	2.934585	15.914504	1.973750
C	4.671167	17.317639	2.021879
C	4.763485	18.712235	1.728955
C	3.474507	19.153611	1.345341
H	3.215097	20.168269	1.068635
C	2.582865	18.036453	1.388057
H	1.526878	18.052863	1.150060
C	6.013667	19.542234	1.760935
H	5.782893	20.594258	1.950999
H	6.539428	19.478932	0.801955
H	6.698554	19.199951	2.542176
C	4.507261	19.108187	4.522466
O	5.276447	19.564304	5.255070
C	2.887643	17.065535	4.577292
O	2.706245	15.865353	4.719864
H	5.489275	16.684535	2.342887
Al	2.248278	17.007040	6.656789
N	3.134172	15.852992	7.928946
N	0.483831	16.267800	6.969667
C	2.560382	14.827863	8.564762
C	1.205477	14.500252	8.440618
H	0.862866	13.650530	9.018098
C	0.218495	15.223227	7.754012
C	3.378973	14.007380	9.530099
H	4.318722	13.685647	9.075298
H	2.821352	13.132275	9.865912
H	3.642447	14.611981	10.404599
C	-1.213956	14.806893	7.978229
H	-1.726681	14.620628	7.032559
H	-1.760535	15.615116	8.475382
H	-1.266988	13.911747	8.598764
C	4.487387	16.221184	8.281456
C	5.571418	15.726446	7.535127
C	6.857842	16.141499	7.886660
H	7.708082	15.767850	7.320595
C	7.070396	17.024640	8.934173
H	8.079055	17.341120	9.185640
C	5.987990	17.506640	9.657364
H	6.158099	18.204797	10.473636
C	4.684934	17.114775	9.352548
C	5.398331	14.763782	6.368298
H	4.327742	14.597242	6.216736
C	5.956654	15.360877	5.070567
H	5.525007	16.344411	4.864082
H	5.723667	14.705304	4.223961
H	7.046299	15.471267	5.121347
C	6.055915	13.406774	6.651722
H	5.648064	12.929604	7.549111
H	7.137171	13.513025	6.795674
H	5.897234	12.725097	5.809454
C	3.527470	17.683408	10.164651
H	2.616699	17.137047	9.894755
C	3.732690	17.502783	11.673229
H	3.913317	16.454571	11.935029
H	2.846011	17.841213	12.219478
H	4.584649	18.086959	12.037280

C	3.299016	19.161212	9.821948
H	3.104328	19.298816	8.752183
H	4.179717	19.761162	10.077343
H	2.444235	19.559234	10.381395
C	-0.618604	16.985245	6.367708
C	-1.121986	18.129152	7.015939
C	-2.185792	18.812600	6.426088
H	-2.585028	19.698462	6.914731
C	-2.736808	18.387429	5.226547
H	-3.566228	18.931457	4.782869
C	-2.212086	17.273287	4.588407
H	-2.634139	16.953191	3.638464
C	-1.144895	16.557158	5.133451
C	-0.539397	18.666028	8.317238
H	0.278932	18.005752	8.628680
C	0.046208	20.068926	8.110573
H	0.787290	20.074399	7.304178
H	0.528885	20.423795	9.027888
H	-0.737503	20.787096	7.845328
C	-1.573797	18.666937	9.449146
H	-1.978131	17.664944	9.629336
H	-2.415845	19.328373	9.217371
H	-1.119846	19.021010	10.380978
C	-0.588130	15.369141	4.357895
H	0.261737	14.962113	4.914293
C	-1.626315	14.253352	4.178332
H	-1.995999	13.866384	5.133251
H	-1.186896	13.414502	3.628298
H	-2.492299	14.606946	3.607413
C	-0.065786	15.815989	2.985901
H	0.649824	16.637182	3.079968
H	-0.887433	16.151087	2.341742
H	0.434715	14.981646	2.481798

TS-2.log

Lowest Frequency = -254.8816cm-1

Al	0.793737	0.665487	0.495430
O	0.072858	-0.623835	-0.486811
N	2.669215	0.484928	0.309726
N	0.699815	2.539337	0.294539
C	-1.018722	-0.376616	0.249958
C	-0.594292	3.155814	0.090149
C	3.516449	1.509633	0.382666
C	3.185178	-0.827266	-0.019807
C	3.400247	-1.143160	-1.375141
C	-1.125588	3.214568	-1.210463
C	-2.521838	4.301509	0.960269
H	-3.074822	4.719373	1.798162
C	3.103710	-0.166222	-2.506740
H	2.860829	0.808538	-2.068165
C	-1.295208	3.678579	1.194818
C	-3.057758	4.379702	-0.318144
H	-4.018633	4.861022	-0.477983
C	1.881450	-0.626756	-3.313240
H	2.084144	-1.581350	-3.812186
H	1.639976	0.109638	-4.089472
H	1.006486	-0.762891	-2.669819
C	3.411014	-1.768706	0.997022
C	-0.413121	2.631879	-2.424770
H	0.539349	2.200565	-2.093633
C	-0.786410	3.573766	2.628837
H	0.116756	2.952345	2.625615
C	-2.367508	3.828369	-1.387553
H	-2.796875	3.878379	-2.385377
C	3.886834	-3.030583	0.634486
H	4.067376	-3.772595	1.408823
C	3.094641	2.847559	0.439671
H	3.876911	3.595450	0.477133
C	3.871046	-2.418061	-1.685723
H	4.035782	-2.685107	-2.726871

C	4.119412	-3.356194	-0.692737
H	4.483510	-4.345467	-0.956178
C	1.787871	3.324284	0.290082
C	3.135634	-1.476542	2.464682
H	2.761249	-0.449821	2.542972
C	-0.086894	3.713427	-3.462476
H	-1.000637	4.160259	-3.869590
H	0.470465	3.279024	-4.299064
H	0.516612	4.523053	-3.037634
C	4.999549	1.251753	0.332573
H	5.292039	0.936727	-0.674345
H	5.276352	0.440299	1.010606
H	5.558266	2.151714	0.592265
C	4.313929	0.038163	-3.426484
H	5.202470	0.361463	-2.872789
H	4.090710	0.798875	-4.181839
H	4.573135	-0.884102	-3.957398
C	-1.237449	1.504071	-3.056542
H	-1.452734	0.716181	-2.330768
H	-0.693786	1.054155	-3.893890
H	-2.191326	1.881869	-3.442247
C	1.633167	4.803670	0.043288
H	1.396782	4.963644	-1.014648
H	2.559515	5.331683	0.272154
H	0.812971	5.237648	0.615582
C	-0.407437	4.941437	3.213717
H	0.403969	5.422807	2.659687
H	-0.079318	4.830724	4.252548
H	-1.267742	5.620094	3.206241
C	-1.820991	2.894125	3.536055
H	-1.387556	2.693387	4.521372
H	-2.166924	1.946307	3.116671
H	-2.697663	3.534096	3.684300
C	2.052091	-2.415470	3.013064
H	1.754713	-2.112267	4.022607
H	2.418896	-3.446481	3.067378
H	1.158357	-2.425225	2.379872
C	4.409410	-1.565465	3.313730
H	4.188838	-1.326752	4.359485
H	5.180024	-0.869798	2.963916
H	4.835084	-2.574561	3.285393
O	-0.603103	-0.122120	3.197528
C	0.047608	0.170151	2.286394
Mn	-2.676604	-1.140470	0.193375
O	-2.361678	-1.858979	-2.630480
C	-2.468833	-1.589778	-1.508739
C	-3.645577	0.666156	0.875546
H	-3.157019	1.631998	0.844169
C	-3.609877	-0.263091	1.946921
C	-4.352732	-1.418874	1.557873
C	-4.844390	-1.195388	0.250105
H	-5.441765	-1.890873	-0.326706
C	-4.393979	0.089353	-0.180687
H	-4.595006	0.548263	-1.140522
C	-4.627538	-2.618696	2.417210
H	-4.794924	-3.510637	1.806604
H	-5.520782	-2.456211	3.030719
H	-3.790621	-2.823003	3.091454
C	-1.891949	-2.625702	0.766077
O	-1.358694	-3.567956	1.181432
H	-3.112448	-0.135369	2.899784

TS-3.log

Lowest Frequency = -337.7764cm-1

Mn	3.400321	18.870149	3.975050
Al	-0.314078	18.565508	6.147711
Al	2.550242	14.963896	5.590310
O	3.435816	16.286168	4.746205
O	0.069337	16.912392	5.891339
N	-1.231146	18.896065	7.765925

O	1.275133	19.356469	5.953978
N	1.997001	13.219776	5.012147
N	3.861991	14.343605	6.843573
N	-1.546524	19.404842	4.989229
C	1.328314	16.487507	5.539017
C	-2.524151	19.246373	7.797880
C	4.899237	16.117229	8.226502
C	1.153979	13.123128	3.843140
C	-0.556153	18.709127	9.031475
C	-2.805112	19.673784	5.343929
C	-0.232587	12.924073	3.982936
C	-0.945453	12.917649	5.330691
H	-0.188202	12.884799	6.122394
C	4.973056	15.221395	7.151660
C	-1.127943	19.790171	3.658011
C	2.303532	12.095366	5.670613
C	-3.278559	19.538931	6.656934
H	-4.322690	19.774856	6.818303
C	-1.746272	14.215285	5.511901
H	-1.101344	15.099948	5.494499
H	-2.280283	14.200308	6.471211
H	-2.497912	14.313663	4.717856
C	3.918451	13.096828	7.307522
C	3.624483	16.325684	9.025160
H	2.886812	15.587651	8.689825
C	-1.195603	16.212543	9.010935
H	-1.657246	16.509439	8.063795
C	-0.605581	21.084108	3.472266
C	-0.107499	15.184263	8.675131
H	0.607879	15.607075	7.963639
H	-0.554496	14.294429	8.216179
H	0.429652	14.866246	9.577189
C	-0.576068	17.447379	9.654597
C	3.122784	12.054904	6.805724
H	3.254789	11.084133	7.267465
C	1.750768	13.247133	2.570550

C	-0.997502	12.771725	2.823653
H	-2.069224	12.609198	2.912216
C	0.188956	21.167831	8.933526
H	-0.400447	21.127183	8.010863
C	-1.679586	17.436639	2.783703
H	-1.917573	17.283135	3.841976
C	6.120513	15.152157	6.338452
C	0.940669	13.089877	1.445246
H	1.378133	13.170429	0.455124
C	-0.419507	12.836553	1.565692
H	-1.030827	12.711195	0.675856
C	0.093674	19.810637	9.618939
C	-0.371514	22.050742	4.627788
H	-0.567564	21.516077	5.563008
C	-1.270754	18.890246	2.588239
C	-0.501061	16.513190	2.445917
H	-0.232046	16.595180	1.385110
H	-0.764932	15.469297	2.644393
H	0.381767	16.754290	3.046465
C	6.020653	16.895880	8.520502
H	5.976342	17.599441	9.348636
C	-3.222526	19.370245	9.128317
H	-3.154579	18.437921	9.694841
H	-4.272195	19.630394	8.990290
H	-2.742590	20.142418	9.737609
C	1.771866	10.770284	5.179253
H	0.698794	10.688996	5.370285
H	2.276285	9.945960	5.684684
H	1.913970	10.670774	4.099875
C	3.825106	16.122624	10.530782
H	4.215083	15.124285	10.759759
H	2.871567	16.246234	11.056769
H	4.526271	16.856222	10.944205
C	2.363585	17.180959	4.983621
C	-0.305454	21.488572	2.170760
H	0.088849	22.488078	2.001441

C	0.024627	17.327909	10.909800
H	0.013828	16.363814	11.413687
C	7.177179	16.804469	7.763535
H	8.038466	17.421573	8.004118
C	-1.334363	23.243598	4.566948
H	-1.195979	23.814326	3.641623
H	-1.154422	23.922213	5.407748
H	-2.382298	22.927332	4.611446
C	-1.870285	11.705479	5.508043
H	-2.734365	11.762767	4.837051
H	-2.257272	11.676048	6.532067
H	-1.365463	10.754219	5.309409
C	-0.968095	19.348644	1.304249
H	-1.081233	18.673167	0.459352
C	-2.281279	15.581038	9.891138
H	-2.742171	14.735064	9.369811
H	-3.075889	16.291787	10.142663
H	-1.866287	15.201586	10.831543
C	-0.504728	20.639338	1.090508
H	-0.272323	20.975157	0.083522
C	-3.781255	20.171934	4.308733
H	-3.420884	21.093059	3.842984
H	-4.759003	20.355320	4.755103
H	-3.886095	19.436985	3.505351
C	7.215374	15.946929	6.672541
H	8.109885	15.906094	6.054864
C	3.059412	17.720530	8.729650
H	3.764069	18.501731	9.038688
H	2.122970	17.877463	9.270435
H	2.870616	17.844814	7.658595
C	1.084448	22.529057	4.678862
H	1.779145	21.684753	4.727501
H	1.240109	23.156580	5.564105
H	1.336643	23.135326	3.800436
C	6.193241	14.289952	5.084511
H	5.251518	13.738403	4.989215
C	4.922094	12.742570	8.376604
H	5.940626	12.810737	7.982319
H	4.753796	11.729672	8.745101
H	4.861354	13.448647	9.209501
C	3.242521	13.533852	2.416400
H	3.515921	14.283996	3.169976
C	-2.915626	17.053031	1.962133
H	-3.783791	17.678972	2.197138
H	-3.187597	16.010962	2.162646
H	-2.724453	17.141277	0.886802
C	0.641505	18.410895	11.521414
H	1.102758	18.295002	12.498707
C	1.639728	21.476777	8.540567
H	2.283385	21.529664	9.426350
H	1.696339	22.442922	8.026204
H	2.038171	20.712305	7.867060
C	0.686435	19.635858	10.870363
H	1.194464	20.474779	11.340132
C	2.188879	19.039708	5.117115
C	-0.395967	22.290049	9.799474
H	-1.436033	22.089527	10.079774
H	-0.370478	23.239632	9.254821
H	0.177680	22.424742	10.723054
C	3.601136	14.148718	1.060872
H	3.535914	13.415079	0.248531
H	4.631730	14.517325	1.086773
H	2.948421	14.991757	0.812494
C	4.091403	12.276357	2.651201
H	3.981975	11.881851	3.665768
H	5.153503	12.500481	2.498401
H	3.809557	11.487371	1.943456
C	3.594027	17.982469	2.011578
H	3.424037	16.922239	1.872324
C	4.823298	18.572638	2.356387
H	5.770339	18.058501	2.471322
C	4.599530	19.978796	2.585463

C	3.225719	20.226359	2.353020
H	2.737362	21.189461	2.442525
C	2.594967	18.994857	2.008246
H	1.544715	18.855540	1.792907
C	6.333693	15.175889	3.838659
H	5.506646	15.892828	3.791376
H	6.332100	14.559488	2.930342
H	7.279142	15.731147	3.857526
C	7.319075	13.251922	5.157309
H	8.301157	13.732491	5.230043
H	7.321085	12.628351	4.256017
H	7.207036	12.589370	6.022961
C	5.650683	21.004813	2.893329
H	5.205750	21.881422	3.374106
H	6.153638	21.338547	1.977950
H	6.414461	20.606028	3.568218
C	4.704892	18.981152	5.186526
O	5.578582	19.139616	5.931973

TS-4.log

Lowest Frequency = -305.3521cm-1

Mn	13.144644	17.839961	4.904418
Al	8.925392	15.102259	5.108388
Al	9.118779	19.957961	4.314252
O	10.795209	19.377136	4.361443
O	8.224024	18.451246	4.207505
O	10.447691	15.932935	5.411033
C	11.256480	18.169459	4.723228
O	6.242069	16.804633	5.077877
N	8.801683	13.924761	3.626631
N	8.845611	13.771923	6.482764
O	13.163270	15.034804	5.752900
N	8.738648	21.120515	5.769433
N	8.495826	21.080577	2.926039
C	9.592210	11.609396	7.401248
H	9.466086	10.574914	7.076194
H	8.947130	11.805249	8.258916
H	10.630517	11.736242	7.727758
C	7.148248	16.091492	5.201605
O	13.123801	18.894823	7.638720
C	7.609968	22.763172	4.407271
H	7.066515	23.699574	4.442901
C	8.257982	14.301308	2.342140
C	6.039744	13.151074	2.982731
H	6.600138	12.971299	3.906848
C	8.408688	14.165424	7.800620
C	10.165015	17.210317	4.949842
C	8.845987	17.346506	4.620793
C	9.309577	12.684056	3.748295
C	9.639926	12.088259	4.966542
H	10.080483	11.100019	4.914321
C	6.603005	14.467622	9.364755
H	5.550182	14.389756	9.621848
C	8.515266	15.299293	0.167111
H	9.116755	15.853296	-0.549962
C	10.443104	15.531067	1.762429
H	10.635648	15.368367	2.828029
C	6.946450	13.901129	2.012871
C	8.071547	22.268743	5.637597
C	8.685871	20.605257	1.573417
C	9.332167	12.559027	6.259793
C	7.840944	22.227963	3.136865
C	7.033533	14.065825	8.100701
C	9.045876	15.027980	1.430470
C	9.493662	11.837296	2.512578
H	8.524504	11.496085	2.137224
H	10.103450	10.961867	2.738899
H	9.960216	12.406129	1.706055
C	9.291175	20.768173	7.062292
C	10.560774	17.036049	1.497650

H	10.560106	17.263422	0.425190
H	11.496192	17.402555	1.924601
H	9.737434	17.590938	1.957988
C	11.200445	22.452329	6.666050
H	10.539608	22.774909	5.854001
C	11.522790	14.773963	0.978103
H	11.542371	13.707287	1.223178
H	12.511962	15.182498	1.211092
H	11.366285	14.870427	-0.102756
C	8.645697	19.785704	7.840677
C	7.498351	14.966428	10.302768
H	7.144243	15.273710	11.283507
C	9.803776	21.057277	0.847067
C	7.830815	23.149491	6.839343
H	7.737783	22.577110	7.762564
H	6.932205	23.751113	6.689474
H	8.677559	23.834455	6.958703
C	8.047375	19.163483	-0.237686
H	7.364652	18.436196	-0.671165
C	7.239891	14.883680	-0.184912
H	6.847916	15.099145	-1.175934
C	4.803101	13.987044	3.337340
H	5.074747	14.941850	3.796318
H	4.163302	13.439662	4.039110
H	4.205755	14.202585	2.444155
C	9.329325	14.679813	8.731829
C	13.015119	16.139970	5.428276
C	7.311407	19.170845	7.423755
H	7.328231	19.009121	6.340281
C	10.737066	22.136149	1.386126
H	10.243807	22.614799	2.238752
C	7.776606	19.674263	1.033936
C	10.481359	21.386846	7.485709
C	10.036029	20.503081	-0.412209
H	10.902760	20.823156	-0.985243
C	6.459725	14.204911	0.740686

H	5.451938	13.897923	0.469442
C	9.171806	19.557579	-0.949277
H	9.369084	19.138122	-1.932666
C	6.494935	19.257020	1.747773
H	6.549652	19.603608	2.782427
C	6.034713	13.488510	7.104740
H	6.469556	13.587930	6.104804
C	9.244948	19.418968	9.047308
H	8.787636	18.648352	9.660213
C	10.817921	14.823108	8.447868
H	11.020597	14.484285	7.427671
C	8.842652	15.072654	9.982468
H	9.540059	15.468170	10.717337
C	7.359192	23.031549	1.955653
H	6.904018	22.396130	1.194124
H	6.643007	23.790809	2.272192
H	8.210083	23.535669	1.483405
C	6.139511	20.115906	7.728804
H	6.124786	20.384381	8.792050
H	5.189675	19.625494	7.490415
H	6.185362	21.038453	7.143657
C	6.310984	17.737642	1.795103
H	6.112574	17.320798	0.801630
H	5.458105	17.488992	2.435920
H	7.194778	17.243027	2.205362
C	11.035614	20.981048	8.699816
H	11.971719	21.421207	9.032276
C	10.430972	20.002427	9.471407
H	10.889836	19.684677	10.403732
C	13.079136	18.460268	6.561180
C	5.602722	11.787179	2.431557
H	4.991475	11.900437	1.529339
H	4.999726	11.254656	3.174926
H	6.455586	11.150942	2.175628
C	4.700516	14.240040	7.094974
H	4.159573	14.131201	8.041121

H	4.053373	13.841408	6.306910
H	4.845357	15.307616	6.901478
C	11.538738	23.693002	7.501918
H	10.659690	24.094791	8.018135
H	11.949276	24.479858	6.860269
H	12.292896	23.468031	8.263354
C	12.460909	21.874330	6.014833
H	13.173984	21.536176	6.775155
H	12.953906	22.634074	5.395215
H	12.211229	21.012899	5.389306
C	5.810613	11.992383	7.360394
H	6.744719	11.425441	7.288489
H	5.108795	11.578027	6.627467
H	5.393761	11.827181	8.360708
C	11.253425	16.289815	8.516300
H	10.718929	16.897023	7.780182
H	12.322559	16.371442	8.300419
H	11.080919	16.717692	9.511838
C	11.009737	23.229671	0.346552
H	11.606279	22.851763	-0.490636
H	11.574125	24.049507	0.802809
H	10.081488	23.640149	-0.066415
C	12.046714	21.540578	1.909688
H	11.858999	20.820975	2.712836
H	12.697650	22.330756	2.302160
H	12.591000	21.026837	1.108273
C	7.044686	17.814270	8.078786
H	7.875491	17.114158	7.932488
H	6.142102	17.369225	7.649522
H	6.873425	17.905999	9.157319
C	5.270848	19.908622	1.087470
H	5.315294	21.002335	1.112051
H	4.355272	19.601833	1.604388
H	5.182630	19.601931	0.038705
C	11.656524	13.978483	9.416977
H	11.585117	14.360388	10.442198
H	12.710266	14.012521	9.122223
H	11.339724	12.930173	9.435984
C	15.093477	18.733842	4.653872
H	15.689188	19.101395	5.481855
C	15.141803	17.416042	4.143141
C	14.192066	17.349314	3.075975
H	13.982388	16.471605	2.475849
C	13.593733	18.626709	2.932309
H	12.831299	18.891381	2.213374
C	16.067632	16.321516	4.586715
H	16.394746	16.484451	5.618006
H	16.959651	16.285339	3.950709
H	15.581440	15.342691	4.542764
C	14.145522	19.492973	3.902798
H	13.905071	20.537679	4.043255

TS-5.log

Lowest Frequency = -69.4013cm-1

Mn	3.610933	19.193349	4.520253
Al	0.161852	18.568420	6.113949
Al	2.378691	15.253201	5.561530
O	3.061201	16.291748	4.218232
O	1.496333	17.899495	7.020478
N	-1.085264	18.670491	7.582770
O	1.109447	20.160088	5.562700
N	1.537687	13.566094	5.180230
N	3.833066	14.491580	6.604571
N	-1.209375	18.839345	4.756318
C	1.779276	17.077226	5.899246
C	-2.409436	18.598825	7.482951
C	5.249658	16.111087	7.841462
C	0.617293	13.430047	4.074805
C	-0.509614	18.739541	8.910112
C	-2.521200	18.668999	4.985137

C	-0.769429	13.523763	4.288886
C	-1.374833	13.879473	5.640659
H	-0.579562	13.844295	6.393794
C	5.032284	15.293863	6.718569
C	-0.849012	19.495359	3.509979
C	1.760048	12.466467	5.924141
C	-3.078151	18.498305	6.253750
H	-4.153471	18.377152	6.298062
C	-1.916807	15.312074	5.610387
H	-1.104463	16.023459	5.426475
H	-2.401947	15.569477	6.558269
H	-2.661569	15.433377	4.813642
C	3.793172	13.283476	7.144129
C	4.353278	16.095257	9.073237
H	3.557734	15.361161	8.903623
C	-0.596806	16.170911	9.080991
H	-1.017398	16.296872	8.079563
C	-0.863953	20.909992	3.496003
C	0.714506	15.390526	8.923972
H	1.407125	15.972172	8.310218
H	0.533246	14.413900	8.454074
H	1.181045	15.204521	9.899612
C	-0.319647	17.556733	9.651061
C	2.745689	12.369991	6.903901
H	2.802886	11.430322	7.439674
C	1.140531	13.169479	2.789503
C	-1.626146	13.301425	3.207380
H	-2.701241	13.371553	3.356216
C	-0.235962	21.275539	8.619122
H	-0.624505	21.026987	7.626341
C	-0.594831	17.252549	2.268799
H	-0.887566	16.864804	3.251210
C	5.954648	15.243470	5.654802
C	0.242759	12.921543	1.750937
H	0.620816	12.703355	0.757029
C	-1.130618	12.978003	1.954222
H	-1.811920	12.785720	1.129293
C	-0.131922	19.988880	9.426218
C	-1.329531	21.739622	4.688633
H	-1.347307	21.091004	5.568666
C	-0.514261	18.767931	2.358116
C	0.754728	16.631081	1.901313
H	1.122926	17.029183	0.947128
H	0.638222	15.549656	1.785718
H	1.513062	16.807124	2.667534
C	6.382429	16.928516	7.844839
H	6.554613	17.586665	8.692505
C	-3.263343	18.645428	8.725465
H	-3.038651	17.808853	9.391154
H	-4.322864	18.623134	8.468199
H	-3.051074	19.557828	9.291236
C	0.937742	11.223249	5.677792
H	-0.118987	11.406416	5.887274
H	1.283259	10.404691	6.309908
H	1.005853	10.915427	4.630378
C	5.147146	15.651264	10.312105
H	5.687114	14.712209	10.150473
H	4.472942	15.518919	11.165598
H	5.886469	16.409317	10.593701
C	2.751088	17.428421	4.930286
C	-0.496452	21.568277	2.324893
H	-0.504012	22.655092	2.299843
C	0.191672	17.663865	10.943167
H	0.340918	16.760685	11.531392
C	7.281606	16.924177	6.789808
H	8.154684	17.571543	6.814686
C	-2.761208	22.249545	4.468845
H	-2.814916	22.887701	3.579184
H	-3.091925	22.841634	5.329377
H	-3.472323	21.427881	4.335216
C	-2.482313	12.910962	6.075672
H	-3.366863	12.998963	5.435403

H	-2.797697	13.139710	7.099581
H	-2.159360	11.865223	6.047339
C	-0.148399	19.477218	1.208025
H	0.114497	18.924475	0.309168
C	-1.600269	15.367186	9.917079
H	-1.801607	14.402057	9.436915
H	-2.557620	15.884865	10.038847
H	-1.208412	15.158682	10.918917
C	-0.128591	20.861409	1.186204
H	0.156995	21.391534	0.281303
C	-3.494166	18.661374	3.829660
H	-3.270369	19.431200	3.089638
H	-4.518026	18.789581	4.183965
H	-3.424139	17.692469	3.322787
C	7.074945	16.071414	5.712806
H	7.802732	16.041137	4.904471
C	3.685712	17.448884	9.338975
H	4.437546	18.228931	9.502064
H	3.064777	17.386072	10.241395
H	3.041464	17.758800	8.509799
C	-0.394519	22.910955	5.006532
H	0.634727	22.567819	5.138347
H	-0.715871	23.400022	5.934123
H	-0.411398	23.672225	4.218610
C	5.818050	14.252924	4.505121
H	4.831098	13.784936	4.573293
C	4.896725	12.802765	8.053395
H	5.856106	13.263905	7.814462
H	4.990047	11.716665	8.000223
H	4.648273	13.071256	9.086209
C	2.643292	13.184887	2.520872
H	3.068097	13.989576	3.132422
C	-1.654389	16.817523	1.243807
H	-2.623988	17.297046	1.411122
H	-1.791423	15.731428	1.287528
H	-1.337649	17.071808	0.225665

C	0.532907	18.896328	11.485984
H	0.930948	18.957876	12.495403
C	1.146100	21.910998	8.423202
H	1.566830	22.246048	9.378124
H	1.074222	22.782619	7.763259
H	1.846304	21.199356	7.975335
C	0.383461	20.043973	10.723661
H	0.675807	21.006126	11.138373
C	2.200402	19.873005	5.083442
C	-1.216190	22.269743	9.252292
H	-2.218413	21.838965	9.355648
H	-1.298529	23.171554	8.634985
H	-0.881330	22.578311	10.249101
C	2.986069	13.525973	1.067381
H	2.723290	12.712485	0.381041
H	4.063695	13.691032	0.974734
H	2.478162	14.437208	0.736179
C	3.323086	11.866850	2.916743
H	3.246879	11.662030	3.988812
H	4.389858	11.902172	2.665483
H	2.879072	11.025215	2.371394
C	4.120400	18.419045	2.569129
H	3.698646	17.484359	2.226591
C	5.303155	18.550182	3.343775
H	5.965160	17.750165	3.648817
C	5.469441	19.932850	3.667401
C	4.374542	20.632816	3.108797
H	4.197355	21.697319	3.203719
C	3.535769	19.696776	2.426146
H	2.608182	19.923645	1.914537
C	5.906615	14.919379	3.130491
H	5.099178	15.648064	3.013781
H	5.818465	14.162999	2.341233
H	6.867432	15.426339	2.983668
C	6.859479	13.133778	4.644018
H	7.877440	13.533213	4.569306

H	6.732039	12.388017	3.850798
H	6.773818	12.619766	5.607873
C	6.628042	20.528317	4.413116
H	6.335011	21.443876	4.935196
H	7.441722	20.777511	3.722769
H	7.019451	19.825453	5.155384
C	4.322462	19.306636	6.147354
O	4.823381	19.507718	7.170146

TS-6.log

Lowest Frequency = -209.7667cm-1

Mn	3.536850	19.205855	4.112459
Al	0.253383	18.224599	5.975817
Al	2.343664	15.350841	5.512673
O	3.574998	16.372352	4.434679
O	1.645944	17.232780	6.659140
N	-0.876789	18.333690	7.565378
O	1.292833	19.901722	5.774098
N	1.576197	13.619385	5.164644
N	3.638392	14.700788	6.797330
N	-1.216531	18.920091	4.815332
C	1.470335	17.003937	5.096211
C	-2.215118	18.403958	7.554179
C	5.052647	16.341335	8.040985
C	0.732307	13.460804	4.002490
C	-0.252443	18.325053	8.872464
C	-2.509493	18.826589	5.110674
C	-0.668579	13.505624	4.127694
C	-1.370933	13.828292	5.440628
H	-0.625819	13.800946	6.242655
C	4.885855	15.424817	6.990043
C	-0.896445	19.576281	3.566182
C	1.744811	12.550578	5.956731
C	-2.982216	18.513069	6.393876
H	-4.057315	18.482965	6.519193
C	-1.941388	15.250234	5.395902
H	-1.138848	15.970968	5.211299
H	-2.443098	15.506728	6.335776
H	-2.676840	15.352707	4.587861
C	3.545656	13.517497	7.402949
C	3.945602	16.735348	9.007771
H	3.044947	16.173081	8.747397
C	-0.628462	15.781495	9.076248
H	-1.158720	15.961449	8.135290
C	-0.770623	20.983359	3.579284
C	0.558705	14.866657	8.763975
H	1.211772	15.356785	8.038191
H	0.219692	13.902795	8.365686
H	1.146456	14.661190	9.667819
C	-0.150684	17.127560	9.608765
C	2.596673	12.538986	7.061419
H	2.614960	11.627515	7.645611
C	1.344108	13.247958	2.750239
C	-1.443684	13.274359	2.988944
H	-2.528040	13.305169	3.068345
C	0.115247	20.860131	8.653742
H	-0.210749	20.640613	7.634989
C	-0.865606	17.337761	2.290560
H	-1.052265	16.958775	3.298715
C	5.946735	15.127013	6.108024
C	0.526393	12.994475	1.648121
H	0.977954	12.811249	0.677724
C	-0.857568	12.997493	1.762835
H	-1.477743	12.801774	0.891927
C	0.188931	19.545321	9.418384
C	-0.945876	21.808025	4.850155
H	-0.901712	21.123752	5.700264
C	-0.775487	18.854239	2.365519
C	0.463613	16.738457	1.819119
H	0.717121	17.084728	0.808745

H	0.396847	15.646499	1.796283
H	1.272912	17.019788	2.496385
C	6.309216	16.931146	8.205074
H	6.452010	17.646570	9.011079
C	-2.984700	18.380492	8.854848
H	-2.858697	17.424836	9.368705
H	-4.047452	18.541659	8.671194
H	-2.620408	19.153723	9.536620
C	1.003211	11.268986	5.660731
H	-0.069116	11.395205	5.833189
H	1.364906	10.462438	6.299462
H	1.125868	10.978716	4.613778
C	4.308935	16.425048	10.467050
H	4.596124	15.379537	10.623997
H	3.451242	16.642753	11.113557
H	5.144923	17.046257	10.808522
C	2.836275	17.434995	4.541059
C	-0.541289	21.644865	2.372565
H	-0.452883	22.728673	2.368320
C	0.396394	17.181037	10.891769
H	0.485291	16.263291	11.469386
C	7.366081	16.639714	7.360468
H	8.332833	17.113373	7.509127
C	-2.318712	22.494002	4.883006
H	-2.443088	23.166364	4.025980
H	-2.424264	23.089591	5.796842
H	-3.137945	21.768117	4.863803
C	-2.479882	12.828360	5.791848
H	-3.324675	12.906252	5.098782
H	-2.864243	13.034452	6.796795
H	-2.131720	11.790430	5.768925
C	-0.563309	19.565995	1.180888
H	-0.481606	19.019298	0.243914
C	-1.590484	15.068379	10.036869
H	-1.998173	14.170099	9.559373
H	-2.431761	15.698684	10.342118
H	-1.076113	14.746435	10.949008
C	-0.448495	20.947551	1.175210
H	-0.283622	21.480212	0.242256
C	-3.565091	19.074471	4.056766
H	-3.365927	19.974708	3.472822
H	-4.553430	19.153223	4.511937
H	-3.574133	18.233869	3.355544
C	7.177825	15.746348	6.314874
H	8.006504	15.519108	5.647970
C	3.606258	18.223439	8.866450
H	4.491048	18.850504	9.027144
H	2.850151	18.502720	9.605985
H	3.197254	18.419206	7.872763
C	0.167314	22.844426	5.036623
H	1.156615	22.380522	4.989123
H	0.064353	23.328611	6.014963
H	0.119779	23.634489	4.278507
C	5.821873	14.125438	4.965133
H	4.772546	13.827300	4.880584
C	4.519716	13.149083	8.495176
H	5.553777	13.238448	8.153825
H	4.343671	12.130579	8.843141
H	4.405467	13.836489	9.337626
C	2.859358	13.295473	2.583823
H	3.246793	14.038424	3.290021
C	-1.999880	16.861637	1.372903
H	-2.970685	17.286260	1.648460
H	-2.078607	15.769604	1.417383
H	-1.808688	17.136758	0.329178
C	0.807587	18.381900	11.454556
H	1.213726	18.403345	12.462583
C	1.490885	21.529109	8.554299
H	1.851999	21.855613	9.536423
H	1.435259	22.412812	7.908884
H	2.230705	20.845022	8.128277
C	0.700403	19.551887	10.717829

H	1.029070	20.492694	11.153701
C	2.264397	19.761822	5.058849
C	-0.917341	21.814461	9.265651
H	-1.919843	21.372563	9.275846
H	-0.967255	22.743791	8.686271
H	-0.655323	22.077055	10.297173
C	3.292292	13.770205	1.193570
H	3.099611	13.017077	0.420945
H	4.368574	13.969208	1.191750
H	2.776837	14.693946	0.909266
C	3.512388	11.948153	2.918064
H	3.355731	11.664755	3.964107
H	4.594296	11.994823	2.747729
H	3.105137	11.153708	2.281402
C	3.794315	18.455095	2.098800
H	3.510086	17.449739	1.813875
C	5.055966	18.833205	2.614498
H	5.905569	18.179352	2.770102
C	5.014791	20.238049	2.906744
C	3.718936	20.695864	2.570015
H	3.361642	21.711267	2.694390
C	2.954791	19.598163	2.075455
H	1.925567	19.634771	1.746061
C	6.231454	14.740145	3.622024
H	5.627640	15.628382	3.414311
H	6.084305	14.013165	2.813648
H	7.290393	15.021913	3.614105
C	6.629077	12.851790	5.251019
H	7.695356	13.078273	5.364718
H	6.523820	12.136877	4.426804
H	6.292537	12.355058	6.167510
C	6.161115	21.077678	3.390389
H	5.799181	21.976214	3.898511
H	6.789248	21.393639	2.549921
H	6.793208	20.525863	4.092332
C	4.680216	19.184853	5.480136
O	5.479656	19.265796	6.310071

TS-7.log

Lowest Frequency = -336.8510cm-1

Mn	1.966807	1.887226	-1.403744
Al	-1.700831	1.484655	0.637558
Al	0.802279	-2.153913	0.132321
O	1.422643	-0.723186	-0.709277
O	-0.648992	-1.337899	0.690551
N	-2.722872	1.728415	2.228080
O	-0.341457	2.661336	0.248072
N	0.292475	-3.871913	-0.514538
N	2.245442	-2.752784	1.216190
N	-3.042723	2.123686	-0.573144
C	-0.495642	0.012585	0.373089
C	-4.023914	2.046851	2.237863
C	3.283706	-0.897469	2.478942
C	-0.577914	-3.983823	-1.661661
C	-2.070391	1.552022	3.505797
C	-4.320126	2.309167	-0.237808
C	-1.967627	-4.121614	-1.490342
C	-2.655167	-4.006849	-0.135224
H	-1.882729	-3.976638	0.641029
C	3.362575	-1.866743	1.468495
C	-2.642936	2.518518	-1.908299
C	0.620212	-4.996589	0.136472
C	-4.789336	2.228954	1.081348
H	-5.844021	2.424299	1.229346
C	-3.428635	-2.683008	-0.054511
H	-2.738592	-1.841595	-0.161039
H	-3.929440	-2.593205	0.917226
H	-4.198101	-2.631044	-0.835077
C	2.339728	-4.003140	1.665710
C	2.003708	-0.632311	3.256435

H	1.170071	-1.092518	2.713019
C	-2.540309	-0.968027	3.329907
H	-2.416320	-0.796029	2.256414
C	-2.271247	3.861077	-2.125776
C	-1.742859	-2.237943	3.640128
H	-0.668258	-2.075605	3.515915
H	-2.042370	-3.029797	2.944600
H	-1.927657	-2.606697	4.656370
C	-2.010694	0.259458	4.066064
C	1.502172	-5.039803	1.222241
H	1.654526	-6.009515	1.679871
C	0.001593	-3.943449	-2.945972
C	-2.754413	-4.312777	-2.629477
H	-3.830130	-4.429439	-2.517690
C	-1.504276	4.056987	3.535153
H	-2.125370	4.035068	2.632253
C	-2.897860	0.099934	-2.734415
H	-3.129686	-0.053922	-1.674612
C	4.514982	-2.002686	0.672969
C	-0.826818	-4.144118	-4.050444
H	-0.401163	-4.128632	-5.049214
C	-2.192409	-4.346812	-3.896745
H	-2.821117	-4.504771	-4.769034
C	-1.531991	2.670411	4.165452
C	-2.189390	4.892116	-1.005015
H	-2.291524	4.366511	-0.050860
C	-2.645454	1.582954	-2.955827
C	-1.630244	-0.702991	-3.052997
H	-1.363888	-0.608486	-4.113433
H	-1.796871	-1.762866	-2.845367
H	-0.781382	-0.358419	-2.453313
C	4.425016	-0.142942	2.755967
H	4.389333	0.606879	3.541307
C	-4.725422	2.289135	3.552945
H	-4.455132	1.554523	4.312823
H	-5.807584	2.288345	3.414656
H	-4.429737	3.272054	3.936910
C	0.037692	-6.314614	-0.313008
H	-1.036822	-6.346748	-0.114626
H	0.514236	-7.142904	0.212249
H	0.166628	-6.451135	-1.390126
C	2.053676	-1.259563	4.654787
H	2.154825	-2.350060	4.607696
H	1.137813	-1.028903	5.210250
H	2.903402	-0.866689	5.225607
C	0.620185	0.342074	-0.344091
C	-1.977597	4.258833	-3.430575
H	-1.699478	5.292779	-3.620637
C	-1.463385	0.130994	5.343745
H	-1.422574	-0.849868	5.809560
C	5.592187	-0.307435	2.026795
H	6.465231	0.298551	2.252344
C	-3.326460	5.918501	-1.097878
H	-3.296813	6.452746	-2.054513
H	-3.235390	6.661107	-0.297731
H	-4.312166	5.450092	-1.005436
C	-3.592953	-5.184074	0.161649
H	-4.452649	-5.191916	-0.517515
H	-3.984683	-5.101759	1.181138
H	-3.094760	-6.155007	0.070354
C	-2.353653	2.034047	-4.245450
H	-2.362079	1.324707	-5.069954
C	-4.029449	-1.208219	3.617818
H	-4.353957	-2.154286	3.169758
H	-4.666264	-0.418759	3.207701
H	-4.213221	-1.271313	4.697385
C	-2.036570	3.361961	-4.489022
H	-1.812121	3.693991	-5.499143
C	-5.331893	2.647775	-1.304822
H	-5.054714	3.558690	-1.841017
H	-6.324464	2.775252	-0.871440
H	-5.367907	1.846727	-2.049301

C	5.623938	-1.214252	0.976943
H	6.523336	-1.301822	0.371749
C	1.698633	0.867175	3.337280
H	2.427067	1.400703	3.958466
H	0.712385	1.021170	3.782248
H	1.702754	1.318555	2.340528
C	-0.828203	5.598307	-0.986729
H	-0.009885	4.874012	-0.949187
H	-0.755598	6.244722	-0.104637
H	-0.693701	6.234515	-1.869427
C	4.555240	-2.896460	-0.559804
H	3.667184	-3.539124	-0.557147
C	3.437466	-4.381095	2.628581
H	4.377960	-4.517845	2.084463
H	3.194385	-5.318354	3.132195
H	3.609177	-3.596949	3.368785
C	1.491671	-3.679110	-3.134118
H	1.803539	-2.982745	-2.345815
C	-4.082427	-0.430706	-3.550299
H	-5.015543	0.094769	-3.318796
H	-4.231160	-1.496233	-3.340961
H	-3.902670	-0.329518	-4.626562
C	-0.958498	1.230319	6.028279
H	-0.533580	1.104099	7.020660
C	-0.076756	4.422220	3.104733
H	0.594149	4.451342	3.971269
H	-0.062058	5.410454	2.631301
H	0.320706	3.701183	2.384819
C	-0.976459	2.482711	5.433179
H	-0.551316	3.333418	5.960203
C	0.638756	2.230964	-0.422685
C	-2.079992	5.132640	4.464024
H	-3.087282	4.879305	4.812579
H	-2.133011	6.092820	3.940680
H	-1.450820	5.277909	5.348914
C	1.810209	-2.993303	-4.465488

H	1.696508	-3.675467	-5.315897
H	2.848255	-2.645637	-4.464829
H	1.163976	-2.125205	-4.633441
C	2.322665	-4.960068	-2.982458
H	2.230666	-5.394398	-1.981848
H	3.384676	-4.750125	-3.154759
H	2.005566	-5.712922	-3.713899
C	2.194809	0.900542	-3.316713
H	1.948594	-0.148657	-3.431227
C	3.447984	1.409372	-2.924675
H	4.347928	0.833439	-2.746070
C	3.321731	2.836114	-2.764648
C	1.985314	3.177028	-3.081503
H	1.568661	4.177085	-3.050320
C	1.278952	1.983704	-3.416376
H	0.233540	1.917838	-3.687279
C	4.483959	-2.015280	-1.815426
H	3.602403	-1.364231	-1.782381
H	4.448422	-2.631843	-2.722241
H	5.371649	-1.374707	-1.882775
C	5.779978	-3.815860	-0.602117
H	6.710995	-3.244960	-0.686079
H	5.724034	-4.479807	-1.471946
H	5.853424	-4.441164	0.294561
C	4.431794	3.792334	-2.439741
H	4.032072	4.721762	-2.023037
H	5.007429	4.043423	-3.338460
H	5.123707	3.368748	-1.705277
C	3.163324	2.070381	-0.100108
O	3.985199	2.304407	0.687261

2-W.log

Lowest Frequency = 17.9966cm-1

W	1.960203	-1.856380	2.147237

Al	-0.896490	0.806863	-0.156847
O	-0.128262	0.376314	1.409772
O	-0.593932	-3.805360	2.392511
N	-2.809307	0.444036	-0.048184
O	4.479485	0.136697	2.251332
O	0.779593	0.340545	4.355589
N	-1.125476	2.691124	-0.531822
C	0.753461	-0.528079	0.754128
O	2.886504	-3.300412	-0.456201
O	3.430802	-3.670789	4.341379
C	3.575904	-0.569295	2.146849
C	0.293102	-3.080689	2.290400
C	2.889141	-2.999753	3.571001
C	1.134507	-0.422176	3.589180
C	2.572301	-2.820971	0.639046
C	-0.066503	3.480790	-1.114847
C	-3.744718	1.381154	0.059086
C	-3.214580	-0.940121	0.006808
C	-3.448473	-1.556086	1.251784
C	1.042893	3.861221	-0.346269
C	0.752060	4.754750	-2.988776
H	0.652831	5.108212	-4.011180
C	-3.454682	-0.789390	2.570051
H	-3.387067	0.279943	2.344981
C	-0.214704	3.906503	-2.455121
C	1.845991	5.163959	-2.231331
H	2.585148	5.834525	-2.662532
C	-2.237721	-1.134601	3.429842
H	-2.231691	-2.198173	3.693605
H	-2.250292	-0.551316	4.358020
H	-1.330130	-0.885988	2.874230
C	-3.341622	-1.650977	-1.197248
C	1.233696	3.416947	1.095885
H	0.413923	2.747067	1.368731
C	-1.399408	3.445765	-3.301467
H	-2.260154	3.310641	-2.638161
C	1.992157	4.707672	-0.931599
H	2.851089	5.023500	-0.343066
C	-3.620362	-3.016964	-1.137239
H	-3.695270	-3.586913	-2.061275
C	-3.459462	2.755501	0.092831
H	-4.294603	3.416728	0.288694
C	-3.719540	-2.925195	1.260739
H	-3.877097	-3.428143	2.211774
C	-3.791792	-3.656214	0.082584
H	-3.997257	-4.723285	0.116980
C	-2.239334	3.363905	-0.203139
C	-3.194626	-0.966924	-2.546926
H	-3.159871	0.115733	-2.377618
C	1.212685	4.610729	2.057936
H	2.040453	5.301754	1.859365
H	1.309161	4.263439	3.091932
H	0.278709	5.178846	1.981206
C	-5.198226	0.983881	0.136028
H	-5.841069	1.864462	0.109582
H	-5.407343	0.422240	1.049873
H	-5.452934	0.329800	-0.703827
C	-4.752155	-1.024957	3.355724
H	-5.648371	-0.830308	2.755921
H	-4.782652	-0.372040	4.234298
H	-4.817546	-2.057902	3.714507
C	2.520713	2.602630	1.255069
H	2.511091	1.726372	0.598768
H	2.612597	2.250588	2.288189
H	3.413518	3.197798	1.024862
C	-2.232729	4.875813	-0.207672
H	-2.223231	5.265376	-1.230710
H	-1.339562	5.264042	0.288242
H	-3.120688	5.257709	0.298106
C	-1.819602	4.457610	-4.370645
H	-1.984785	5.453684	-3.945788
H	-2.750899	4.131852	-4.845141

H	-1.069421	4.549474	-5.163877
C	-1.116541	2.080865	-3.943085
H	-2.016934	1.691371	-4.433771
H	-0.783007	1.342747	-3.205329
H	-0.329093	2.166497	-4.701203
C	-1.890948	-1.385715	-3.225496
H	-1.799052	-0.904840	-4.205630
H	-1.877342	-2.467665	-3.381307
H	-1.011105	-1.128723	-2.625508
C	-4.394848	-1.243625	-3.460171
H	-4.310796	-0.656382	-4.381307
H	-5.342954	-0.984464	-2.975966
H	-4.444052	-2.299548	-3.749029
O	1.253645	-1.129437	-1.594732
C	0.582967	-0.421823	-0.596776
Al	2.481705	-2.242961	-1.957578
N	4.282470	-1.766228	-2.444620
N	2.150701	-3.433550	-3.389547
C	4.986326	-2.474377	-3.317613
C	4.495274	-3.636230	-3.945834
H	5.212501	-4.192058	-4.538685
C	3.172696	-4.055073	-4.014838
C	6.381482	-2.067937	-3.722582
H	6.660912	-1.083496	-3.348949
H	6.447254	-2.071548	-4.813794
H	7.101112	-2.802974	-3.348674
C	2.884771	-5.238006	-4.907614
H	2.381063	-4.921329	-5.826224
H	2.218987	-5.947333	-4.408333
H	3.813486	-5.742801	-5.176518
C	4.886658	-0.626295	-1.789396
C	4.489866	0.656000	-2.202869
C	4.987520	1.759557	-1.508832
H	4.670248	2.758681	-1.799889
C	5.882459	1.597469	-0.461705
H	6.257179	2.464059	0.076368
C	6.313447	0.325353	-0.112748
H	7.032579	0.209849	0.694339
C	5.831714	-0.813494	-0.759413
C	3.623442	0.871156	-3.433197
H	3.292129	-0.106251	-3.799844
C	2.379266	1.698463	-3.127566
H	1.748835	1.196026	-2.386919
H	1.793659	1.844757	-4.041232
H	2.639705	2.689034	-2.745090
C	4.454932	1.507045	-4.555889
H	5.338723	0.904303	-4.794692
H	4.800096	2.507344	-4.270987
H	3.853674	1.607412	-5.466728
C	6.414076	-2.172255	-0.374860
H	5.958587	-2.940044	-1.009549
C	7.933892	-2.192774	-0.608545
H	8.209875	-1.852659	-1.610851
H	8.325864	-3.205865	-0.468039
H	8.444451	-1.541684	0.109446
C	6.127424	-2.564725	1.078892
H	5.058384	-2.678640	1.269634
H	6.517411	-1.816074	1.777245
H	6.612628	-3.519780	1.307903
C	0.830841	-3.768137	-3.879599
C	0.041978	-4.703027	-3.194551
C	-1.186633	-5.068384	-3.753804
H	-1.812127	-5.791841	-3.236496
C	-1.606866	-4.541003	-4.965353
H	-2.557263	-4.848562	-5.394123
C	-0.812739	-3.612269	-5.629627
H	-1.155324	-3.199472	-6.574329
C	0.409961	-3.202114	-5.101857
C	0.492491	-5.313481	-1.878425
H	1.563609	-5.115255	-1.765332
C	-0.231693	-4.646107	-0.704480
H	-0.102877	-3.556052	-0.700241

H	0.142143	-5.040993	0.245027
H	-1.309816	-4.838379	-0.748843
C	0.309543	-6.834187	-1.836647
H	0.796825	-7.325584	-2.686182
H	-0.748536	-7.117866	-1.846586
H	0.744678	-7.236507	-0.916316
C	1.257016	-2.161110	-5.827318
H	2.306050	-2.330250	-5.560875
C	1.165754	-2.257431	-7.352807
H	1.371729	-3.272274	-7.710340
H	1.893609	-1.581006	-7.812367
H	0.177074	-1.964357	-7.722333
C	0.894387	-0.746317	-5.355105
H	0.930200	-0.657572	-4.263095
H	-0.121067	-0.484355	-5.675230
H	1.583341	-0.009543	-5.786719

3-W.log

Lowest Frequency = 12.5233cm-1

W	17.451025	5.053474	14.893182
Al	16.065951	2.432220	11.052754
Al	20.479176	1.587528	12.274698
O	17.556088	1.517155	10.914338
O	16.364321	3.474437	12.453500
O	19.837375	2.925328	13.289227
O	14.310939	4.462890	15.127432
N	21.153612	0.113700	13.261042
N	14.452087	1.477926	11.288671
N	21.972982	1.812485	11.125272
O	20.604554	5.666099	14.648021
O	16.686350	7.515814	12.987852
N	15.551613	3.449369	9.544533
C	17.465634	3.653208	13.169294
O	18.055941	2.698524	16.998781
C	18.573980	1.866860	11.721679
C	18.570015	2.833958	12.703557
O	17.349959	7.065710	17.374241
C	19.482641	5.410857	14.688292
C	15.445441	4.669533	15.047191
C	20.327428	-0.306997	14.371316
C	22.330618	-0.468568	13.040375
C	17.391294	6.336158	16.475233
C	16.980262	6.616127	13.651309
C	17.863019	3.520298	16.212916
C	16.466141	3.847003	8.495121
C	13.314278	2.139130	11.033023
C	14.357987	0.162712	11.890388
C	14.261359	0.025724	13.284284
C	23.053548	1.021190	11.150855
C	17.361450	4.913270	8.695810
C	17.328227	3.516421	6.268428
H	17.317159	2.980270	5.321919
C	14.382762	1.195883	14.246846
H	14.397634	2.129157	13.674298
C	19.262902	-1.194807	14.133441
C	16.442006	3.137581	7.277095
C	23.218208	-0.048097	12.039250
H	24.142480	-0.605736	11.952687
C	18.224124	4.559774	6.453138
H	18.909307	4.841133	5.657159
C	20.562693	0.242987	15.645826
C	15.709126	1.098130	15.010358
H	15.768836	0.159435	15.573746
H	15.796642	1.921787	15.723259
H	16.576607	1.133947	14.340819
C	14.345718	-0.964836	11.041878
C	17.406351	5.746702	9.968829
H	16.693057	5.331977	10.689604
C	15.501433	1.968824	7.011556
H	14.856030	1.834493	7.886448

C	18.235870	5.244272	7.657869
H	18.938687	6.060802	7.802613
C	14.147389	-2.219535	11.616473
H	14.119222	-3.101064	10.983715
C	21.904033	2.847822	10.116206
C	13.261257	3.325750	10.283248
H	12.288810	3.794145	10.199818
C	14.082684	-1.257101	13.811611
H	13.999880	-1.377094	14.889038
C	19.054555	-1.887362	12.792591
H	19.777644	-1.476266	12.079016
C	14.001638	-2.368582	12.990261
H	13.844351	-3.355595	13.417335
C	22.225957	4.175678	10.468839
C	14.283634	3.874612	9.494160
C	22.765385	-1.613550	13.916666
H	23.046971	-1.235586	14.905146
H	23.625007	-2.127729	13.485117
H	21.951263	-2.325909	14.067930
C	14.549368	-0.839135	9.536008
H	15.239540	-0.001946	9.373358
C	21.745895	1.156413	15.946055
H	22.389678	1.186255	15.059567
C	21.105366	1.089745	8.410176
H	21.366173	0.408962	9.227928
C	16.997437	7.199433	9.686733
H	17.697764	7.674900	8.990531
H	16.993993	7.775959	10.615300
H	15.996858	7.266787	9.245945
C	18.404357	-1.489131	15.194403
H	17.568400	-2.165967	15.029936
C	19.671544	-0.078195	16.669571
H	19.814847	0.357401	17.654989
C	12.006115	1.598434	11.550414
H	11.877753	0.547710	11.278345
H	11.167637	2.176074	11.160667
H	11.992835	1.650934	12.643362
C	19.589996	0.996285	8.193569
H	19.028350	1.240515	9.100889
H	19.314416	-0.019215	7.884095
H	19.268642	1.687926	7.405067
C	22.192334	5.138069	9.457716
H	22.447073	6.166527	9.691052
C	13.212221	1.271430	15.235450
H	12.240107	1.257363	14.730420
H	13.278685	2.197632	15.813253
H	13.228065	0.433350	15.941275
C	18.800676	5.703780	10.607467
H	19.140325	4.676424	10.773264
H	18.791940	6.217695	11.574439
H	19.542704	6.197873	9.969770
C	21.460705	3.513513	7.847754
H	21.138190	3.272035	6.837821
C	21.505843	2.504370	8.811171
C	22.590656	4.569359	11.900504
H	21.872252	4.074652	12.567722
C	17.654044	-1.649325	12.221870
H	16.873203	-2.037706	12.885017
H	17.552413	-2.164268	11.259197
H	17.463515	-0.586819	12.051559
C	18.595136	-0.926465	16.448244
H	17.907217	-1.152793	17.258686
C	24.167603	1.251764	10.159590
H	23.865501	0.892443	9.170846
H	25.065166	0.710765	10.461953
H	24.400939	2.313763	10.059509
C	19.328644	-3.393206	12.915324
H	18.616648	-3.863958	13.602510
H	20.336463	-3.599619	13.291058
H	19.225865	-3.881676	11.940318
C	13.900179	4.955083	8.518061
H	14.603001	5.790028	8.570684

H	12.891320	5.316307	8.719940
H	13.935632	4.572515	7.493784
C	14.594211	2.219278	5.799726
H	14.020290	3.146268	5.897688
H	13.882046	1.394908	5.683182
H	15.175351	2.287533	4.873558
C	22.454628	6.072474	12.157173
H	23.217675	6.647192	11.618310
H	22.580560	6.271424	13.223645
H	21.467083	6.446115	11.867351
C	16.294755	0.671968	6.814555
H	15.616657	-0.159896	6.594596
H	16.878580	0.420292	7.706321
H	16.992462	0.760508	5.974275
C	22.588805	0.617830	17.109910
H	22.029880	0.649233	18.051336
H	23.486079	1.231484	17.241668
H	22.904390	-0.419574	16.950404
C	21.846536	0.610200	7.154739
H	21.531725	1.170461	6.267706
H	21.626344	-0.445999	6.967954
H	22.932638	0.720665	7.243442
C	21.817024	4.815207	8.160085
H	21.792351	5.586852	7.394178
C	21.295262	2.594257	16.226935
H	20.710124	2.982571	15.388705
H	22.166435	3.241454	16.379789
H	20.678200	2.638969	17.131146
C	24.007062	4.123241	12.292845
H	24.119555	3.036028	12.312679
H	24.247597	4.493166	13.294982
H	24.749324	4.534934	11.597792
C	15.196379	-2.086757	8.926205
H	15.486579	-1.891662	7.890243
H	14.504751	-2.936345	8.910816
H	16.092573	-2.385045	9.478891
C	13.242473	-0.517225	8.797569
H	12.828444	0.452905	9.088645
H	12.487797	-1.286614	8.998910
H	13.412040	-0.490008	7.714498

4-W.log

Lowest Frequency = 12.3783cm-1

W	3.241032	4.855354	7.154592
Al	1.774322	9.182707	5.503079
Al	6.988674	7.864194	5.101126
O	2.101100	7.511322	6.012133
N	7.786752	7.073011	3.578830
O	3.380901	9.713332	5.097963
O	5.621722	6.947545	5.773376
O	3.284746	2.205584	8.932149
N	8.518302	7.956337	6.201922
N	0.907101	10.248971	6.780722
N	0.610086	9.418265	4.053360
C	0.342526	9.363973	8.998229
C	-0.549293	12.105487	7.461163
H	0.256389	12.505092	8.081618
H	-1.239226	11.581375	8.131135
H	-1.086771	12.927273	6.986452
C	10.679327	7.105165	7.005018
H	10.969566	8.114584	7.307374
H	11.547644	6.585575	6.598483
H	10.349339	6.579036	7.906368
C	7.236083	7.299870	2.258984
C	9.673753	6.308096	4.874631
H	10.534400	5.650763	4.857080
O	3.243439	2.981665	4.552097
C	8.544810	6.496714	10.099899
H	8.334557	7.011915	11.043727
H	9.625209	6.555942	9.929030

H	8.277861	5.443717	10.234887
C	6.242099	7.090555	9.279368
H	5.922406	6.087988	9.581854
H	5.644557	7.389403	8.411734
H	6.019119	7.777151	10.104239
O	2.991490	6.582921	9.861152
C	8.524283	8.910593	7.290519
C	4.429645	7.607061	5.695744
O	5.907545	10.632511	4.515997
C	4.431166	8.910821	5.236373
C	0.306767	6.606315	1.634380
H	-0.177538	5.638468	1.553820
C	5.783633	9.461927	4.857711
C	8.898382	6.340236	3.704579
C	7.737405	7.096168	8.941336
H	7.873394	6.455111	8.064547
C	3.849981	5.840846	2.654033
H	3.339860	5.226150	3.398578
H	3.414018	5.596547	1.679238
H	3.618073	6.890890	2.863974
C	10.624771	10.461718	5.234427
H	10.848835	10.814333	4.220737
H	10.877529	9.397142	5.276980
H	11.282929	10.995832	5.929759
C	3.961969	9.999606	1.882525
H	4.200380	9.196414	2.584086
H	4.564284	10.868191	2.164862
H	4.270179	9.668493	0.883521
C	5.362180	5.577178	2.688513
H	5.689355	5.694950	3.726407
C	0.166037	7.350424	2.805898
C	9.557636	7.141991	5.999743
C	1.071157	7.072343	0.571383
H	1.162932	6.475736	-0.332752
C	7.335892	8.469838	0.153511
H	7.793145	9.218572	-0.489177
C	9.151726	10.719587	5.578671
H	8.537160	10.133327	4.886389
C	3.418047	11.427350	7.859573
H	3.128372	11.360349	6.808127
C	8.180129	8.508679	8.592510
C	2.465256	10.342148	1.917970
H	2.264985	10.822524	2.880711
C	-0.948593	8.764690	8.452873
H	-1.211794	9.283203	7.524800
C	1.238790	10.085328	8.182185
C	7.838863	8.277185	1.439858
C	6.107772	6.582136	1.820524
C	1.632133	9.069025	1.828249
C	8.788529	12.184878	5.329921
H	9.434067	12.870321	5.890754
H	7.744614	12.378580	5.591771
H	8.911485	12.420202	4.269206
C	-2.135152	8.927428	9.408025
H	-2.013246	8.325961	10.314866
H	-3.055595	8.591144	8.919988
H	-2.272605	9.970389	9.715171
C	8.824119	10.258801	6.994854
C	5.648933	6.808235	0.519413
H	4.787290	6.251310	0.160493
C	6.259956	7.730066	-0.315841
H	5.888457	7.884707	-1.325708
C	8.477103	10.620963	2.038658
H	8.133286	10.996198	1.067986
H	9.290550	11.274482	2.374998
H	7.645931	10.706372	2.748533
C	-0.644027	6.802020	3.978333
H	-0.131803	7.102523	4.900406
C	8.826920	11.176584	8.044694
H	9.059082	12.217444	7.843909
C	8.195571	9.470228	9.606831
H	7.933719	9.173729	10.619656

C	8.974977	9.174291	1.914972
H	9.291494	8.843738	2.910711
C	1.741948	8.279409	0.680330
H	2.379554	8.616429	-0.133852
C	8.525958	10.788743	9.344068
H	8.536123	11.521242	10.147013
C	-0.009945	11.155253	6.423562
C	0.801851	8.608383	2.867006
C	5.667383	4.136180	2.259210
H	5.101402	3.428442	2.872771
H	6.731531	3.898247	2.365289
H	5.389224	3.973555	1.211091
C	-0.726614	7.292570	8.094416
H	0.108001	7.180822	7.396263
H	-1.621483	6.856411	7.635557
H	-0.486516	6.706337	8.989207
C	0.688498	9.156208	10.331517
H	0.021533	8.588846	10.974737
C	10.201884	9.107382	0.997211
H	11.017057	9.709147	1.413391
H	9.978450	9.501558	-0.000192
H	10.569495	8.083316	0.872999
C	-1.084402	10.662512	2.778940
H	-1.920941	11.331847	2.982515
H	-1.460334	9.730574	2.350829
H	-0.443385	11.125567	2.023423
C	4.856208	10.900911	7.961639
H	5.299440	11.102525	8.944029
H	5.487068	11.380780	7.205965
H	4.899156	9.818792	7.796237
C	9.408099	5.555805	2.522558
H	9.905941	6.226253	1.814627
H	8.589089	5.072998	1.984892
H	10.128029	4.802518	2.845522
C	-0.290699	10.406303	4.034132
C	-0.542136	11.247375	5.129826
H	-1.274274	12.029252	4.970602
C	1.878982	9.649276	10.850267
H	2.134590	9.468679	11.890697
C	2.740020	10.366223	10.036115
H	3.668540	10.754763	10.447073
C	2.444519	10.600703	8.689700
C	2.139377	11.343625	0.802428
H	2.747925	12.245974	0.921000
H	1.087555	11.647516	0.798135
H	2.362935	10.922513	-0.184095
C	-0.701599	5.272526	3.996236
H	-1.140113	4.933187	4.938074
H	0.298213	4.832070	3.915453
H	-1.320212	4.877651	3.182053
C	3.235831	6.851629	6.140586
O	6.446918	4.537838	7.208083
O	0.061563	4.507547	7.077335
C	5.315149	4.752880	7.168659
C	1.199287	4.712660	7.087865
C	3.102411	5.974144	8.889778
C	3.265219	3.159577	8.275549
C	3.262580	3.691840	5.463228
C	-2.066819	7.375646	4.025822
H	-2.075884	8.456356	4.196356
H	-2.630940	6.910492	4.841573
H	-2.599251	7.170397	3.089378
C	3.361580	12.906158	8.265116
H	3.634146	13.035510	9.318966
H	2.361079	13.330211	8.124781
H	4.061458	13.492305	7.660387

Int-1(W).log

Lowest Frequency = 11.2868cm-1

W	2.159013	-1.904187	-0.480922

Al	-0.876505	0.757891	-0.271061	C	2.126170	3.901062	1.618838
O	0.038263	-0.129342	1.040304	H	2.425579	4.077067	2.649343
O	-0.011624	-4.279323	-0.544968	C	-3.703509	-3.001002	-0.970821
N	-2.733961	0.545065	-0.369680	H	-3.766810	-3.710920	-1.791582
O	4.158754	0.616993	-0.475362	C	-3.163154	2.890823	-0.725328
O	2.379571	-2.138002	2.725282	H	-3.938453	3.616004	-0.938722
N	-0.809850	2.629481	-0.280225	C	-3.902389	-2.525957	1.375875
C	0.549044	-0.526886	-0.114428	H	-4.124771	-2.863586	2.385397
O	1.813951	-1.558809	-3.649277	C	-3.992428	-3.421626	0.319830
O	4.588852	-3.964629	-0.894139	H	-4.282329	-4.452428	0.503437
C	3.458663	-0.298155	-0.468166	C	-1.870751	3.401162	-0.525907
C	0.779951	-3.443010	-0.513883	C	-2.984235	-1.269046	-2.657721
C	3.713974	-3.222665	-0.757477	H	-2.882041	-0.177564	-2.678845
C	2.302727	-2.057295	1.580828	C	-0.427969	4.469956	3.270663
C	1.936110	-1.682365	-2.506899	H	0.474951	4.889027	3.728193
C	0.483496	3.209052	0.010761	H	-1.148677	4.278915	4.072619
C	-3.575312	1.554885	-0.608921	H	-0.850856	5.236090	2.611448
C	-3.228064	-0.796303	-0.146714	C	-5.042656	1.252155	-0.751861
C	-3.515143	-1.201266	1.169470	H	-5.609553	2.155825	-0.976791
C	0.839845	3.428805	1.353476	H	-5.429861	0.807309	0.169932
C	2.660592	3.906409	-0.723573	H	-5.202907	0.518112	-1.547946
H	3.380410	4.079518	-1.519362	C	-4.759658	-0.111583	3.078990
C	-3.410416	-0.261677	2.364421	H	-5.546946	0.234940	2.400401
H	-3.121839	0.730431	1.997716	H	-4.677954	0.609735	3.898722
C	1.383274	3.446372	-1.043676	H	-5.088918	-1.064230	3.507978
C	3.032470	4.133293	0.594324	C	0.439596	2.107745	3.471709
H	4.034389	4.485480	0.823133	H	0.640567	1.168409	2.948340
C	-2.325194	-0.725108	3.345132	H	-0.282901	1.908632	4.271399
H	-2.573626	-1.704086	3.770368	H	1.369383	2.445428	3.943091
H	-2.238050	-0.015339	4.175735	C	-1.680302	4.892682	-0.590617
H	-1.350387	-0.803068	2.854534	H	-0.963558	5.148407	-1.377842
C	-3.320542	-1.685145	-1.232259	H	-1.261871	5.264334	0.349448
C	-0.112517	3.172420	2.514994	H	-2.624430	5.400854	-0.788040
H	-1.056810	2.791692	2.108015	C	1.354316	4.370183	-3.406263
C	1.021078	3.180796	-2.498750	H	0.894775	5.297288	-3.045872
H	-0.062399	3.023202	-2.557279	H	0.992252	4.182253	-4.422161

H	2.434468	4.537959	-3.470884
C	1.702605	1.900225	-2.998251
H	1.429011	1.691993	-4.038155
H	1.414339	1.036002	-2.390911
H	2.793333	1.992448	-2.944042
C	-1.640932	-1.870059	-3.091652
H	-1.378022	-1.548990	-4.105207
H	-1.681766	-2.965205	-3.080807
H	-0.834175	-1.563065	-2.418084
C	-4.096395	-1.638282	-3.645800
H	-3.862439	-1.246481	-4.640890
H	-5.065388	-1.230205	-3.337533
H	-4.207610	-2.723632	-3.739754

Int-2(W).log

Lowest Frequency = 9.2300cm-1

W	2.154036	-1.741906	1.453122
Al	-1.176450	0.967874	-0.258264
O	-0.301106	0.177108	1.181885
O	0.306209	-4.369297	1.210204
N	-3.040641	0.773010	-0.311888
O	4.004481	0.883531	1.689437
O	0.818766	-1.047219	4.286079
N	-1.108273	2.843907	-0.287320
C	0.604366	-0.557014	0.583610
O	3.467998	-2.408250	-1.417763
O	4.470012	-3.493947	2.836138
C	3.348091	-0.058584	1.605382
C	0.970860	-3.434185	1.297978
C	3.642567	-2.868389	2.330854
C	1.304159	-1.300919	3.272107
C	2.951956	-2.142440	-0.426207
C	0.220864	3.394295	-0.449636
C	-3.899072	1.795581	-0.237815
C	-3.522000	-0.576813	-0.515033
C	-3.693157	-1.426769	0.592616
C	0.989799	3.710030	0.686119
C	2.068433	3.921914	-1.894112
H	2.495689	4.007716	-2.890385
C	-3.439592	-0.980976	2.027176
H	-3.033802	0.036064	2.004061
C	0.746715	3.498386	-1.752500
C	2.848666	4.223707	-0.786789
H	3.880257	4.538681	-0.916730
C	-2.401310	-1.873881	2.718337
H	-2.779774	-2.893018	2.855353
H	-2.153062	-1.472728	3.706211
H	-1.475609	-1.928052	2.139160
C	-3.729718	-1.026553	-1.833361
C	0.445388	3.637534	2.107765
H	-0.608578	3.340289	2.057345
C	-0.069148	3.186601	-3.002432
H	-1.063794	2.845725	-2.690481
C	2.309416	4.120155	0.487380
H	2.927357	4.355874	1.350272
C	-4.139007	-2.346657	-2.018999
H	-4.298079	-2.718527	-3.028270
C	-3.494280	3.135391	-0.155725
H	-4.284737	3.872667	-0.090433
C	-4.111833	-2.737090	0.351178
H	-4.254248	-3.411655	1.191853
C	-4.335604	-3.196020	-0.938473
H	-4.653220	-4.221875	-1.103403
C	-2.183856	3.632068	-0.220707
C	-3.487862	-0.140963	-3.049934
H	-3.305395	0.882311	-2.701354
C	0.513942	5.008008	2.796694
H	1.552765	5.318697	2.952217
H	0.032807	4.962953	3.779040
H	0.022308	5.793212	2.211955

C	-5.376255	1.511004	-0.291433
H	-5.955026	2.410002	-0.077640
H	-5.648870	0.724637	0.416896
H	-5.647709	1.147025	-1.288214
C	-4.741355	-0.945190	2.838572
H	-5.483551	-0.271128	2.397227
H	-4.543386	-0.604493	3.860053
H	-5.192872	-1.941638	2.900400
C	1.182444	2.586312	2.947367
H	1.058900	1.585778	2.525672
H	0.785399	2.572228	3.968091
H	2.254031	2.807122	3.005745
C	-1.993657	5.124514	-0.226538
H	-1.393930	5.431344	-1.088191
H	-1.445713	5.435920	0.668076
H	-2.952526	5.642719	-0.252212
C	-0.268052	4.447035	-3.855150
H	-0.747713	5.252843	-3.288983
H	-0.895087	4.224782	-4.724897
H	0.690914	4.826933	-4.224144
C	0.563554	2.066273	-3.837958
H	-0.064407	1.847424	-4.708982
H	0.684786	1.140914	-3.266266
H	1.550428	2.361878	-4.210902
C	-2.240925	-0.598597	-3.820245
H	-2.049500	0.070276	-4.667122
H	-2.382725	-1.609343	-4.219251
H	-1.343650	-0.617958	-3.192020
C	-4.707782	-0.092341	-3.977778
H	-4.533062	0.613149	-4.796567
H	-5.613154	0.220888	-3.446378
H	-4.909308	-1.071124	-4.425848
O	0.803051	-0.843431	-1.871492
C	0.264231	-0.313198	-0.931466

Int-3(W).log

Lowest Frequency = 15.5805cm-1

W	17.992052	5.713850	14.055527
Al	16.078177	2.410751	11.209320
Al	20.534941	1.519823	12.180906
O	17.551234	1.487528	10.948670
O	16.492868	3.502306	12.526922
O	19.983598	2.899241	13.169328
O	14.905783	6.366907	13.522843
N	21.099418	-0.022815	13.105887
N	14.486331	1.498094	11.569869
N	22.082284	1.774351	11.125960
O	20.992767	5.081229	15.015793
N	15.479403	3.458057	9.759912
C	17.682215	3.786880	13.035833
O	17.111937	4.733240	16.882132
C	18.619640	1.831512	11.690116
C	18.693394	2.829266	12.640290
O	18.460690	8.699456	15.075171
C	19.934731	5.259873	14.589108
C	16.028470	6.131216	13.678967
C	20.095334	-0.660151	13.929296
C	22.330890	-0.526547	13.030087
C	18.296104	7.611411	14.703660
C	17.450250	5.122179	15.834491
C	16.426778	3.918217	8.770606
C	13.321970	2.122457	11.341618
C	14.505357	0.231966	12.268027
C	14.614348	0.199749	13.667984
C	23.186284	1.029302	11.247725
C	17.254779	5.027025	9.039841
C	17.501988	3.590305	6.642485
H	17.606096	3.037923	5.711165
C	14.755180	1.454395	14.519471
H	14.661339	2.331789	13.871524

C	19.324370	-1.698291	13.370898
C	16.538772	3.192157	7.569969
C	23.318969	-0.013908	12.174825
H	24.277745	-0.517267	12.194806
C	18.331660	4.674235	6.892346
H	19.080718	4.968819	6.161653
C	19.857856	-0.184029	15.231864
C	16.147095	1.509714	15.163210
H	16.274775	0.696296	15.887447
H	16.293157	2.460095	15.688272
H	16.943199	1.407028	14.417843
C	14.459826	-0.950659	11.498300
C	17.130237	5.870866	10.304592
H	16.468237	5.351076	11.001535
C	15.668871	1.978225	7.266585
H	14.881627	1.917410	8.026210
C	18.201975	5.383142	8.077640
H	18.855782	6.231420	8.265472
C	14.470069	-2.170112	12.174518
H	14.427689	-3.097420	11.611052
C	22.008759	2.784961	10.094138
C	13.199464	3.239446	10.502810
H	12.205818	3.659094	10.406770
C	14.632439	-1.049941	14.294209
H	14.719010	-1.096101	15.377241
C	19.537486	-2.211140	11.951810
H	20.416408	-1.710118	11.530389
C	14.548962	-2.222624	13.562144
H	14.556065	-3.184440	14.069183
C	22.224600	4.138075	10.432732
C	14.192029	3.798111	9.678030
C	22.684078	-1.730992	13.862237
H	22.398987	-1.578129	14.906239
H	23.751737	-1.945407	13.805270
H	22.129902	-2.606093	13.507513
C	14.409031	-0.908346	9.972563
H	15.016932	-0.051522	9.655019
C	20.641426	0.961978	15.859832
H	21.305048	1.384423	15.098971
C	21.348576	0.948761	8.423019
H	21.662268	0.302840	9.250400
C	16.504269	7.239489	10.003979
H	17.124178	7.811107	9.303602
H	16.408242	7.820787	10.927532
H	15.504856	7.144779	9.568047
C	18.314232	-2.262180	14.149769
H	17.691451	-3.048948	13.729755
C	18.851796	-0.802211	15.978370
H	18.658768	-0.456029	16.991030
C	12.072055	1.619999	12.016387
H	11.988434	0.532948	11.956181
H	11.182688	2.077569	11.581704
H	12.115183	1.882577	13.079100
C	19.835131	0.773219	8.247670
H	19.283652	1.035658	9.157368
H	19.591999	-0.264122	7.987113
H	19.465576	1.421908	7.444292
C	22.125266	5.081864	9.407850
H	22.291668	6.130679	9.631797
C	13.667708	1.549814	15.596673
H	12.658825	1.497155	15.172610
H	13.761272	2.496332	16.137999
H	13.758973	0.740389	16.329659
C	18.483135	6.065673	11.007389
H	19.007209	5.123380	11.186244
H	18.329236	6.625975	11.957988
H	19.153993	6.712472	10.429143
C	21.573798	3.379533	7.804583
H	21.300994	3.098773	6.790104
C	21.668261	2.393111	8.787226
C	22.560895	4.573905	11.857902
H	21.919740	3.999202	12.535616

C	18.335743	-1.872516	11.060569
H	17.425070	-2.341274	11.453537
H	18.499247	-2.247595	10.042399
H	18.152846	-0.794163	11.006023
C	18.083076	-1.827028	15.447338
H	17.294481	-2.282237	16.041659
C	24.347824	1.265767	10.314692
H	24.132379	0.804807	9.344070
H	25.257334	0.813225	10.712505
H	24.517816	2.329231	10.137550
C	19.812146	-3.720432	11.927923
H	18.940163	-4.288027	12.270728
H	20.656153	-3.995198	12.569799
H	20.040159	-4.049744	10.908632
C	13.745328	4.769716	8.618338
H	14.460521	5.584592	8.492131
H	12.763222	5.178203	8.859815
H	13.673428	4.249855	7.656065
C	14.978448	2.086192	5.901855
H	14.409998	3.017520	5.805850
H	14.286579	1.249070	5.760782
H	15.702059	2.055007	5.080076
C	22.256575	6.050720	12.124336
H	22.933501	6.716426	11.575557
H	22.383579	6.258003	13.189876
H	21.225586	6.308549	11.857922
C	16.488941	0.685673	7.356712
H	15.849910	-0.185767	7.174080
H	16.957263	0.574790	8.341882
H	17.287228	0.677526	6.605737
C	21.509753	0.465550	17.023527
H	20.891634	0.044031	17.824492
H	22.083810	1.295644	17.447777
H	22.219455	-0.308315	16.709672
C	22.096512	0.480831	7.169114
H	21.750954	1.008550	6.273640
H	21.924586	-0.587786	7.003724
H	23.176642	0.643990	7.251874
C	21.809024	4.711288	8.107176
H	21.737439	5.468460	7.329754
C	19.712645	2.091609	16.321163
H	19.117419	2.471437	15.486773
H	20.303914	2.927063	16.709748
H	19.036271	1.760862	17.117896
C	24.026132	4.290137	12.218439
H	24.256076	3.221297	12.229198
H	24.243696	4.678711	13.218531
H	24.704597	4.782053	11.510455
C	15.005518	-2.156408	9.315945
H	15.067958	-2.010455	8.232845
H	14.378927	-3.040114	9.481460
H	16.011258	-2.371814	9.687016
C	12.986627	-0.689143	9.436211
H	12.575421	0.282833	9.719925
H	12.310671	-1.469459	9.805208
H	12.986582	-0.735708	8.341076

TS-1(W).log

Lowest Frequency = -153.2519cm-1

W	2.717431	-1.118495	-0.414665
Al	-1.008358	0.317441	0.003306
O	0.510606	-0.415803	1.827171
O	2.002664	-4.242984	-0.150298
N	-2.693709	-0.396861	0.595683
O	3.573926	1.964655	-0.770713
O	4.189706	-1.226339	2.444513
N	-1.366597	2.109287	0.624884
C	0.939297	-0.540056	0.709518
O	0.930042	-0.891135	-3.067966
O	5.392307	-1.955192	-1.951611

C	3.254617	0.867994	-0.632463	C	-2.485782	2.513190	1.227760
C	2.245362	-3.121403	-0.238195	C	-3.919514	-0.808830	-2.028052
C	4.421879	-1.650681	-1.404815	H	-3.786091	0.133318	-1.483793
C	3.671517	-1.189415	1.418484	C	0.476473	3.830851	3.633231
C	1.603884	-0.991927	-2.138988	H	1.173593	4.674846	3.579319
C	-0.368924	3.087970	0.248172	H	0.580309	3.374247	4.623152
C	-3.646664	0.289220	1.236354	H	-0.539269	4.231626	3.558244
C	-2.972193	-1.751933	0.170576	C	-4.949799	-0.392839	1.567811
C	-2.607965	-2.833283	0.993241	H	-5.568786	0.239878	2.204863
C	0.668995	3.407768	1.145163	H	-4.780665	-1.351476	2.063013
C	0.567539	4.562966	-1.406958	H	-5.500656	-0.607933	0.645804
H	0.543303	5.006429	-2.399719	C	-2.839817	-3.106645	3.498441
C	-1.933713	-2.651572	2.346816	H	-3.780966	-2.547883	3.530745
H	-1.721529	-1.586700	2.483993	H	-2.333624	-2.962523	4.458637
C	-0.428006	3.657416	-1.038284	H	-3.087361	-4.170353	3.407682
C	1.587761	4.904175	-0.531135	C	2.165616	2.184115	2.779564
H	2.355028	5.610526	-0.835521	H	2.441312	1.487633	1.983849
C	-0.594870	-3.397919	2.403873	H	2.173216	1.633092	3.725327
H	-0.741757	-4.483690	2.385806	H	2.941690	2.955527	2.837593
H	-0.060047	-3.146554	3.325346	C	-2.699431	3.981019	1.497986
H	0.047346	-3.134117	1.559582	H	-2.932989	4.487485	0.554306
C	-3.560640	-1.957519	-1.092764	H	-1.803512	4.457594	1.897962
C	0.780749	2.798602	2.538405	H	-3.532753	4.132857	2.185267
H	0.045901	1.991722	2.618698	C	-2.270285	4.560864	-2.523247
C	-1.519922	3.311993	-2.044002	H	-2.683597	5.132402	-1.685263
H	-2.253791	2.665054	-1.548420	H	-3.096872	4.278709	-3.183684
C	1.631261	4.330530	0.731127	H	-1.611879	5.230623	-3.086759
H	2.439531	4.593399	1.408973	C	-0.945227	2.536686	-3.237339
C	-3.793234	-3.268580	-1.508171	H	-1.742094	2.260137	-3.936955
H	-4.239499	-3.447583	-2.483594	H	-0.438876	1.618253	-2.919373
C	-3.530922	1.643095	1.569969	H	-0.215646	3.145318	-3.783104
H	-4.378115	2.087050	2.078209	C	-2.980601	-0.782640	-3.242121
C	-2.872797	-4.125953	0.535678	H	-3.209814	0.072629	-3.888068
H	-2.602427	-4.974499	1.159649	H	-3.092138	-1.694407	-3.839613
C	-3.460456	-4.347560	-0.700881	H	-1.928674	-0.712682	-2.942482
H	-3.651286	-5.361988	-1.040254	C	-5.383917	-0.865812	-2.479210

H	-5.628702	0.012418	-3.085630
H	-6.071103	-0.891813	-1.626647
H	-5.581431	-1.752589	-3.090870

TS-2(W).log

Lowest Frequency = -276.3364cm-1

W	-2.615627	-1.473720	-0.049945
Al	1.127553	0.448699	0.295403
O	0.333417	-0.694848	-0.843524
O	-1.456467	-3.634269	2.032831
N	2.990240	0.283949	0.137664
O	-3.857392	0.672262	-2.100761
O	-1.407416	-3.095819	-2.550283
N	0.992936	2.311751	0.114286
C	-0.676725	-0.553725	-0.007261
O	-3.686611	0.312428	2.395826
O	-5.384491	-3.096357	-0.172412
C	-3.391831	-0.080370	-1.362441
C	-1.879712	-2.868184	1.284216
C	-4.397184	-2.499931	-0.117964
C	-1.855871	-2.530845	-1.653622
C	-3.273349	-0.325223	1.526888
C	-0.349819	2.837475	-0.021962
C	3.819446	1.332845	0.103127
C	3.520210	-1.049373	-0.060070
C	3.619615	-1.546728	-1.375303
C	-0.918774	2.935541	-1.303657
C	-2.394295	3.582144	0.991504
H	-2.979169	3.831767	1.872219
C	3.206292	-0.743843	-2.604281
H	2.778295	0.207378	-2.267610
C	-1.072465	3.162948	1.142468
C	-2.981197	3.672305	-0.264362
H	-4.016537	3.987724	-0.358652
C	2.126069	-1.470807	-3.416121
H	2.510654	-2.403879	-3.842953
H	1.797087	-0.839515	-4.249385
H	1.255187	-1.703162	-2.797908
C	3.874988	-1.835256	1.051246
C	-0.147469	2.619318	-2.580550
H	0.884222	2.366322	-2.307607
C	-0.444271	3.114064	2.532120
H	0.416136	2.435095	2.491898
C	-2.246306	3.357029	-1.397432
H	-2.716181	3.423781	-2.375419
C	4.369810	-3.120089	0.818147
H	4.651893	-3.742284	1.664303
C	3.378888	2.661939	0.094527
H	4.147711	3.423655	0.049008
C	4.121883	-2.835891	-1.552075
H	4.210122	-3.237289	-2.558768
C	4.500659	-3.617621	-0.469471
H	4.887430	-4.620219	-0.630013
C	2.054094	3.117377	0.017931
C	3.724940	-1.354335	2.488721
H	3.306846	-0.341533	2.468217
C	-0.091409	3.842797	-3.505771
H	-1.087692	4.096982	-3.883310
H	0.546650	3.637478	-4.371658
H	0.300427	4.726989	-2.991491
C	5.304458	1.090893	0.040961
H	5.564373	0.508701	-0.847213
H	5.627381	0.503496	0.905112
H	5.853382	2.032678	0.024233
C	4.414462	-0.418175	-3.493105
H	5.181424	0.151952	-2.958017
H	4.100374	0.173856	-4.359100
H	4.883369	-1.334232	-3.869157
C	-0.734029	1.412996	-3.325689
H	-0.707333	0.508013	-2.711463

H	-0.158909	1.219790	-4.238496
H	-1.774138	1.596556	-3.615782
C	1.862838	4.585420	-0.264087
H	1.910437	4.738292	-1.348601
H	2.667468	5.168174	0.188919
H	0.898359	4.958444	0.079711
C	0.089838	4.494013	2.944482
H	0.877883	4.852436	2.275652
H	0.504571	4.454290	3.957285
H	-0.718757	5.233809	2.939664
C	-1.399087	2.588537	3.608510
H	-0.848856	2.421111	4.540141
H	-1.874902	1.649753	3.316473
H	-2.191724	3.312082	3.828464
C	2.750430	-2.248228	3.267986
H	2.556359	-1.830024	4.261154
H	3.163363	-3.253786	3.404274
H	1.791702	-2.355908	2.752060
C	5.073870	-1.290087	3.216764
H	4.938983	-0.910858	4.235053
H	5.788739	-0.636241	2.706538
H	5.529938	-2.283438	3.290045
O	-0.397377	-0.496778	2.866884
C	0.342683	-0.153740	2.044912

TS-3(W).log

Lowest Frequency = -289.9492cm-1

W	3.478521	19.049025	3.727063
Al	-0.350697	18.555225	6.093020
Al	2.617373	14.999515	5.619397
O	3.541040	16.318403	4.801650
O	0.107481	16.947351	5.717515
N	-1.218097	18.825435	7.746091
O	1.248274	19.399336	5.957927
N	2.011852	13.267474	5.036206
N	3.868973	14.337744	6.909690
N	-1.626262	19.398086	4.998189
C	1.382658	16.519893	5.453942
C	-2.525209	19.120700	7.822083
C	4.948563	16.113741	8.256175
C	1.194044	13.198955	3.845916
C	-0.502169	18.666134	8.992627
C	-2.888495	19.593239	5.390252
C	-0.200086	13.028552	3.948408
C	-0.949502	12.995939	5.275871
H	-0.214447	12.941142	6.087136
C	5.018781	15.157122	7.234737
C	-1.272583	19.853902	3.668450
C	2.261177	12.139037	5.706588
C	-3.322696	19.398506	6.709387
H	-4.372290	19.579885	6.901975
C	-1.751576	14.290528	5.468478
H	-1.104216	15.173007	5.463807
H	-2.293835	14.262143	6.423056
H	-2.496033	14.400939	4.669358
C	3.847180	13.094946	7.396726
C	3.661360	16.407382	9.004315
H	2.906318	15.682953	8.679048
C	-1.110548	16.164117	9.024071
H	-1.655989	16.455472	8.120013
C	-0.845684	21.185300	3.514587
C	-0.012407	15.183518	8.592040
H	0.634547	15.641212	7.837574
H	-0.456961	14.281791	8.154323
H	0.600663	14.878161	9.449074
C	-0.490894	17.414698	9.636647
C	3.032879	12.081475	6.877082
H	3.108799	11.111288	7.352828
C	1.826035	13.320892	2.590915
C	-0.938993	12.919968	2.767672

H	-2.016175	12.781580	2.826832
C	0.199611	21.135567	8.841307
H	-0.334731	21.046406	7.889494
C	-1.719395	17.504828	2.729951
H	-1.937095	17.305781	3.785194
C	6.200947	14.972069	6.492247
C	1.040209	13.210688	1.443149
H	1.505883	13.295942	0.466510
C	-0.329205	12.997140	1.525377
H	-0.922166	12.909235	0.618845
C	0.141222	19.787467	9.548083
C	-0.608171	22.117611	4.696809
H	-0.743634	21.541518	5.618748
C	-1.415846	18.988224	2.570714
C	-0.480855	16.678093	2.358837
H	-0.219951	16.821264	1.303027
H	-0.670743	15.611961	2.520913
H	0.385104	16.956596	2.967054
C	6.092689	16.856944	8.553966
H	6.049543	17.606467	9.340910
C	-3.192130	19.184296	9.172631
H	-3.123411	18.220999	9.685286
H	-4.242241	19.458334	9.070154
H	-2.693107	19.916940	9.813665
C	1.709383	10.824969	5.207833
H	0.648211	10.737835	5.456138
H	2.238416	9.991711	5.672144
H	1.795917	10.746802	4.121636
C	3.819724	16.255950	10.521117
H	4.177166	15.256343	10.793357
H	2.859252	16.424339	11.019787
H	4.532458	16.984228	10.924038
C	2.415996	17.186051	4.872979
C	-0.645979	21.661375	2.217826
H	-0.322872	22.689366	2.071301
C	0.138304	17.324734	10.879612
H	0.154011	16.369285	11.399422
C	7.274354	16.666211	7.855865
H	8.154361	17.255877	8.097628
C	-1.622665	23.267933	4.723565
H	-1.546497	23.881037	3.818642
H	-1.438202	23.919570	5.584551
H	-2.653216	22.902983	4.795222
C	-1.884594	11.784192	5.396888
H	-2.732172	11.868090	4.708011
H	-2.295761	11.726063	6.410285
H	-1.381730	10.836365	5.178930
C	-1.214995	19.517227	1.295959
H	-1.319996	18.869641	0.429071
C	-2.100866	15.474707	9.970659
H	-2.582777	14.634036	9.460312
H	-2.887933	16.153723	10.315767
H	-1.597575	15.074315	10.857521
C	-0.853542	20.844783	1.115551
H	-0.696098	21.233429	0.113375
C	-3.913751	20.071463	4.394365
H	-3.616848	21.028002	3.956100
H	-4.889593	20.181788	4.868025
H	-3.994143	19.361329	3.566154
C	7.318594	15.735040	6.827296
H	8.237865	15.608507	6.259950
C	3.155821	17.806935	8.634826
H	3.867610	18.577373	8.955158
H	2.198490	18.011393	9.120497
H	3.025378	17.899157	7.551447
C	0.827040	22.657731	4.703512
H	1.559248	21.844748	4.694625
H	0.995937	23.265812	5.599938
H	1.020964	23.293601	3.832714
C	6.290320	14.024057	5.302823
H	5.346831	13.471919	5.224649
C	4.787749	12.717096	8.513928

H	5.820127	12.689687	8.151907
H	4.532246	11.736336	8.917731
H	4.754933	13.461579	9.314224
C	3.332912	13.531683	2.475458
H	3.638790	14.224488	3.269183
C	-2.933869	17.053823	1.910925
H	-3.839717	17.615890	2.165276
H	-3.132545	15.992131	2.094676
H	-2.760020	17.172962	0.835853
C	0.744586	18.429406	11.463558
H	1.223652	18.337661	12.434834
C	1.647928	21.524953	8.519245
H	2.235094	21.661252	9.434649
H	1.671842	22.468572	7.962969
H	2.135083	20.760477	7.906937
C	0.755324	19.644524	10.793564
H	1.253109	20.500913	11.242376
C	2.149802	19.181384	5.111698
C	-0.496958	22.233633	9.653971
H	-1.544309	21.985722	9.858815
H	-0.476603	23.181422	9.105812
H	0.002299	22.395453	10.615796
C	3.753370	14.192162	1.160972
H	3.640301	13.513328	0.307390
H	4.806599	14.481783	1.216394
H	3.172699	15.098835	0.964400
C	4.091860	12.210881	2.666182
H	3.936231	11.781725	3.661073
H	5.168873	12.366370	2.538166
H	3.768347	11.473746	1.921490
C	6.460080	14.832484	4.010454
H	5.637304	15.543886	3.895351
H	6.482045	14.170117	3.136562
H	7.400238	15.395950	4.021489
C	7.409580	12.988470	5.459145
H	8.395063	13.465453	5.498246
H	7.409840	12.300797	4.606233
H	7.291467	12.392699	6.371173
C	5.089022	18.976036	5.038382
O	6.007621	18.986997	5.727973
C	4.123640	20.867799	3.225851
O	4.427487	21.964507	2.985478
C	4.599332	17.810721	2.485765
O	5.187225	17.072309	1.826234
C	2.329353	19.237039	2.029257
O	1.820237	19.360269	1.002044

TS-4(W).log

Lowest Frequency = -319.2650cm-1

W	-1.816083	-2.147054	-2.465205
Al	-2.017691	0.979688	1.273242
Al	2.564225	-0.152259	-0.310866
O	-2.184588	-0.096385	-0.125438
N	3.464610	1.031787	-1.478152
O	-0.295384	1.343318	1.219877
O	1.196087	-1.061645	-0.946803
O	-2.684100	-4.288252	-4.666368
N	3.963744	-1.417442	-0.075787
N	-2.690702	0.175686	2.847715
N	-2.945655	2.611557	1.342675
C	-3.408300	-2.173335	2.722511
C	-3.993790	0.130686	4.942784
H	-3.302080	-0.633953	5.297674
H	-4.950105	-0.358916	4.729070
H	-4.164285	0.864610	5.733040
C	5.693104	-2.854587	-1.083303
H	6.616435	-2.658669	-1.631410
H	5.164970	-3.670041	-1.590379
H	5.931665	-3.190613	-0.072906
C	3.191148	2.450439	-1.518890

C	4.909045	-0.773462	-2.190219
H	5.590526	-1.086524	-2.972506
O	-1.416710	-0.051742	-4.859102
C	2.961288	-5.341471	-0.433481
H	2.785532	-6.110073	0.328204
H	4.036587	-5.325874	-0.640695
H	2.441317	-5.650867	-1.345810
C	0.955779	-4.067050	0.390143
H	0.393724	-4.497969	-0.443884
H	0.532286	-3.081025	0.605737
H	0.795598	-4.710668	1.263661
O	-2.436900	-4.372860	-0.227175
C	3.988831	-2.226366	1.120778
C	0.101183	-0.414669	-0.387767
O	2.180013	1.544925	2.350963
C	0.488296	0.570718	0.486962
C	-3.201239	4.505108	-1.855592
H	-3.754040	4.477064	-2.789110
C	2.430131	0.867160	1.444575
C	4.367417	0.511537	-2.323094
C	2.440730	-3.971988	0.023667
H	2.517128	-3.277366	-0.816855
C	-0.413545	2.350200	-2.457092
H	-1.095414	1.562989	-2.790888
H	-0.745592	3.287035	-2.917570
H	-0.519273	2.458424	-1.372779
C	6.965800	-0.717095	1.770568
H	7.527004	0.223097	1.718391
H	7.028892	-1.199459	0.789565
H	7.462344	-1.369027	2.498556
C	0.649356	4.400101	1.382641
H	0.767891	3.469077	0.824679
H	1.261623	4.330708	2.288335
H	1.039677	5.221802	0.771839
C	1.028655	2.005255	-2.847151
H	1.219902	0.971619	-2.540169

C	-3.417872	3.513259	-0.898238
C	4.805043	-1.637028	-1.082013
C	-2.271544	5.516833	-1.649905
H	-2.120580	6.277984	-2.411367
C	3.823500	4.707330	-0.957933
H	4.514913	5.406310	-0.492668
C	5.511455	-0.451688	2.181029
H	5.064481	0.182138	1.407289
C	-0.080941	-0.624292	3.946457
H	-0.113407	0.240989	3.275865
C	3.255806	-3.425684	1.185584
C	-0.820498	4.640703	1.753975
H	-1.122732	3.839551	2.436657
C	-4.800843	-1.779965	2.242535
H	-4.942969	-0.707595	2.410170
C	-2.411375	-1.233347	3.045679
C	4.098196	3.339942	-0.906865
C	2.025770	2.922207	-2.152723
C	-1.709066	4.581517	0.515730
C	5.457357	0.330643	3.496642
H	5.959570	-0.199872	4.312545
H	4.423629	0.527815	3.799459
H	5.963230	1.293677	3.381564
C	-5.907519	-2.511397	3.012002
H	-5.906642	-3.584597	2.794621
H	-6.888096	-2.121856	2.719465
H	-5.800505	-2.394384	4.096304
C	4.712038	-1.748232	2.235063
C	1.799323	4.300431	-2.177192
H	0.902742	4.680236	-2.660502
C	2.688563	5.189703	-1.592757
H	2.495045	6.259034	-1.628066
C	5.294576	3.212691	1.307287
H	5.206281	4.293274	1.465252
H	6.208253	2.877606	1.811468
H	4.437824	2.735532	1.792271

C	-4.405563	2.380834	-1.172093
H	-3.986224	1.462452	-0.742853
C	4.711918	-2.514576	3.400469
H	5.269311	-2.171388	4.267084
C	3.285508	-4.151200	2.380406
H	2.730282	-5.084233	2.443370
C	5.362778	2.882751	-0.189047
H	5.447246	1.794601	-0.289069
C	-1.520073	5.537222	-0.485843
H	-0.765698	6.308518	-0.346832
C	4.007719	-3.709434	3.476753
H	4.021615	-4.291772	4.394502
C	-3.492939	0.821452	3.698419
C	-2.691031	3.593878	0.307895
C	1.203479	2.075381	-4.371089
H	0.463060	1.442759	-4.868252
H	2.197761	1.738162	-4.682909
H	1.068842	3.103127	-4.728694
C	-4.934134	-2.011825	0.735468
H	-4.164933	-1.453312	0.196809
H	-5.915686	-1.685426	0.374633
H	-4.816314	-3.073583	0.490088
C	-3.089274	-3.528470	2.816715
H	-3.833830	-4.271217	2.543772
C	6.626307	3.507974	-0.796485
H	7.521619	3.086934	-0.326689
H	6.648094	4.591454	-0.635661
H	6.696759	3.336562	-1.874977
C	-4.375477	4.313946	2.398577
H	-5.170151	4.344119	3.145027
H	-4.784212	4.576107	1.420709
H	-3.634481	5.076831	2.652732
C	1.344688	-1.179174	3.895929
H	1.508125	-1.959279	4.648063
H	2.059914	-0.378173	4.105262
H	1.587082	-1.608324	2.916105
C	4.906801	1.351802	-3.453665
H	4.176832	2.079278	-3.810440
H	5.215108	0.708220	-4.279767
H	5.788438	1.905165	-3.114614
C	-3.723403	2.954682	2.377782
C	-3.959153	2.126991	3.482354
H	-4.602905	2.532923	4.253356
C	-1.830889	-3.942545	3.222531
H	-1.596691	-5.002590	3.270916
C	-0.869996	-3.001539	3.565630
H	0.111131	-3.341214	3.882883
C	-1.134170	-1.632147	3.492369
C	-0.948140	5.977445	2.497997
H	-0.346688	5.958133	3.412826
H	-1.979899	6.210928	2.779325
H	-0.582654	6.806498	1.882009
C	-4.595648	2.108142	-2.666883
H	-5.178019	1.193208	-2.798683
H	-3.635223	1.971749	-3.176199
H	-5.138357	2.919922	-3.164969
C	-1.242763	-0.729872	-0.823015
O	1.168608	-3.255342	-2.966992
O	-4.865352	-1.175845	-2.378382
C	0.137768	-2.808318	-2.711506
C	-3.756046	-1.503103	-2.365344
C	-2.196410	-3.558846	-1.008090
C	-2.365772	-3.506985	-3.871472
C	-1.527723	-0.782691	-3.970234
C	-5.769700	2.625845	-0.513012
H	-5.704661	2.661638	0.578580
H	-6.462123	1.819268	-0.775606
H	-6.204153	3.570305	-0.862442
C	-0.378703	-0.116781	5.364891
H	-0.422153	-0.951879	6.074186
H	-1.326795	0.425768	5.419363
H	0.410215	0.567545	5.694601

Al1.log

SCF (wB97x) = -1241.09457222

G(298 K)= -1240.516275

Lowest Frequency = 11.4330cm-1

Al	9.600739	1.420201	3.168886
N	8.768633	2.828116	4.360595
N	10.236588	2.918529	1.966450
C	8.660248	4.141195	4.163286
C	9.193430	4.797275	3.046624
H	9.032372	5.867043	2.988271
C	9.967619	4.221710	2.031184
C	7.947911	4.993690	5.187090
H	6.927678	4.632385	5.348097
H	7.910375	6.037246	4.871412
H	8.457509	4.934305	6.154161
C	10.527442	5.152656	0.981278
H	10.222158	4.834138	-0.020049
H	11.621877	5.131088	0.996834
H	10.192398	6.177606	1.146518
C	8.253466	2.274138	5.585724
C	6.935432	1.783734	5.633405
C	6.490652	1.185525	6.813317
H	5.475758	0.796963	6.863170
C	7.320404	1.070622	7.919556
H	6.957138	0.598289	8.828386
C	8.618472	1.557700	7.857044
H	9.269006	1.460049	8.723488
C	9.107876	2.163811	6.699472
C	5.994459	1.866490	4.439108
H	6.490221	2.455658	3.660161
C	5.720318	0.471499	3.863037
H	6.651500	-0.025965	3.572051
H	5.073973	0.538500	2.980837
H	5.217432	-0.164020	4.601072
C	4.680650	2.575660	4.788866
H	4.855517	3.574413	5.203434
H	4.102132	2.007995	5.526022
H	4.057534	2.684480	3.894838
C	10.547606	2.661223	6.668870
H	10.697618	3.205431	5.730093
C	10.846779	3.633551	7.815802
H	10.153082	4.481296	7.818533
H	11.864144	4.028539	7.724196
H	10.771928	3.140601	8.791336
C	11.531123	1.484080	6.683033
H	11.347247	0.804162	5.843832
H	11.434642	0.904325	7.608223
H	12.564818	1.842496	6.615585
C	11.112307	2.450090	0.923875
C	12.495893	2.374956	1.174355
C	13.324798	1.849561	0.182568
H	14.395080	1.779480	0.364330
C	12.807341	1.407689	-1.027059
H	13.467624	0.998009	-1.786809
C	11.441569	1.487123	-1.258772
H	11.038002	1.133949	-2.205289
C	10.572712	2.004754	-0.296961
C	13.105746	2.824095	2.496305
H	12.319888	3.311865	3.083196
C	13.604593	1.618736	3.303449
H	12.797259	0.899977	3.482131
H	14.000714	1.939758	4.273801
H	14.404864	1.093878	2.769101
C	14.230124	3.846749	2.295569
H	13.889296	4.714858	1.720997
H	15.081443	3.409504	1.762324
H	14.596906	4.203915	3.263718
C	9.079160	2.050757	-0.589799
H	8.590185	2.577757	0.236546

C	8.768475	2.822334	-1.878027
H	9.180632	3.836971	-1.853684
H	7.685739	2.900141	-2.023049
H	9.182960	2.317772	-2.757780
C	8.491408	0.635216	-0.651085
H	8.672872	0.091199	0.281830
H	8.942645	0.059854	-1.467665
H	7.410158	0.673527	-0.824144

CO.log

Lowest Frequency = 2252.2364cm-1

O	17.721534	7.813931	17.341390
C	17.720169	6.853625	16.735170

MnCpCO3.log

Lowest Frequency = 19.3200cm-1

Mn	3.056684	18.970681	4.204298
O	0.614145	19.978979	5.514768
C	1.575510	19.569406	5.031492
C	2.917187	18.234506	2.199050
H	2.486644	17.275865	1.939715
C	4.287783	18.481647	2.518313
C	4.435626	19.867816	2.794779
C	3.153763	20.470571	2.673837
H	2.935406	21.521124	2.818356
C	2.219955	19.458342	2.294548
H	1.160979	19.601706	2.122187
C	5.722821	20.578116	3.095429
H	5.556169	21.453115	3.729460
H	6.187319	20.919793	2.164729
H	6.431916	19.918935	3.603405
C	4.141608	19.503087	5.535596
O	4.873761	19.869406	6.345415
C	2.898391	17.302190	4.857895
O	2.810347	16.214907	5.226158
H	5.085715	17.749821	2.523406

WCO6.log

Lowest Frequency = 62.3678cm-1

W	0.000003	0.000003	0.000000
C	-2.075036	-0.000007	0.000000
C	-0.000001	1.467212	1.467207
C	-0.000001	1.467212	-1.467207
C	-0.000001	-1.467207	1.467204
C	-0.000001	-1.467207	-1.467204
C	2.075040	-0.000003	0.000000
O	-3.221695	-0.000015	0.000000
O	-0.000007	2.278036	2.278026
O	-0.000007	2.278036	-2.278026
O	-0.000009	-2.278036	2.278019
O	-0.000009	-2.278036	-2.278019
O	3.221699	-0.000007	0.000000

6 References

(1) Cui, C.; Roesky, H. W.; Schmidt, H.-G.; Noltemeyer, M.; Hao, H.; Cimpoesu, F. *Angew. Chem., Int. Ed.* **2000**, *39*, 4274–4276.

(2) Agbossou, F.; O'Connor, E. J.; Garner, C. M.; Méndez, N. Q.; Fernández, J. M.; Patton, A. T.; Ramsden, J. A.; Gladysz, J. A.; O'Connor, J. M.; Tajima, T.; Gable, K. P. In *Inorganic Syntheses*; John Wiley & Sons, Ltd, 1992; pp 211–225.

(3) Kong, R. Y.; Crimmin, M. R. *J. Am. Chem. Soc.* **2018**, *140*, 13614–13617.

(4) Pyykkö, P.; Atsumi, M. *Chem. Eur. J.* **2008**, *15*, 186–197.

(5) Frisch, M. J.; Trucks, G. W.; Schlegel, H. B.; Scuseria, G. E.; Robb, M. A.; Cheeseman, J. R.; Scalmani, G.; Barone, V.; Mennucci, B.; Petersson, G. A.; Nakatsuji, H.; Caricato, M.; Li, X.; Hratchian, H. P.; Izmaylov, A. F.; Bloino, J.; Zheng, G.; Sonnenberg, J. L.; Hada, M.; Ehara, M.; Toyota, K.; Fukuda, R.; Hasegawa, J.; Ishida, M.; Nakajima, T.; Honda, Y.; Kitao, O.; Nakai, H.; Vreven, T.; Montgomery, J. A., Jr.; Peralta, J. E.; Ogliaro, F.; Bearpark, M.; Heyd, J. J.; Brothers, E.; Kudin, K. N.; Staroverov, V. N.; Kobayashi, R.; Normand, J.; Raghavachari, K.; Rendell, A.; Burant, J. C.; Iyengar, S. S.; Tomasi, J.; Cossi, M.; Rega, N.; Millam, J. M.; Klene, M.; Knox, J. E.; Cross, J. B.; Bakken, V.; Adamo, C.; Jaramillo, J.; Gomperts, R.; Stratmann, R. E.; Yazyev, O.; Austin, A. J.; Cammi, R.; Pomelli, C.; Ochterski, J. W.; Martin, R. L.; Morokuma, K.; Zakrzewski, V. G.; Voth, G. A.; Salvador, P.; Dannenberg, J. J.; Dapprich, S.; Daniels, A. D.; Farkas, Ö.; Foresman, J. B.; Ortiz, J. V.; Cioslowski, J.; Fox, D. J. Gaussian, Inc., Wallingford, CT 2009.

(6) Hooper, T. N.; Garçon, M.; White, A. J. P.; Crimmin, M. R. *Chem. Sci.* **2018**, *9*, 5435–5440.

(7) Kong, R. Y.; Crimmin, M. R. *Chem. Commun.* **2019**, *55*, 6181–6184.

(8) Chai, J.-D.; Head-Gordon, M. *J. Chem. Phys.* **2008**, *128*, 084106.

(9) Stephens, P. J.; Devlin, F. J.; Chabalowski, C. F.; Frisch, M. J. *J. Phys. Chem.* **1994**, *98*, 11623–11627.

(10) Zhao, Y.; Truhlar, D. G. *Theor. Chem. Acc.* **2008**, *120*, 215–241.

(11) Becke, A. D. *J. Chem. Phys.* **1993**, *98*, 5648–5652.

(12) Zhao, Y.; Truhlar, D. G. *J. Chem. Phys.* **2006**, *125*, 194101.

(13) Miertuš, S.; Scrocco, E.; Tomasi, J. *Chem. Phys.* **1981**, *55*, 117–129.

(14) Cammi, R.; Tomasi, J. *J. Comput. Chem.* **1995**, *16*, 1449–1458.

(15) Grimme, S.; Antony, J.; Ehrlich, S.; Krieg, H. *J. Chem. Phys.* **2010**, *132*, 154104.

(16) Hratchian, H. P.; Schlegel, H. B. In *Theory and Applications of Computational Chemistry*; Dykstra, C. E., Frenking, G., Kim, K. S., Scuseria, G. E., Eds.; Elsevier: Amsterdam, 2005; pp 195–249.

(17) Gleadening, E. D.; Bakenhoop, J. K.; Reed, A. E.; Carpenter, J. E.; Bohmann, J. A.; Morales, C. M.; Landis, C. R.; Weinhold, F. Theoretical Chemistry Institute, University of Wisconsin, Madison 2013.

(18) Neese, F. *WIREs Comput Mol Sci* **2018**, *8*, e1327.

(19) Weigend, F.; Ahlrichs, R. *Phys. Chem. Chem. Phys.* **2005**, *7*, 3297–3305.

(20) Neese, F.; Wennmohs, F.; Hansen, A.; Becker, U. *Chem. Phys.* **2009**, *356*, 98–109.

(21) Adamo, C.; Barone, V. *J. Chem. Phys.* **1999**, *110*, 6158–6170.
